# The Immune Subtypes and Landscape of Advanced-Stage Ovarian Cancer

**DOI:** 10.3390/vaccines10091451

**Published:** 2022-09-02

**Authors:** Minjie Zhang, Mengna Shi, Yang Yu, Jianmin Sang, Hong Wang, Jianhong Shi, Ping Duan, Renshan Ge

**Affiliations:** 1 Department of Obstetrics and Gynecology, The Second Affiliated Hospital and Yuying Children’s Hospital of Wenzhou Medical University, Wenzhou 325027, China; 2 Department of Anesthesiology, The Second Affiliated Hospital and Yuying Children’s Hospital of Wenzhou Medical University, Wenzhou 325027, China; 3 State Key Laboratory of Molecular Oncology, National Cancer Center/National Clinical Research Center for Cancer/Cancer Hospital, Chinese Academy of Medical Sciences and Peking Union Medical College, Beijing 100021, China

**Keywords:** ovarian cancer, immune subtypes, immune landscape, immunotherapy

## Abstract

Immunotherapy has played a significant role in the treatment of a variety of hematological and solid tumors, but its application in ovarian cancer (OC) remains unclear. This study aimed to identify immune subtypes of OC and delineate an immune landscape for selecting suitable patients for immunotherapy, thereby providing potent therapeutic targets for immunotherapy drug development. Three immune subtypes (IS1–IS3) with distinctive molecular, cellular, and clinical characteristics were identified from the TCGA and GSE32062 cohorts. Compared to IS1, IS3 has a better prognosis and exhibits an immunological “hot”. IS3, in contrast, exhibits an immunological “cold” and has a worse prognosis in OC patients. Moreover, gene mutations, immune modulators, CA125, CA199, and HE4 expression, along with sensitivity either to immunotherapy or chemotherapy, were significantly different among the three immune subtypes. The OC immune landscape was highly heterogeneous between individual patients. Poor prognosis was correlated with low expression of the hub genes CD2, CD3D, and CD3E, which could act not only as biomarkers for predicting prognosis, but also as potential immunotherapy targets. Our study elucidates the immunotyping and molecular characteristics of the immune microenvironment in OC, which could provide an effective immunotherapy stratification method for optimally selecting patients, and also has clinical significance for the development of new immunotherapy as well as rational combination strategies for the treatment of OC patients.

## 1. Introduction

Immunotherapy has become a powerful clinical strategy for the treatment of patients with a broad variety of hematological and solid malignancies, including melanoma, non-small cell lung cancer, and bladder cancer [1]. Currently, immune checkpoint (ICP) inhibitors, such as antibodies blocking PD-1/PD-L1 axis, have shown significant antitumor activity in many cancer types and have been approved by the FDA as a recommended therapy for cancer treatment [2]. However, immunotherapy has been reported with a low response rate of clinical outcomes, and the overall survival (OS) benefit is limited in a narrow spectrum of patients, suggesting that there may be a specific pattern associated with clinical immunotherapy benefits. In the KEYNOTE-028 Phase-1b study of pembrolizumab in 26 heavily pre-treated ovarian cancer (OC) patients, the results showed one complete response, two partial responses, and six patients with stable disease, corresponding to a disease control rate of 34.6% [3]. Commonly, the efficacy of ICP inhibitors can be predicted based on tumor PD-L1 expression or tumor mutation burden, but these factors fail to determine whether the patients can benefit from the treatment or not [4]. Therefore, identification of immune subtypes and delineation of the immune landscape could be beneficial for improving the response and efficacy of immunotherapy in cancer treatment.

OC is a malignant tumor with the highest mortality rate among female reproductive system tumor tumors [5]. Approximately 75% of OC patients are at an advanced stage at the time of diagnosis. Additionally, most patients relapse, and the 5-year survival rate is less than 35% [6,7]. At present, ICP blockade therapy is the most promising immunotherapy for OC. However, a low response rate is often observed for ICP blockade therapy when used alone, while immunotherapy in combination with other therapeutic approaches could improve the response rate. The immunotherapy efficacy for OC is still unsatisfactory, due to the tumor heterogeneity, lack of antigen targets in the tumor microenvironment as well as human lymphocyte antigen expression, high expression of immunosuppressive molecules, and low infiltration of immune cells [8]. Specifically, an effective response to immunotherapy often depends on the interaction between tumor cells and the regulation of the tumor immune microenvironment (TIM). The TIM plays an essential role in either suppressing or enhancing the immune response by regulating different immune cell populations or functions [9]. Importantly, recent studies have shown that immunosuppression in the TIM is a major obstacle for effective immunotherapy in OC treatment [10]; however, a few studies have provided a comprehensive immune characterization of OC to help improve the immunotherapy efficacy for OC patients.

In this study, we performed retrospective analysis and identified three reproducible OC immune subtypes. Then, we selected one independent cohort for subtype verification and comprehensive molecular identification. Generally, the results showed that significant differences were observed for the prognosis of patients with different immune subtypes. In addition, each immune subtype was associated with a specific gene expression profile, while different subtypes had distinctive epigenetics, tumor infiltrating immune cell (TIIC) composition and immune function (immune activation or suppression), and cytokine characteristics. This study established a conceptual framework for understanding the TIM of OC, which not only provides insights for the exploration of novel and effective immunotherapies, but also facilitates the effective stratification and selection of OC patients for individual cancer treatments.

## 2. Materials and Methods

### 2.1. Data Extraction and Preprocessing

We obtained clinical information and transcriptome data of 338 OC patients from the The Cancer Genome Atlas (TCGA) database using the following screening criteria: 1. samples of the primary solid tumor were retained; 2. samples with stage III and stage IV were retained; 3. samples with OS time less than 30 days were removed; 4. genes with an expression level (TPM) equal to 0 in more than 50% of the samples were removed; 5. log transformation log2 (TPM + 1) was performed. Normalized mRNA matrix and clinical information of 260 OC patients were collected from Gene Expression Omnibus (GEO) dataset GSE32062 (https://www.ncbi.nlm.nih.gov/geo/query/acc.cgi?acc=GSE32062, accessed on 12 July 2022) using the following processing: 1. convert the probe to gene symbol according to the comment file; 2. remove probes corresponding to multiple gene names 3. samples with stage III and stage IV were retained; 4. samples with OS time less than 30 days were removed.

### 2.2. Generation of the Immune-Related Gene List

According to previous reports [11,12], the following categories of genes were collected as immune-related genes (IRGs) for subsequent analysis: immune cell-specific genes derived from single-cell RNA-seq data; costimulatory and coinhibitory molecules; cytokines and cytokine receptors; genes involved in antigen processing and presentation; and other IRGs. Following the criteria, a total of 2006 IRGs (Supplementary Materials File S1) were eventually collected.

### 2.3. Identification and Validation of the Immune Subtypes

After the previous screening, the expression data of 1933 IRGs were ultimately obtained. ConsensusClusterPlus was used to construct a consistency matrix, and the samples were classified by clustering [13]. The partition around medoids algorithm using the 1-Pearson correlation distance metric was applied, and 500 bootstraps were performed, each involving 80% of the patients in the TCGA cohort. The clustering number was set from 2 to 10, and the optimal classification was determined by calculating the consistency matrix and consistency cumulative distribution function. The immune subtypes were then validated in an independent cohort (GSE32062) with the same settings.

### 2.4. Characteristics of TIICs of the Immune Subtypes

For the calculation enrichment score of different TIICs in each sample, we used single-sample gene set enrichment analysis (ssGSEA) via GSVA package [14]. The list of 28 TIICs was derived from the previous literature reported by Charoentong et al. [15]. Then, an R package heatmap was used to show the degree of immune infiltration of samples in each subgroup and the relative abundance enrichment score of each immune cell type was measured and standardized from −4 to 4. The Euclidean distance was used to calculate the clustering between immune cells, and hierarchical clustering was used for cluster analysis of these immune infiltrating cells. When the number of classifications was 4, the differences between different categories could be obviously observed. Violin plots were drawn by vioplot and limma package to compare the differences of TIICs enrichment score among different subtypes. R package TCGAbiolinks was applied to analyze the correlation between immune subtypes and TCGA molecular subtypes [16,17]. The relationship between the immune subtypes and 56 previously reported immune-related molecular and cellular features [18] was evaluated by ANOVA algorithm to validate the reliability of the immune subtype. FDR < 0.01(Benjamini–Hochberg corrected *p* value) was used as the screening threshold, then 26 immune-related molecular and cellular features with significant differences were identified.

### 2.5. Prediction of the Efficacy of Immunotherapy and Chemotherapy

The combined subclass mapping of gene expression in the TCGA cohort was conducted to predict the clinical response of OC immune subtypes to immunotherapy and chemotherapy drugs. Based on the largest publicly available pharmacogenomics database (Genomics of Drug Sensitivity in Cancer), the chemotherapy response of each sample was tested. Four commonly used OC drugs, including cisplatin, paclitaxel, gemcitabine, and gefitinib, were selected for this study. The relative IC50 of each drug was predicted by the pRRophetic R package.

### 2.6. Assessment of the Immune Landscape

Considering the dynamic characteristics of the immune system in the TIM, graph learning-based dimensionality reduction analysis was performed by using the reduceDimension function of the monocle package with a gaussian distribution. Briefly, this analysis projects high-dimensional gene expression data into a tree structure in a low-dimensional space by preserving local geometric information [19] and has previously been used to model cancer progression and define trajectories using large-scale and single-cell gene expression data [20,21]. In this study, we conducted this analysis with IRG expression profiles. This immune landscape reflected the relationship between patients in a nonlinear manifold, which may complement the discrete immune subtypes defined in linear euclidean spaces.

### 2.7. Construction of Co-Expression Modules of OC

The weighted gene co-expression network analysis (WGCNA) algorithm implemented in the R software package was applied to recognize the co-expression modules of IRGs [22]. To establish a weighted adjacency matrix, the soft threshold was set at three, using the scale-free topology criterion. Next, the expression matrix was transformed into an adjacent matrix, and then the adjacent matrix was transformed into a topological matrix. According to the standard of a hybrid dynamic shear tree, the average linkage hierarchy clustering approach was used with a minimum of 30 genes for each network. The eigengenes of each module were computed and the close modules were integrated into a new one. Then, Kyoto Encyclopedia of Genes and Genomes (KEGG) enrichment analyses of genes in the module were used to explore gene functions and pathways through the clusterProfiler R package. Benjamini–Hochberg adjusted *p*-value less than 0.05 was taken to present statistical significance.

### 2.8. Tissue Specimens

This study was approved by the Second Affiliated Hospital and Yuying Children’s Hospital of Wenzhou Medical University. (LCKY2019-274). Signed informed consent forms, which were provided by the Wenzhou Medical University, were obtained from all participants. No patients received treatment before surgery. The fresh OC tissues were collected from the Second Affiliated Hospital and Yuying Children’s Hospital of Wenzhou Medical University between 2018 and 2020. The primary tumor tissues were isolated from each patient, evaluated by an experienced pathologist, and immediately stored in liquid nitrogen for further analysis. In this study, we defined patients who relapsed within one year as the recurrence group, and defined patients who did not relapse for more than one year as the no recurrence group.

### 2.9. Total RNA Extraction, Quantitative Real-Time PCR and Western Blotting

Total RNA extraction, quantitative real-time PCR (qRT-PCR) analysis and western blotting were performed as previously described [23]. The primer sequences are shown in Supplementary Materials File S2, GAPDH served as an internal control. The antibodies used were as follows: anti-CD2 antibody (Abcam, Cambridge, UK), anti-CD3D antibody (Abcam, Cambridge, UK), anti-CD3E antibody (Cell Signaling Technology, Danvers, MA, USA), and anti-GAPDH antibody (Proteintech, Wuhan, China).

### 2.10. Statistical Analysis

Kaplan–Meier (KM) analysis with the log-rank test was performed to evaluate OS differences among groups. Student’s *t*-test was used to determine the significance of differences between two groups, and ANOVA was conducted for comparisons among more than two groups. Bonferroni, Benjamini–Hochberg, and FDR methods were used to derive significance measures for post hoc tests. All of the above statistical analyses were performed using R version 3.5.3 and SPSS 22.0 software (SPSS Inc. Chicago, IL, USA).

## 3. Results

### 3.1. Identification and Validation of the Immune Subtypes

**Figure 1:**
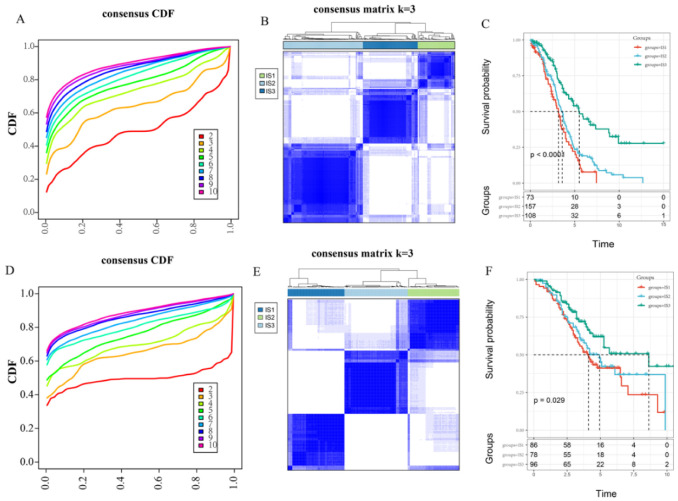
Identification and validation of immune subtypes. (**A**). CDF curve for the TCGA cohort. (**B**). Sample clustering heatmap for the TCGA cohort. (**C**). KM curves showing the OS of the OC immune subtypes in the TCGA cohort. (**D**). CDF curve for the GSE32062 cohort. (**E**). Sample clustering heatmap for the GSE32062 cohort. (**F**). KM curves showing the OS of the OC immune subtypes in the GSE32062 cohort.

Accumulated evidence have suggested that immunotyping can reflect the comprehensive immune status in the tumors and their microenvironment, and identification of specific populations might improve the efficacy of immunotherapy for patients [24]. Firstly, we extracted the expression profiles of IRGs in OC from the TCGA RNA-seq data, and obtained 1933 genes for further analysis. Meanwhile, ConsensusClusterPlus was used to cluster 338 OC samples. According to the cumulative distribution function and clustering heatmap, the optimal number of clusters was 3 (Figure 1A,B). Therefore, we defined the three immune subtypes as IS1 (n = 73), IS2 (n = 157), and IS3 (n = 108). IS3 showed the best prognosis among three immune subtypes, whereas IS1 showed the poorest prognosis (Figure 1C). In addition, the same approach for immune subtype analysis was conducted on GSE32062 chip data. Similarly, the 260 samples obtained could also be divided into three immune subtypes (Figure 1D,E). The prognoses of these three immune molecular subtypes were also significantly different (*p* < 0.05), which was consistent with those from the TCGA dataset (Figure 1F). The significant prognostic impact of the three immune subtypes in both cohorts indicated that immunotyping could be used as a precise prognostication strategy for OC.

### 3.2. Hub Mutation Gene Features in Different Immune Subtypes

**Figure 2:**
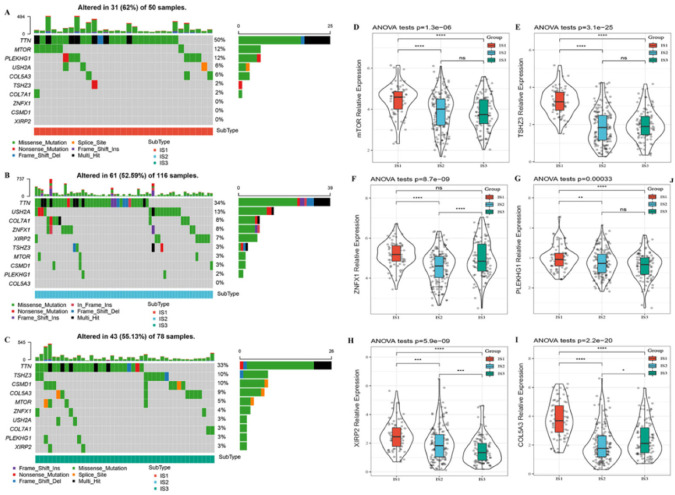
Hub mutation gene features in different immune subtypes. **A–C**. Top 10 mutation gene maps for the three immune subtypes: IS1 (**A**), IS2 (**B**), and IS3 (**C**). **D–I**. Differential expression of mutation genes among three immune subtypes: mTOR (**D**), TSHZ3 (**E**), ZNFX1 (**F**), PLEKHG1 (**G**), XIRP2 (**H**), and COL5A3 (**I**). * *p* < 0.05; ** *p* < 0.01; *** *p* < 0.001; **** *p* < 0.0001, and ns, no significance.

In order to explore hub mutation gene features in different immune subtypes, we used the TCGA mutation dataset processed by Mutect2 to calculate mutations in each patient and screened out 1901 genes with a cumulative mutation frequency greater than three in all three subtypes (Supplementary Materials File S3). The chi-square test was used to identify genes with significantly high mutation frequency. The selection threshold was multiple-test adjusted *p* < 0.05, and 136 genes were ultimately obtained (Supplementary Materials File S4). We conducted statistical analysis of the mutations of the top 10 mutant genes of the three subtypes and found that the mutation rate of IS1, IS2, and IS3 was 62%, 52.59%, and 55.13%, respectively (Figure 2A–C). Furthermore, six mutated genes (mTOR, TSHZ3, ZNFX1, PLEKHG1, XIRP2, and COL5A3) with different expression among the three immune subtypes were shown in Figure 2D–I. Interestingly, we found that all six mutated genes were expressed the highest in the IS1 immune subtype, which had the worst prognosis (Figure 1C,F). Previous studies have demonstrated that hub gene mutations and tumor-initiating mutations play key roles in tumor immune escape and progression [25,26,27]. Our analysis also suggested that the difference in prognoses among the three immune subtypes may be related to the heterogeneity of mutation frequency and expression of hub genes.

### 3.3. Different Expression of Immune Modulators among Three Immune Subtypes

**Figure 3:**
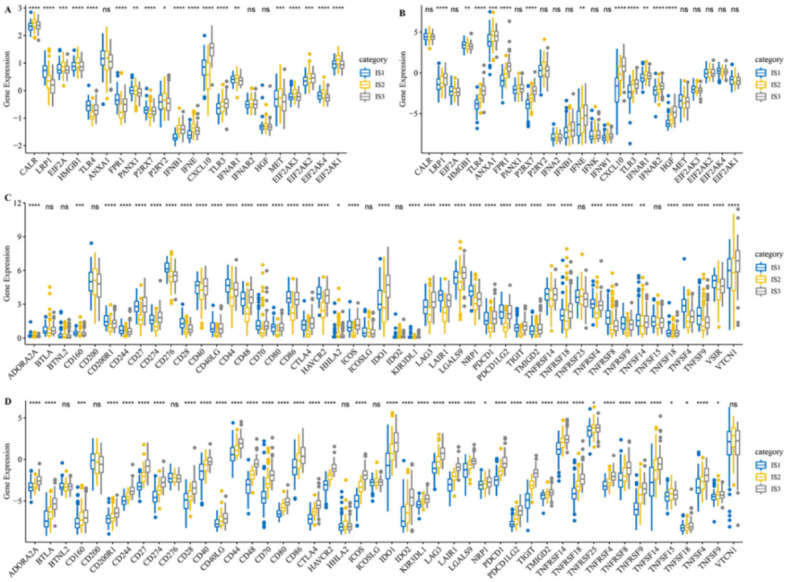
Different expression of ICDMs and ICPs among three immune subtypes. (**A**,**B**) Differential expression of ICDMs among the OC immune subtypes in the TCGA cohort (**A**) and GSE32062 cohort (**B**). (**C**,**D**) Differential expression of ICPs among the OC immune subtypes in the TCGA cohort (**C**) and GSE32062 cohort (**D**). * *p* < 0.05; ** *p* < 0.01; *** *p* < 0.001; **** *p* < 0.0001; and ns, no significance.

Since immunogenic cell death modulators (ICDMs) and ICPs play pivotal roles in tumor immunity, we next analyzed their expression levels among the different subtypes in the TCGA and GSE32062 cohorts. A total of 25 ICDMs were acquired from published literature [12] and 22 ICDMs were expressed in the TCGA cohort after matrix processing, while 19 of them (86.4%) were differently expressed among the immune subtypes (Figure 3A). By contrast, all 25 ICDMs were expressed in the GSE32062 cohort, and 12 ICDMs (48%) were differently expressed among the immune subtypes (Figure 3B). We concluded that about half or more ICDM expressions differed between three immune subtypes of both two cohorts. Besides, 47 ICPs were obtained from previous studies [28] and detected in both cohorts. The expression of 40 (85.1%) of these ICPs in the TCGA cohort (Figure 3C) and 41 (87.2%) of these ICPs in the GSE32062 cohort (Figure 3D) were different between the immune subtypes. For instance, CD200R1, CD244, CD276, CD28, CD40, CD44, CD80, CD86, HAVCR2, LAIR1, NRP1, PDCD1LG2, TNFRSF14, TNFRSF4, TNFRSF8, TNFRSF9, TNFSF14, TNFSF4, TNFSF9, and VSIR were upregulated in the IS1 subtype in the TCGA cohort. These results indicate that the different expression of ICDMs and ICPs in each immune subtype may lead to differences in disease progression, thus providing new clues for revealing immune heterogeneity in different subtypes.

### 3.4. Association between Immune Subtypes and Tumor Biomarkers

**Figure 4:**
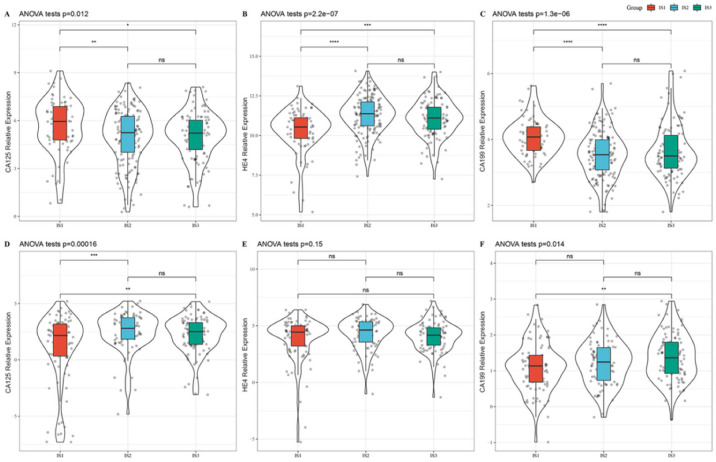
Association between immune subtypes and tumor biomarkers. (**A**–**C**) CA125 (**A**), HE4 (**B**) and CA199 (**C**) expression among three immune subtypes in the TCGA cohort. (**D**–**F**) CA125 (**D**), HE4 (**E**) and CA199 (**F**) expression among three immune subtypes in the GSE32062 cohort. * *p* < 0.05; ** *p* < 0.01; *** *p* < 0.001; **** *p* < 0.0001, and ns, no significance.

CA125, HE4, and CA199 are common tumor biomarkers for clinical diagnosis and treatment of OC, and their abnormal activation often indicates disease progression and poor prognosis [29]. Therefore, we next extracted the expression profiles of the genes encoding CA125, HE4, and CA199 from the TCGA and GSE32062 cohorts and analyzed their expression among the three subtypes. The results showed that the IS1 subtype with the worst prognosis had the highest expression of CA125 and lowest expression of HE4 in the TCGA cohort (Figure 4A,B), while it demonstrated the lowest expression of CA125 in the GSE32062 cohort (Figure 4D). Besides, the IS1 subtype showed slightly lower expression of HE4 and CA199 in the GSE32062 cohort when compared to the other two subtypes (Figure 4E,F). On the contrary, the IS3 subtype with the best prognosis had the relatively higher expression of CA199 in the GSE32062 cohort (Figure 4F). For precise prognostication of OC, these results suggested that simply assessing CA125, HE4, and CA199 expression for predicting patient prognosis might not be as effective as immunotyping (Figure 1C,F).

### 3.5. Characteristics of TIICs of the Immune Subtypes

**Figure 5:**
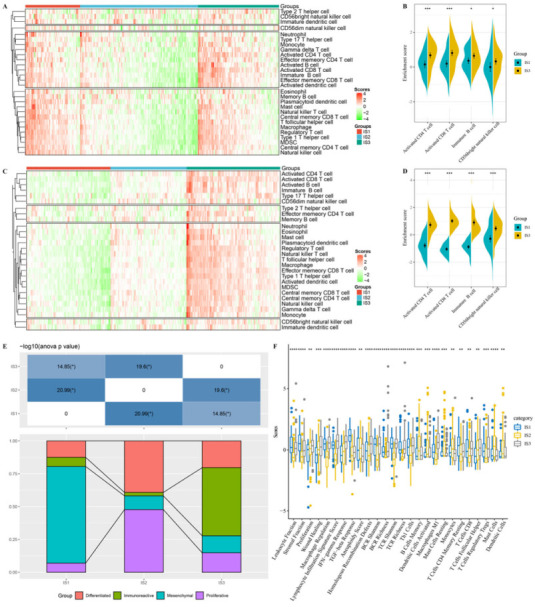
Characteristics of TIICs of the immune subtypes. (**A**,**C**). Differential enrichment scores of 28 TIICs among three subtype in the TCGA (**A**) and GSE32062 cohort (**C**). (**B**,**D**). Several immune cell enrichment scores in the TCGA cohort (**B**) and GSE32062 cohort (**D**) with significant differences between IS1 and IS3 subtypes. (**E**). Overlap of three immune subtypes with TCGA molecular typing of OC. (**F**). Enrichment scores of 26 immune-related characteristics with significant differences among three immune subtypes. * *p* < 0.05; ** *p* < 0.01; *** *p* < 0.001; **** *p* < 0.0001.

The response to immunotherapy depends on the immunological status of the tumor microenvironment. Previous studies have shown that the tumor infiltrates are composed of at least 28 TIICs. Therefore, we further characterized these TIICs among three immune subtypes using ssGSEA. In the TCGA cohort, the TIICs were mainly divided into four categories (Figure 5A). Particularly, the enrichment scores of activated CD4^+^ T cells (*p* < 0.001), activated CD8^+^ T cells (*p* < 0.001), immature B cells (*p* < 0.05), and CD56-bright natural killer cells (*p* < 0.05) in the IS1 subtype were lower than those in the IS3 subtype (Figure 5B). Interestingly, these results were also observed in the GSE32062 cohort (Figure 5C,D). Given that the IS1 subtype was associated with the worst prognosis, while the IS3 subtype showed the best prognosis, good prognosis of OC may be positively related to the infiltration degree of these four immune cells. Compared to IS3, the IS1 can be considered immunological “cold”, while the IS3 can be considered immunological “hot”. Therefore, OC patients with IS3 tumors may respond positively to immunotherapy.

In the TCGA dataset, OC samples had been divided into four molecular subtypes: immunoreactive, mesenchymal, proliferative, and differentiated. To demonstrate the reliability of the immunotyping, we explored the relationship between the three immune subtypes and the previously reported TCGA molecular subtypes. Among the four subtypes, the patients with the IS1 subtype were mainly enriched in the mesenchymal subtype, while patients with the IS3 subtype were mainly enriched in the immunoreactive subtype (Figure 5E). Moreover, immunoreactive subtype patients have a relatively good prognosis among the four molecular subtypes [30], which is consistent with previous analysis (Figure 1C). In addition, we explored the association between the immune subtypes with 56 previously defined immune molecular features and identified 26 significantly different molecular features (FDR < 0.01, Figure 5F). IS3 had the highest scores in terms of macrophage regulation, lymphocyte infiltration signature score, IFN-gamma response, Th1 cells, CD8^+^ T cells, and follicular helper T cells. These features were associated with an immune-activated phenotype, which was mutually verified with the conclusion that the IS3 subtype was mainly enriched in the immunoreactive subtype and was immunological “hot”. Overall, these findings further confirm the efficacy and prognostic value of our immune subtype. Therefore, immunotyping is helpful for understanding the immune status of OC patients, and thus could be used to classify patients into immunological “hot” or immunological “cold”, which potentially could guide clinicians to assess the feasibility of these patients for immunotherapy.

### 3.6. Association between Immune Subtypes and Immunotherapy/Chemotherapy Response

**Figure 6:**
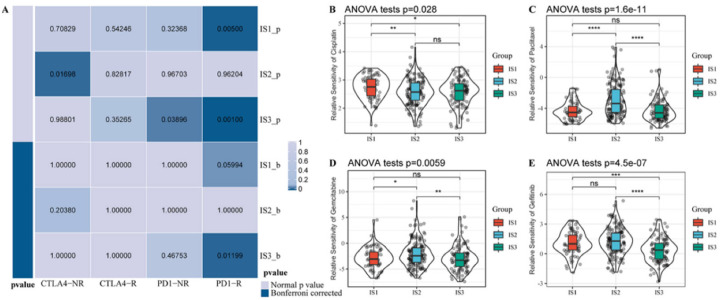
Association between immune subtypes and immunotherapy/chemotherapy response. (**A**). Submap analysis showing that IS3 is potentially more sensitive to PD-1 inhibitors. Bonferroni adjusted *p*-values less than 0.05 were considered statistically significant. B-E. Box plots of the estimated IC50 values for cisplatin (**B**), paclitaxel (**C**), gemcitabine (**D**), and gefitinib (**E**). * *p* < 0.05; ** *p* < 0.01; *** *p* < 0.001; **** *p* < 0.0001, and ns, no significance.

Considering that immunotherapy and chemotherapy are important strategies for the treatment of OC, we further assessed the response of different immune subtypes to these two clinical treatments. Using subclass mapping, we compared the TCGA expression profiles of the three immune subtypes with another published dataset (GSE91061) from 47 patients with melanoma who received PD-1 inhibitors or cytotoxic T-lymphocyte-associated protein-4 inhibitors [31]. Bonferroni corrected *p*-values less than 0.05 were considered statistically significant, and a significant correlation was observed between the expression profile of the IS3 group and the PD1-response group (Bonferroni corrected *p* < 0.05, Figure 6A), suggesting that the IS3 subtype is more sensitive to PD-1 inhibitors than the other two subtypes. In terms of response to chemotherapeutic drugs, IS1 was less sensitive to cisplatin (Figure 6B). Additionally, IS2 was less sensitive to paclitaxel and gemcitabine among the three subtypes (Figure 6C,D) and IS3 was more sensitive to gefitinib among the three subtypes (Figure 6E). These data indicated that immunophenotyping could provide novel insight and guidance for immunotherapy and chemotherapy drug selection for OC patients, which would further promote the individualized treatment of OC patients.

### 3.7. Immune Landscape of OC in the TCGA Cohort

**Figure 7:**
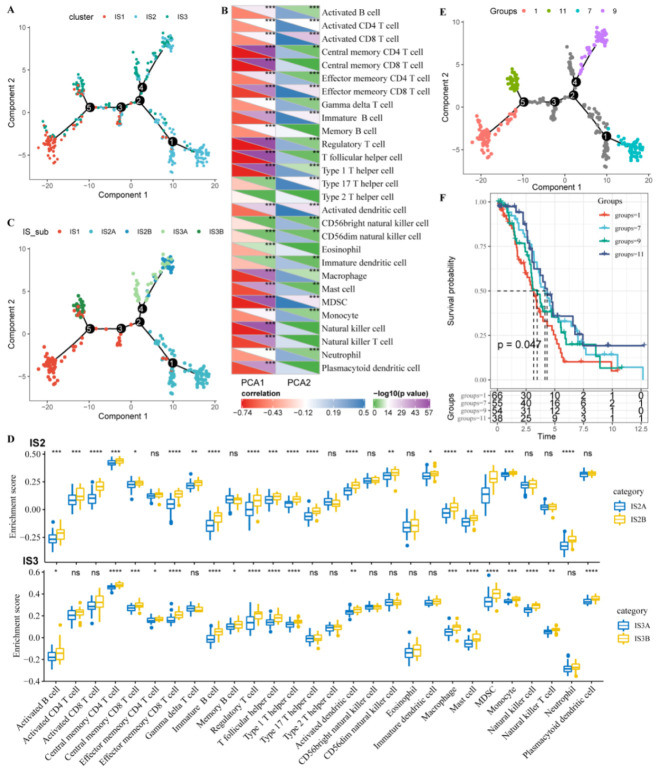
Immune landscape of OC. (**A**). Immune landscape of OC. Each point represents a sample, different colors represent different molecular subtypes, the horizontal axis represents the first principal component, and the vertical axis represents the second principal component. (**B**). Heatmap of two principal components with 28 TIICs. (**C**). Immune landscape of the subsets of OC immune subtypes. (**D**). Differential enrichment scores of the 28 TIICs in the above subsets. (**E**). Immune landscape of OC. (**F**). Prognostic differences based on the OC immune landscape. * *p* < 0.05; ** *p* < 0.01; *** *p* < 0.001; **** *p* < 0.0001, and ns, no significance.

To visualize individual patient distribution, we placed a single patient into a graph with a sparse tree structure and defined the OC immune landscape in the TCGA cohort. The position of the patient represented the overall characteristics of the immune microenvironment of the corresponding tumor subtype (Figure 7A). As shown in Figure 7B, the horizontal axis (the first principal component) was highly correlated with a variety of immune cells, including central memory CD8^+^ T cells, regulatory T cells, follicular helper T cells, central memory CD4^+^ T cells, natural killer cells, myeloid-derived suppressor cells (MDSCs), and type 1 T helper cells, and macrophages had the highest correlation (|R| > 0.7). The vertical coordinate (the second principal component) was well correlated with activated CD8^+^ T cells, activated dendritic cells, and effector memory CD8^+^ T cells. These results suggested that there was significant intraclass heterogeneity in the subtypes. The IS2 and IS3 subtypes were further divided into two subsets, respectively (Figure 7C), and the enrichment scores of several immune cells were significantly different between the subsets (Figure 7D). For example, IS2A showed lower scores of activated B cells, activated CD4^+^ T cells, activated CD8^+^ T cells, effector memory CD8^+^ T cells, regulatory T cells, and MDSCs than those of IS2B, which suggested that IS2A is more like an immunological cold subtype than IS2B. In addition, samples with extreme distribution positions in the immune landscape were subjected to prognostic comparison, and patients in group 1 showed the worst survival probability (Figure 7E,F). This landscape analysis provided further complementary results for the immune subtypes.

### 3.8. Identification of Immune Gene Co-Expression Modules and Immune Hub Genes

**Figure 8:**
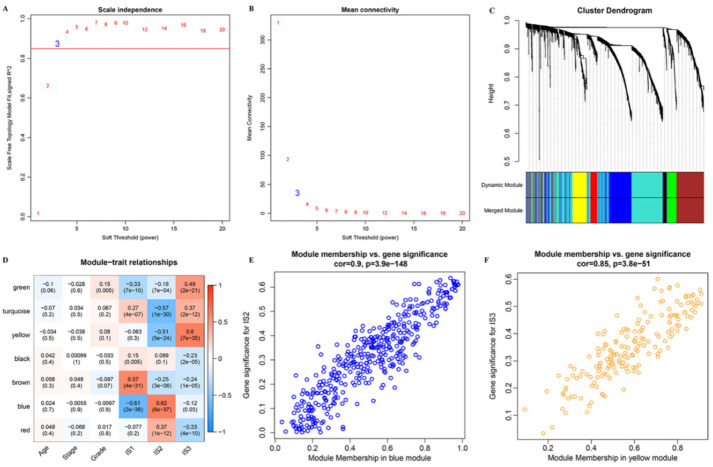
Identification of the immune gene co-expression module of OC. (**A**). Scale-free fit index for various soft-thresholding powers (β). (**B**). Mean connectivity for various soft-thresholding powers. (**C**). Dendrogram of all differentially expressed genes clustered; (**D**). Heatmap of the correlations between the 7 modules and three immune subtypes. (**E**): Scatter diagram of genes with significant correlations with the IS2 subtype in the blue module. (**F**): Scatter diagram of genes with significant correlations with the IS3 subtype in the yellow module.

An immune gene co-expression module can be used to explore which IRGs could significantly influence the efficacy of immunotherapy and OC prognosis. Therefore, the R package “WGCNA” was further utilized to identify the co-expression modules of these immune genes. The soft threshold was set at three in the scale-free network (Figure 8A,B). A total of seven modules (Supplementary Materials File S5) were obtained by setting height = 0.25, deep split = 2, and module size = 30 (Figure 8C). Then, we further analyzed the correlation between each module and patient age, stage, grade, IS1, IS2, and IS3 (Figure 8D). The blue module was dramatically correlated with the IS2 subtype (*p <* 0.001, Figure 8E), while the yellow module was dramatically related to the IS3 subtype (*p <* 0.001, Figure 8F).

**Figure 9:**
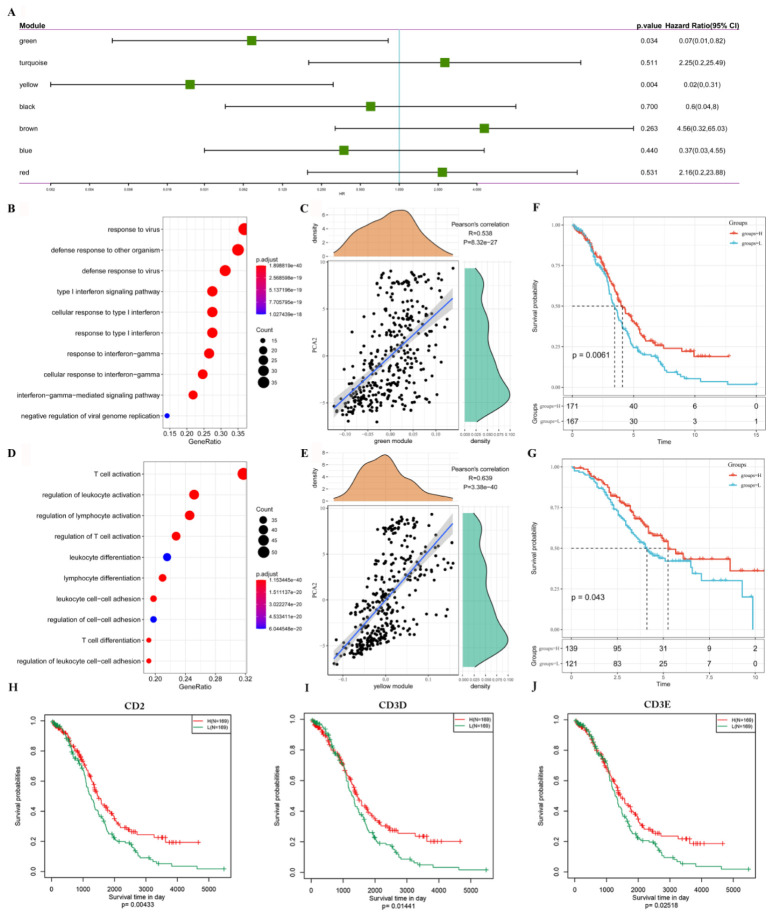
Identification of immune hub genes of OC. (**A**). Forest maps of single factor survival analysis of seven modules of OC. (**B**). Dot plot showing the top 10 KEGG terms in the green module. The dot color intensity and size represent the enrichment level and gene count respectively. (**C**). Correlation between the green module feature vector and the second principal component in the immune landscape. (**D**). Dot plot showing the top 10 KEGG terms in the yellow module. (**E**). Correlation between the yellow module feature vector and the second principal component in the immune landscape. (**F**,**G**). KM survival curve of patients grouped according to the expression of model characteristic genes in the yellow module in the TCGA cohort (**F**) and in the GSE32062 cohort (**G**). (**H**–**J**). KM survival curves of patients stratified by hub gene CD2 (**H**), CD3D (**I**), and CD3E (**J**) expression in the TCGA cohort.

Further prognostic correlation analysis showed that the green and yellow modules were associated with OC prognosis (Figure 9A). Moreover, the functional enrichment analysis showed that genes enriched in the green module were involved in immune-related pathways, such as response to virus and defense response to other organisms (Figure 9B); while the green module presented a dramatically positive correlation with the second principal component in the immune landscape (*p* < 0.001, Figure 9C). The yellow module was enriched with genes involved in T cell activation and also showed a dramatically positive correlation with the second principal component (*p* < 0.001) in the immune landscape (Figure 9D,E). As shown in Figure 7, the second principal component is positively correlated with Activated CD8^+^ T cell, Activated dendritic cell, and Effector memory CD8^+^ T cell. It was consistent with the result that the green and yellow modules are both positively correlated with the activation of the immune signal pathway and the second principal component.

Furthermore, we extracted the expression profiles of genes in the yellow module with the highest prognostic correlation coefficient (greater than 0.85) from the TCGA and GSE32062 cohorts. The mean value of expression was used as the sample feature, and the patients were classified by the mean value to analyze whether the patients with varied scores had a difference in prognosis. In the TCGA cohort, patients with high scores of genes that clustered into yellow modules had prolonged survival compared to those with low scores (Figure 9F). Moreover, similar results were observed in the GSE32062 cohort (Figure 9G). Finally, we selected yellow-module genes with a correlation of more than 0.9 as hub gene and identified three genes (Supplementary Materials File S6), CD2, CD3D and CD3E. In addition, KM survival analysis of the TCGA cohort revealed that low mRNA levels of CD2 (*p* < 0.01), CD3D (*p* < 0.05), or CD3E (*p* < 0.05) were positively correlated with the poor prognosis of patients (Figure 9H–J). It indicated that the hub genes can act as biomarkers for predicting the prognoses of OC patients as well as identifying patients for immunotherapy.

### 3.9. Verification of the Prognostic Characteristics of CD2, CD3D and CD3E in the ICGC Cohort and Independent Cohort

**Figure 10:**
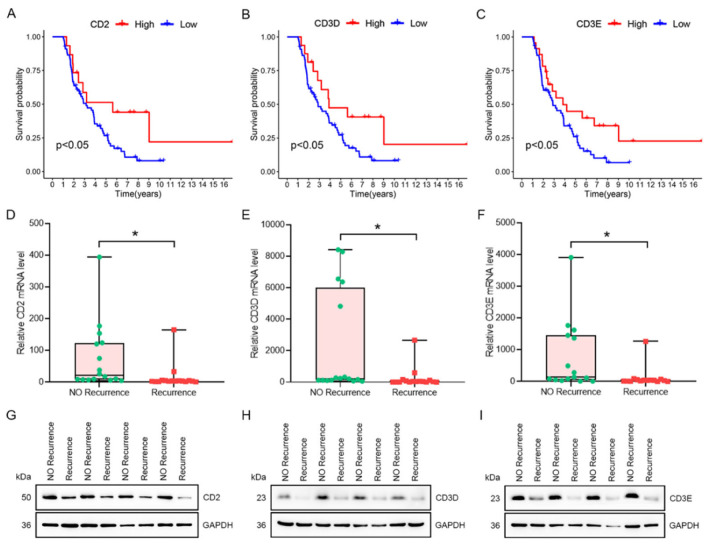
Verification of the prognostic characteristics of immune hub genes in the ICGC cohort and independent cohort. (**A**–**C**) KM survival curve of patients grouped according to the expression of CD2 (**A**), CD3D (**B**), and CD3E (**C**) in the ICGC database. (**D**–**F**). CD2 (**D**), CD3D (**E**), and CD3E (**F**) mRNA levels in tumor tissues from patients with and without recurrence. (**G**–**I**). CD2 (**G**), CD3D (**H**), and CD3E (**I**) protein levels in tumor tissues from patients with and without recurrence. * *p* < 0.05.

In order to explore the accuracy of the prognostic prediction of three hub genes, an ICGC dataset was downloaded for prognostic analysis. We further confirmed that low mRNA levels of CD2 (*p* < 0.05), CD3D (*p* < 0.05), or CD3E (*p* < 0.05) were significantly correlated with poor prognosis of OC patients in the ICGC cohort (Figure 10A–C). Then, the qRT-PCR and western blotting were applied to detect the expression differences of CD2, CD3D, and CD3E in tumor tissues from recurrence and no recurrence patients. The mRNA levels of CD2 (*p* < 0.05), CD3D (*p* < 0.05), or CD3E (*p* < 0.05) were significantly lower in patients with recurrence than those without recurrence (Figure 10D–F). Moreover, the change trend of three protein levels between two groups was consistent with the change of mRNA. As the patients in the recurrent group usually represent the worst prognosis, prognostic analysis in the ICGC cohort and experimental results in the independent cohort further validate our previous bioinformatics analysis.

## 4. Discussion

The TIM is highly complex and critical in the progression of OC [32], and immune infiltrating cells and immune-related genes play vital roles in diagnosis and treatment [33]. In this study, we analyze the public datasets that include TCGA and GEO cohorts to identify OC immune subtypes and delineate an immune landscape. Based on 1933 IRGs, ConsensusClusterPlus was applied to construct a consistency matrix and then three immune subtypes (IS1, IS2, and IS3) with significantly different prognoses were identified. The IS3 subtype showed the best prognosis, while the IS1 subtype had the worst prognosis among the three subtypes. Subsequently, we explored the hub gene mutations and expression characteristics of each subtype and we conducted a differential analysis of ICDMs and ICPs among the three subtypes. Not only can we intuitively find that there are significant differences in the mutation frequency and expression of frequently mutated genes among the three subtypes, but also that the mRNA levels of more than half of ICDMs or ICPs are significantly different. Since hub gene mutations, ICDMs, and ICPs play pivotal roles in tumor progression and immune response [34,35,36], the heterogeneities of the three are important inducers for the different prognoses of immune subtypes. Currently in clinical practice, CA125, HE4 and CA199 are widely used in the diagnosis of OC [37]. However, in this study it showed that the IS1 subtype with the worst prognosis had the highest expression of CA125 and lowest expression of HE4 in the TCGA cohort, but it exhibited the lowest expression of CA125 in the GSE32062 cohort. Moreover, the IS3 subtype with the best prognosis possessed the relatively higher expression of CA199 in the GSE32062 cohort. We concluded that there was an inconsistency in CA125 performance between TCGA and GEO cohorts, based on prognostic features, HE4 and CA199 possessed the opposite trend in expression as expected. However, the predictive effect of the immune subtypes was well validated in both TCGA and GEO datasets, so our typing may be more suitable for risk prediction in patients. Of course, the popularization and application of our immune subtypes still need extensive verification by a large multi-center cohort.

Previous studies have identified four molecular subtypes of OC (immunoreactive, mesenchymal, proliferative, and differentiated) based on TCGA dataset. In this study, the patients with the IS1 subtype were mainly enriched in the mesenchymal subtype, while patients with the IS3 subtype were mainly enriched in immunoreactive subtype. Our three immune subtypes were closely related to TCGA molecular subtypes. Generally, the tumor microenvironment in most of OC patients is immunosuppressed. T cells can effectively combat tumors by generating immunomodulatory antibodies after immune system activation [38], while immunotherapy for cancer treatment is based on the activation of tumor-reactive CD4^+^ and CD8^+^ T cells [39]. In the process of tumor immunity, CD8^+^ T cells are activated and transformed into effector CD8^+^ cytotoxic T cells to play a lasting and effective anti-tumor immune response [40]. Our study showed that the enrichment score of activated CD4^+^ T cells, activated CD8^+^ T cells, immature B cells, and CD56-bright natural killer cells in the IS1 subtype was lower than that in the IS3 subtype. Then, we concluded that the IS3 subtypes belonged to immune hot tumors, and IS1 belonged to immune cold tumors. Neoantigens are usually immunogenic only in the presence of infiltrating lymphocytes, so immune hot tumors with more infiltrating lymphocytes may be more sensitive to immune checkpoint inhibitors. In the follow-up analysis, we also confirmed that the IS3 subtype may have a higher response rate to PD-1 therapy. Since the establishment of immune subtypes can advance the understanding of tumorigenesis and provide the basis for precise clinical treatment [41], our immunophenotyping not only validates the accuracy of TCGA molecular typing, but also complements it and provides a new perspective for immunotherapy.

Seven co-expression modules were constructed using the WGCNA method in order to explore which IRGs could significantly influence the efficacy of immunotherapy and OC prognosis. Compared with other methods, WGCNA has multiple advantages as its analysis is mainly focused on the association between co-expression modules and immune subtypes, and the results possess higher reliability and biological significance [42]. Here, we confirmed that the yellow module was dramatically related to the IS3 subtype and was a protective factor of OC prognosis. Additionally, functional enrichment analysis showed that the yellow module was involved in T cell activation, regulation of lymphocyte activation, and regulation of T cell activation. In the previous analysis, we found that IS3 possessed the highest scores in terms of lymphocyte infiltration signature score, CD8^+^ T cells, and follicular helper T cells, while the enrichment scores of activated CD8^+^ T cells in the IS3 subtype were both higher in the TCGA and GEO datasets. The analysis conclusions of different plates can complement and verify each other, which not only shows that our analysis has good rationality and reliability, but also confirms the importance of yellow module genes in the immune landscape of OC. Subsequently, it showed that CD2, CD3D, and CD3E were hub genes in the yellow module. CD2 is a transmembrane glycoprotein of the immunoglobulin superfamily expressed on the surface of T cells, natural killer cells, thymocytes, and dendritic cells [43]. CD2 expression is closely related to clinicopathological features such as clinical stage and distant metastasis [44]. In this study, we showed that the decrease of CD2 mRNA level was related to the poor prognosis of patients, while the protein and mRNA levels of CD2 in the recurrence group were lower than those in the no recurrence group. CD3 is an important marker protein on the surface of T lymphocytes and can form a complex with the T cell receptor, thus mediating the recognition of antigens and signals by T lymphocytes [45]. CD3D is associated with ICPs and infiltrating immune cells, and its expression is positively correlated with the prognosis of colon cancer [46]. CD3E is often used as a target of multidrug therapy, and Huo et al. [47,48] reported that high expression of CD3E is associated with better prognosis in patients with OC. In our study, CD2, CD3D, and CD3E all belong to the yellow module, and their high mRNA levels are related to good prognosis of OC patients, which indicate that they can be used as biomarkers of OC; however, their function mechanisms and the development of targeted drugs still need to be further explored.

We identified three immune subtypes of OC patients from publically available databases and analyzed the immune microenvironment characteristics of these different subtypes. These three subtypes represented diverse prognostic characteristics, molecular features and immune response patterns. However, further investigations with more clinical samples from patients receiving immunotherapy are required in order to clarify the factors that affect the immune response. In conclusion, our research provides a conceptual framework for understanding the immune landscape of OC and possesses distinct clinical significance to guide the design of new immunotherapies as well as appropriate combination strategies.

## Data Availability

Both mRNA data and clinical information can be obtained from the TCGA and GEO database. The original contributions presented in the study are included in the article/supplementary material, further inquiries can be directed to corresponding author.

## Supplementary Materials

The following supporting information can be downloaded at: https://www.mdpi.com/article/10.3390/vaccines10091451/s1, Supplementary Materials File S1: 2006 immune-related genes obtained from literature. Supplementary Materials File S2: Primers for qRT-PCR analysis. Supplementary Materials File S3: 1901 genes with a cumulative mutation frequency greater than 3 in all three subtypes in the TCGA cohort. Supplementary Materials File S4: 136 genes with significantly high mutation frequency among three immune subtype in the TCGA cohort. Supplementary Materials File S5: 7 immune gene co-expression modules obtained by cluster analysis. Supplementary Materials File S6: List of module genes and module trait correlations.

## Abbreviations

ICP: immune checkpoint; OS: overall survival; OC: ovarian cancer; TIM: tumor immune microenvironment; TIIC: tumor infiltrating immune cell; TCGA: The Cancer Genome Atlas; GEO: Gene Expression Omnibus; IRG: immune-related gene; ssGSEA: single-sample gene set enrichment analysis; WGCNA: weighted gene co-expression network analysis; KEGG: Kyoto Encyclopedia of Genes and Genomes; qRT-PCR: quantitative real-time PCR; KM: Kaplan-meier; ICDMs: immunogenic cell death modulators.
