# Supplementary material for: The Immune Subtypes and Landscape of Advanced-Stage Ovarian Cancer
Source: Vaccines (Basel). 2022 Sep 2;10(9):1451. doi: 10.3390/vaccines10091451 (PMC9501495; doi:10.3390/vaccines10091451)
Supplement: Supplementary file 1 [file vaccines-10-01451-s001.zip › Supplementary Materials File/Supplementary Materials File S1.pdf]

GeneSymbol	GeneID
A2M	2
ABCA6	23460
ABCA8	10351
ABCA9	10350
ABCB1	5243
ABCC9	10060
ABCD2	225
ABI3	51225
ABI3BP	25890
ACAP1	9744
ACE	1636
ACHE	43
ACOXL	55289
ACP5	54
ACSL5	51703
ACSM5	54988
ACSS3	79611
ACTA2	59
ACTN1	87
ACVR1	90
ACVR1B	91
ACVR2A	92
ACVR2B	93
ACVRL1	94
ADAM12	8038
ADAM28	10863
ADAM6	8755
ADAM8	101
ADAMDEC1	27299
ADAMTS10	81794
ADAMTS12	81792
ADAMTS14	140766
ADAMTS16	170690
ADAMTS2	9509
ADAMTS4	9507
ADAMTS5	11096
ADAMTS9	56999
ADAMTSL2	9719
ADAP2	55803
ADAT1	23536
ADCY4	196883
ADCYAP1	116
ADORA2A	135
ADORA3	140
ADPRH	141
ADRA2A	150
ADRM1	11047
AEBP1	165
AFF3	3899
AGAP2	116986

AGTR1	185
AHCYL2	23382
AIF1	199
CRYBG2	55057
AIM2	9447
AKAP12	9590
AKAP2	11217
AKAP5	9495
AKNA	80709
ALDH1A1	216
ALDH3B1	221
ALOX5	240
ALOX5AP	241
ALOXE3	59344
ALPK2	115701
AMH	268
AMHR2	269
JAML	120425
AMIGO3	386724
AMPD1	270
AMPH	273
ANGPTL1	9068
ANGPTL2	23452
ANK2	287
ANKRD17	26057
ANKRD22	118932
ANKRD36BP1	84832
ANKRD44	91526
ANKRD55	79722
SOWAHD	347454
ANO6	196527
ANTXR1	84168
ANTXR2	118429
ANXA6	309
AOAH	313
AOC3	8639
AP1S2	8905
AP3B1	8546
APBB1IP	54518
APBB2	323
APLNR	187
APOBR	55911
APOBEC3A	200315
APOBEC3D	140564
APOBEC3G	60489
APOBEC3H	164668
APOC1	341
APOC2	344
APOE	348
APOL3	80833
APOL6	80830

AQP10	89872
AQP1	358
AQP9	366
AREG	374
ARHGAP15	55843
ARHGAP18	93663
ARHGAP22	58504
ARHGAP25	9938
ARHGAP30	257106
ARHGAP31	57514
ARHGAP4	393
ARHGAP6	395
ARHGAP9	64333
ARHGDIB	397
ARHGEF15	22899
ARHGEF37	389337
ARHGEF6	9459
ARID5A	10865
ARL6IP5	10550
ARRB1	408
ARRB2	409
ARRDC5	645432
ARSB	411
ART4	420
ASAH1	427
CLMP	79827
ASGR2	433
ASPN	54829
ASRGL1	80150
ASXL2	55252
ASXL3	80816
ATE1	11101
ATP10A	57194
ATP2A3	489
ATP8A1	10396
ATP8B4	79895
AVPR1A	552
AZGP1	563
B2M	567
B3GAT1	27087
BANK1	55024
BATF	10538
BATF2	116071
BATF3	55509
BCL2A1	597
BCL2L14	79370
BCL6B	255877
BDKRB2	624
BEND5	79656
BEX5	340542
BFSP2	8419

BGN	633
BHLHA15	168620
BHLHE22	27319
BHLHE41	79365
BICC1	80114
BIN2	51411
BIRC6	57448
BLK	640
BMP2	650
BMP2K	55589
BMP7	655
BMPR1A	657
BMPR1B	658
BMPR2	659
BNC2	54796
BST1	683
BST2	684
BTK	695
BTLA	151888
BTN2A2	10385
BTN3A1	11119
BTN3A2	11118
BTN3A3	10384
C10orf128	170371
VSIR	64115
VSTM4	196740
C10orf99	387695
C11orf21	29125
RUBCNL	80183
MEDAG	84935
CEP128	145508
SLIRP	81892
RTRAF	51637
C14orf2	9556
C15orf48	84419
C15orf53	400359
C16orf54	283897
MILR1	284021
SCIMP	388325
LDLRAD4	753
PEAK3	374872
C19orf38	255809
TRIR	79002
MCEMP1	199675
C19orf66	55337
C1orf116	79098
C1orf127	148345
GCSAML	148823
C1orf162	128346
PIK3CD-AS1	644997
C1orf228	339541

THEMIS2	9473
C1orf54	79630
C1QA	712
C1QB	713
C1QC	714
C1QTNF7	114905
LAMP5	24141
C2	717
RTP5	285093
TRABD2A	129293
C3	718
C3AR1	719
C4A	720
NDNF	79625
C5AR1	728
DCANP1	140947
CREBRF	153222
C5orf56	441108
ADTRP	84830
UQCC2	84300
C6orf132	647024
CCDC170	80129
C7	730
CPED1	79974
NUGGC	389643
ERCC6L2	375748
C9orf139	401563
CYSRT1	375791
CACNA1C	775
CACNA2D2	9254
CACNA2D4	93589
CALB2	794
CALD1	800
CALR	811
CAMK1	8536
CAMK4	814
CANX	821
CARD11	84433
CARD8	22900
CARD9	64170
CASP5	838
CASS4	57091
CAV1	857
CCDC102B	79839
CCDC141	285025
CCDC69	26112
CCDC80	151887
CCL11	6356
CCL13	6357
CCL14	6358
CCL15	6359

CCL1	6346
CCL16	6360
CCL17	6361
CCL18	6362
CCL19	6363
CCL20	6364
CCL21	6366
CCL22	6367
CCL23	6368
CCL24	6369
CCL25	6370
CCL26	10344
CCL2	6347
CCL28	56477
CCL3	6348
CCL3L1	6349
CCL3L3	414062
CCL4	6351
CCL4L1	388372
CCL5	6352
CCL7	6354
CCL8	6355
CCNT1	904
CCR10	2826
CCR1	1230
CCR2	729230
CCR3	1232
CCR4	1233
CCR5	1234
CCR6	1235
CCR7	1236
CCR8	1237
CCR9	10803
CCRL2	9034
CD14	929
CD160	11126
CD163	9332
CD163L1	283316
CD180	4064
CD19	930
CD1A	909
CD1B	910
CD1C	911
CD1D	912
CD1E	913
CD200	4345
CD200R1	131450
CD207	50489
CD209	30835
CD226	10666
CD22	933

CD244	51744
CD247	919
CD248	57124
CD274	29126
CD27	939
CD28	940
CD2	914
CD300A	11314
CD300C	10871
CD300E	342510
CD300LB	124599
CD300LF	146722
CD302	9936
CD33	945
CD34	947
CD36	948
CD37	951
CD3D	915
CD3E	916
CD3G	917
CD40	958
CD40LG	959
CD48	962
CD4	920
CD52	1043
CD53	963
CD58	965
CD5	921
CD68	968
CD6	923
CD69	969
CD70	970
CD72	971
CD74	972
CD7	924
CD79A	973
CD79B	974
CD80	941
CD84	8832
CD86	942
CD8A	925
CD8B	926
CD93	22918
CD96	10225
ADGRE5	976
CDC42SE2	56990
CDH11	1009
CDH20	28316
CDH3	1001
CDH5	1003
CDH6	1004

CDK15	65061
CDKL5	6792
CDSN	1041
CEACAM19	56971
CEACAM21	90273
CEACAM4	1089
ADA2	51816
CELF2	10659
CERKL	375298
CETP	1071
CFP	5199
CHAC1	79094
CHIT1	1118
CHN1	1123
CHRD	8646
CHRDL1	91851
CHRNA6	8973
CHST13	166012
CHST2	9435
CIITA	4261
CILP	8483
CISH	1154
CLCF1	23529
CLEC10A	10462
CLEC11A	6320
CLEC12A	160364
CLEC14A	161198
CLEC1A	51267
CLEC3B	7123
CLEC4A	50856
CLEC4D	338339
CLEC4E	26253
CLEC4G	339390
CLEC4M	10332
CLEC5A	23601
CLEC6A	93978
CLEC9A	283420
CLECL1	160365
CLIC2	1193
CLIC3	9022
CLIC5	53405
CLIP3	25999
CLNK	116449
CLOCK	9575
CLTB	1212
CMA1	1215
CMAHP	8418
CMKLR1	1240
CMPK2	129607
CNFN	84518
CNR2	1269

CNRIP1	25927
CNTF	1270
CNTFR	1271
COL10A1	1300
COL11A1	1301
COL12A1	1303
COL14A1	7373
COL15A1	1306
COL18A1	80781
COL1A1	1277
COL1A2	1278
COL6A5	256076
COL3A1	1281
COL4A1	1282
COL4A2	1284
COL5A1	1289
COL5A2	1290
COL5A3	50509
COL6A1	1291
COL6A2	1292
COL6A3	1293
COL6A6	131873
COL8A1	1295
COLEC12	81035
CORIN	10699
CORO1A	11151
COTL1	23406
CPA3	1359
CPNE5	57699
CPVL	54504
CPXM1	56265
CPZ	8532
CR1	1378
CR1L	1379
CR2	1380
CRCT1	54544
CREB1	1385
CREB3L1	90993
CREBL2	1389
CRISPLD2	83716
CRLF2	64109
CRTAM	56253
CRYBB1	1414
CSF1	1435
CSF1R	1436
CSF2	1437
CSF2RA	1438
CSF2RB	1439
CSF3	1440
CSF3R	1441
CSGALNACT:	55454

CSMD2	114784
CST7	8530
CTF1	1489
CTGF	1490
CTHRC1	115908
CTLA4	1493
CTSB	1508
CTSE	1510
CTSG	1511
CTSK	1513
CTSL	1514
CTSO	1519
CTSS	1520
CTSW	1521
CTSZ	1522
CTTNBP2	83992
CX3CL1	6376
CX3CR1	1524
CXCL10	3627
CXCL11	6373
CXCL12	6387
CXCL1	2919
CXCL13	10563
CXCL16	58191
CXCL2	2920
CXCL3	2921
CXCL5	6374
CXCL6	6372
CXCL9	4283
CXCR1	3577
CXCR2	3579
CXCR2P1	3580
CXCR3	2833
CXCR4	7852
CXCR5	643
CXCR6	10663
ACKR3	57007
CXorf21	80231
CXorf36	79742
CXorf65	158830
CYBA	1535
CYBB	1536
CYFIP2	26999
CYP1B1	1545
CYP27A1	1593
CYSLTR1	10800
CYSLTR2	57105
CYTH4	27128
CYTIP	9595
DAAM2	23500
DAB2	1601

DACT1	51339
DACT3	147906
ACKR1	2532
DBH	1621
DCBLD1	285761
DCHS1	8642
DCN	1634
DDI2	84301
DDR2	4921
DDX58	23586
DDX60	55601
DENND1C	79958
DENND2A	27147
DERL3	91319
DHRS1	115817
DHRS9	10170
DIXDC1	85458
DKK2	27123
DLC1	10395
DLL4	54567
DMKN	93099
DMXL2	23312
DNAH8	1769
DNAJC5B	85479
DOCK10	55619
DOCK11	139818
DOCK2	1794
DOCK4	9732
DOCK8	81704
DOK1	1796
DOK2	9046
DOK3	79930
DOK5	55816
DOK6	220164
DPEP1	1800
DPEP2	64174
DPP8	54878
DPT	1805
DSC1	1823
DSG1	1828
DSP	1832
DUOXA1	90527
DUSP16	80824
DUSP4	1846
DYSF	8291
E2F5	1875
EBF1	1879
EBF2	64641
EBI3	10148
ECM2	1842
ECSCR	641700

EDA	1896
EDA2R	60401
EDAR	10913
EDNRA	1909
EDNRB	1910
EFEMP2	30008
EFTUD2	9343
EGF	1950
EGFR	1956
EHD2	30846
ADGRL4	64123
EMCN	51705
EMILIN1	11117
EMILIN2	84034
ADGRE2	30817
ADGRE4P	326342
ENG	2022
ENO3	2027
ENOX1	55068
ENPEP	2028
ENPP2	5168
ENPP3	5169
ENPP4	22875
ENTPD1	953
EOMES	8320
EP300	2033
EPCAM	4072
EPO	2056
EPOR	2057
EPS8	2059
EPS8L1	54869
EPSTI1	94240
ERAP1	51752
ERAP2	64167
ERN1	2081
ERP27	121506
ESAM	90952
ETS1	2113
ETV3	2117
ETV7	51513
EVI2A	2123
EVI2B	2124
EVPL	2125
F13A1	2162
F2R	2149
F5	2153
FABP3	2170
FCMR	9214
FAM105A	54491
FAM107A	11170
PCED1B	91523

DENND6B	414918
FAM129C	199786
FAM13C	220965
FAM155A	728215
FAM168A	23201
FAM171B	165215
FAM177B	400823
TVP23A	780776
CCSER1	401145
FAM198B	51313
FAM19A5	25817
FAM25A	643161
FAM25BP	100132929
CALHM5	254228
CALHM6	441168
PIEZO2	63895
STRIP2	57464
FAM46B	115572
FAM46C	54855
FAM49A	81553
NXPE4	54827
MINDY2	54629
RIPOR2	9750
FAM78A	286336
FAM83A	84985
FAM92B	339145
FAP	2191
FAS	355
FASLG	356
FAT4	79633
FBLN2	2199
FBLN5	10516
FBN1	2200
FBP1	2203
FBXL7	23194
FBXO6	26270
FCAR	2204
FCER1A	2205
FCER1G	2207
FCER2	2208
FCGBP	8857
FCGR1A	2209
FCGR1B	2210
FCGR1CP	100132417
FCGR2A	2212
FCGR2B	2213
FCGR2C	9103
FCGR3A	2214
FCGR3B	2215
FCGRT	2217
FCN1	2219

FCRL1	115350
FCRL2	79368
FCRL3	115352
FCRL4	83417
FCRL5	83416
FCRL6	343413
FCRLA	84824
FERMT2	10979
FERMT3	83706
FGD2	221472
FGD3	89846
FGD5	152273
FGF14	2259
FGF7	2252
FGL2	10875
FGR	2268
FHL5	9457
FIBIN	387758
FICD	11153
VEGFD	2277
FILIP1L	11259
FKBP11	51303
FKBP7	51661
FLI1	2313
ANKRD36BP2	645784
FLT1	2321
FLT3	2322
FLT3LG	2323
FLT4	2324
FLVCR2	55640
FMNL1	752
FMNL3	91010
FMOD	2331
FN1	2335
FNBP1	23048
FNDC1	84624
FNIP2	57600
FOLR2	2350
FOXP3	50943
FPR1	2357
FPR2	2358
FPR3	2359
FRZB	2487
FSCN1	6624
FSTL1	11167
FSTL3	10272
FUCA1	2517
FUT7	2529
FYB1	2533
FYN	2534
FZD4	8322

GAB3	139716
GALM	130589
GALNT15	117248
GAPT	202309
GAS7	8522
GATA1	2623
GATA2	2624
GATA3	2625
GATM	2628
GBGT1	26301
GBP1	2633
GBP2	2634
GBP4	115361
GBP5	115362
GCSAM	257144
GDF5	8200
GFI1	2672
GFRA3	2676
GGT1	2678
GGT5	2687
GGTA1P	2681
GHR	2690
GHRL	51738
GIMAP1	170575
GIMAP2	26157
GIMAP4	55303
GIMAP5	55340
GIMAP6	474344
GIMAP7	168537
GIMAP8	155038
GIPC3	126326
GIT2	9815
GJA4	2701
GJA5	2702
GJB2	2706
GJB3	2707
GJB5	2709
GJD3	125111
GLIPR2	152007
GLIS3	169792
GLRX	2745
COLGALT2	23127
GLT8D2	83468
GMFG	9535
GMIP	51291
GMPR	2766
GNA15	2769
GNAI2	2771
GNG11	2791
GNG2	54331
GNG7	2788

GNGT2	2793
GNLY	10578
GNS	2799
GPBAR1	151306
GPC5	2262
GPC6	10082
GPIHBP1	338328
ADGRG5	221188
ADGRF4	221393
ADGRF5	221395
ADGRA2	25960
GPR132	29933
ADGRD1	283383
GPR137B	7107
GPR141	353345
GPR15	2838
GPR157	80045
GPR171	29909
GPR174	84636
GPR18	2841
GPR183	1880
GPR25	2848
GPR34	2857
GPR35	2859
GPR4	2828
GPR55	9290
GPR65	8477
GPR78	27201
GPR82	27197
GPR84	53831
GPRIN3	285513
GPSM3	63940
GRAP	10750
GRAP2	9402
GREM1	26585
GRIN3A	116443
GSDMA	284110
GTF2A1	2957
GUCY1A2	2977
GUCY1A3	2982
GVINP1	387751
GYPC	2995
GZMA	3001
GZMB	3002
GZMH	2999
GZMK	3003
GZMM	3004
HAMP	57817
HAPLN3	145864
HAVCR1	26762
HAVCR2	84868

HCG26	352961
HCK	3055
HCLS1	3059
HCP5	10866
HCST	10870
HDC	3067
HECW2	57520
HEPH	9843
HEPHL1	341208
HERC6	55008
HERPUD1	9709
HEYL	26508
HFE	3077
HGF	3082
HIC1	3090
HIPK3	10114
HIST1H2AE	3012
HIST1H2AG	8969
HIST1H2AM	8336
HIST1H3H	8357
HK3	3101
HLA-A	3105
HLA-B	3106
HLA-C	3107
HLA-DMA	3108
HLA-DMB	3109
HLA-DOA	3111
HLA-DOB	3112
HLA-DPA1	3113
HLA-DPB1	3115
HLA-DPB2	3116
HLA-DQA1	3117
HLA-DQA2	3118
HLA-DQB1	3119
HLA-DQB2	3120
HLA-DRA	3122
HLA-DRB1	3123
HLA-DRB5	3127
HLA-DRB6	3128
HLA-E	3133
HLA-F	3134
HLA-G	3135
HLA-H	3136
HLX	3142
HMCN1	83872
ARHGAP45	23526
HMSD	284293
HNMT	3176
HPGD	3248
HPGDS	27306
HRH2	3274

HS3ST1	9957
HS3ST2	9956
HSD11B1	3290
HSD17B14	51171
HSH2D	84941
HSP90AA1	3320
HSP90AB1	3326
HSPA12B	116835
HSPA1A	3303
HSPA1B	3304
HSPA1L	3305
HSPA2	3306
HSPA4	3308
HSPA5	3309
HSPA6	3310
HSPA8	3312
HTR2A	3356
HTRA3	94031
HTRA4	203100
HVCN1	84329
HYDIN	54768
ICAM1	3383
ICAM2	3384
ICAM3	3385
ICK	22858
ICOS	29851
ICOSLG	23308
IDO1	3620
IDO2	169355
IFFO1	25900
IFI27	3429
IFI30	10437
IFI35	3430
IFI44	10561
IFI44L	10964
IFI6	2537
IFIH1	64135
IFIT2	3433
IFIT3	3437
IFIT5	24138
IFITM1	8519
IFITM3	10410
IFNA13	3447
IFNA21	3452
IFNAR1	3454
IFNAR2	3455
IFNB1	3456
IFNE	338376
IFNG	3458
IFNGR1	3459
IFNGR2	3460

IFNK	56832
IFNW1	3467
IGDCC4	57722
IGF1	3479
JCHAIN	3512
IGLL1	3543
IGSF10	285313
IGSF21	84966
IGSF6	10261
IKZF1	10320
IKZF3	22806
IL10	3586
IL10RA	3587
IL10RB	3588
IL11	3589
IL11RA	3590
IL12A	3592
IL12B	3593
IL12RB1	3594
IL12RB2	3595
IL13	3596
IL13RA1	3597
IL15	3600
IL15RA	3601
IL16	3603
IL17A	3605
IL17B	27190
IL17RA	23765
IL17RB	55540
IL18	3606
IL18BP	10068
IL18R1	8809
IL18RAP	8807
IL19	29949
IL1A	3552
IL1B	3553
IL36G	56300
IL1R1	3554
IL1R2	7850
IL1RAP	3556
IL1RL1	9173
IL1RN	3557
IL20	50604
IL20RA	53832
IL20RB	53833
IL21	59067
IL21R	50615
IL22	50616
IL22RA1	58985
IL22RA2	116379
IL2	3558

IL23A	51561
IL23R	149233
IL24	11009
IL27	246778
IFNL2	282616
IFNLR1	163702
IFNL1	282618
IL2RA	3559
IL2RB	3560
IL2RG	3561
IL32	9235
IL3RA	3563
IL4	3565
IL4I1	259307
IL4R	3566
IL5	3567
IL5RA	3568
IL6	3569
IL6R	3570
IL6ST	3572
IL7	3574
IL7R	3575
CXCL8	3576
IL9	3578
IL9R	3581
INHBA	3624
INHBB	3625
INHBC	3626
INHBE	83729
INMT	11185
INPP5D	3635
IPCEF1	26034
IQGAP2	10788
IRF1	3659
IRF4	3662
IRF7	3665
IRF8	3394
IRF9	10379
ISG15	9636
ISG20	3669
ISLR	3671
ITGA11	22801
ITGA1	3672
ITGA2B	3674
ITGA4	3676
ITGA5	3678
ITGA8	8516
ITGA9	3680
ITGAD	3681
ITGAL	3683
ITGAM	3684

ITGAX	3687
ITGB1	3688
ITGB2	3689
ITGB3	3690
ITGB7	3695
ITGBL1	9358
ITK	3702
ITM2A	9452
IVL	3713
JAK2	3717
JAK3	3718
JAKMIP1	152789
JAM2	58494
JAM3	83700
JMY	133746
JSRP1	126306
JUP	3728
KCNA3	3738
KCNAB2	8514
KCND2	3751
KCNE4	23704
KCNH2	3757
KCNJ10	3766
KCNJ8	3764
KCNK13	56659
KCNK6	9424
KCNMB1	3779
KCNN3	3782
KCNN4	3783
KCNT2	343450
KCTD12	115207
KDR	3791
FAM30A	9834
KIAA0368	23392
TESPA1	9840
KIAA0754	643314
JCAD	57608
KIAA1549	57670
TLDC1	57707
SHISAL1	85352
KIAA1755	85449
KIF21B	23046
KIR2DL1	3802
KIR2DL3	3804
KIR2DL4	3805
KIR2DS4	3809
KIR3DL1	3811
KIR3DL2	3812
KIR3DL3	115653
KIRREL1	55243
KIT	3815

KITLG	4254
KL	9365
KLHDC10	23008
KLHL11	55175
KLHL23	151230
KLHL6	89857
KLK7	5650
KLK9	284366
KLRB1	3820
KLRC1	3821
KLRC2	3822
KLRC3	3823
KLRC4	8302
KLRD1	3824
KLRG1	10219
KLRK1	22914
KMO	8564
KRT1	3848
KRT14	3861
KRT16	3868
KRT6A	3853
KRT6B	3854
KRT6C	286887
KRT78	196374
LAD1	3898
LAG3	3902
LAIR1	3903
LAIR2	3904
LAMA2	3908
LAMA4	3910
LAMC2	3918
LAP3	51056
LAPTM5	7805
LAT	27040
LAT2	7462
LATS1	9113
LATS2	26524
LAX1	54900
LCE3D	84648
LCK	3932
LCN10	414332
LCOR	84458
LCP1	3936
LCP2	3937
LDB2	9079
LEP	3952
LEPR	3953
LGALS2	3957
LGALS9	3965
LGI2	55203
LGMN	5641

LHFPL6	10186
LHFPL2	10184
LIF	3976
LIFR	3977
LIG3	3980
LILRA1	11024
LILRA2	11027
LILRA3	11026
LILRA4	23547
LILRA5	353514
LILRA6	79168
LILRB1	10859
LILRB2	10288
LILRB3	11025
LILRB4	11006
LILRB5	10990
LILRP2	79166
LIMD2	80774
LIME1	54923
LIMS1	3987
LIPA	3988
LMOD1	25802
LMTK2	22853
UNQ6494	100129066
FAM83A-AS1	100131726
LINC00426	100188949
PCED1B-AS1	100233209
TNFRSF14-A:	115110
LINC00654	149837
LINC00926	283663
SMIM1	388588
LGALS17A	400696
MIR31HG	554202
LOC606724	606724
LOC653786	653786
LOC730101	730101
BMS1P20	96610
LOXL2	4017
LOXL3	84695
LPAR4	2846
LPL	4023
PLPPR4	9890
LPXN	9404
LRCH2	57631
LRMP	4033
LRP6	4040
LRRC15	131578
LRRC17	10234
LRRC25	126364
LRRC32	2615
NRROS	375387

LSAMP	4045
LSP1	4046
LST1	7940
LTA	4049
LTB	4050
LTBP2	4053
LTBR	4055
LTC4S	4056
LUM	4060
LY86	9450
LY9	4063
LY96	23643
LYL1	4066
LYN	4067
LYPD3	27076
LYPD5	284348
LYVE1	10894
LYZ	4069
MAGEL2	54551
MAN1A1	4121
MAN1A2	10905
MAN1C1	57134
MAOB	4129
MAP1LC3C	440738
MAP3K2	10746
MAP4K1	11184
MAP7D1	55700
43160	55016
43167	220972
MARCO	8685
MBNL3	55796
SLC25A53	401612
MCOLN2	255231
MED13	9969
MED13L	23389
MEF2B	100271849
MEF2C	4208
MEI1	150365
MEOX2	4223
MET	4233
MFAP3	4238
MFAP4	4239
MFNG	4242
MFRP	83552
MFSD7	84179
MGAT4A	11320
MGAT5	4249
MZB1	51237
MGP	4256
NA	4276
MICAL2	9645

MICB	4277
MIR155HG	114614
MITF	4286
MLPH	79083
MMP12	4321
MMP1	4312
MMP14	4323
MMP16	4325
MMP2	4313
MMP25	64386
MMP3	4314
MMP9	4318
MMRN1	22915
MMRN2	79812
MNDA	4332
MPEG1	219972
MPL	4352
MPP1	4354
MR1	3140
MRC1	4360
MRGPRF	116535
MRO	83876
MRPL27	51264
MRPL55	128308
MRPS12	6183
MRPS21	54460
MRVI1	10335
MS4A14	84689
MS4A1	931
MS4A2	2206
MS4A4A	51338
MS4A6A	64231
MS4A7	58475
MSR1	4481
MSRB3	253827
MVP	9961
MX1	4599
MXD1	4084
MXRA8	54587
MYCT1	80177
MYEF2	50804
MYO1F	4542
MYO1G	64005
MYO7A	4647
MYO9A	4649
N4BP2	55728
N4BP2L1	90634
NAALADL1	10004
NAIP	4671
NAP1L3	4675
NAPSB	256236

NBEA	26960
NBEAL1	65065
NCCRP1	342897
NCF1	653361
NCF1B	654816
NCF1C	654817
NCF2	4688
NCF4	4689
NCKAP1L	3071
NCOA2	10499
NCR1	9437
NCR3	259197
NEGR1	257194
NFAM1	150372
NFATC2	4773
NFKB2	4791
NFKBID	84807
NFKBIE	4794
NFYA	4800
NFYB	4801
NFYC	4802
NHLRC2	374354
NHSL2	340527
NID2	22795
NIPAL4	348938
NKG7	4818
NLRC3	197358
NLRC4	58484
NLRC5	84166
NLRP12	91662
NLRP3	114548
NMI	9111
NOD2	64127
NOTCH4	4855
NOVA2	4858
NOX4	50507
NR1H3	10062
NR5A2	2494
NRP1	8829
NRXN3	9369
NT5E	4907
NTM	50863
NTNG2	84628
NTRK1	4914
OAS1	4938
OAS2	4939
OAS3	4940
OASL	8638
TENM3	55714
OGFRL1	79627
OGN	4969

OLFML1	283298
OLFML2B	25903
OLFML3	56944
OLR1	4973
OMD	4958
OSCAR	126014
OSM	5008
OSMR	9180
OTOA	146183
OVOL1	5017
P2RX1	5023
P2RX4	5025
P2RX5	5026
P2RX7	5027
P2RY10	27334
P2RY11	5032
P2RY12	64805
P2RY13	53829
P2RY14	9934
P2RY8	286530
P4HA3	283208
PABPC5	140886
PADI2	11240
PAFAH1B2	5049
PAG1	55824
PAK5	57144
PALM2-AKAI	445815
PARM1	25849
PARP12	64761
PARP14	54625
PARP15	165631
PARP9	83666
PARVG	64098
PATL2	197135
PATZ1	23598
PBX4	80714
PCDH12	51294
PCDH17	27253
PCDH18	54510
PCDHGA12	26025
PCOLCE	5118
PCYOX1L	78991
PDCD1	5133
PDCD1LG2	80380
PDE1A	5136
PDE1B	5153
PDE3A	5139
PDE3B	5140
PDE4B	5142
PDE6G	5148
PDGFA	5154

PDGFB	5155
PDGFC	56034
PDGFRA	5156
PDGFRB	5159
PDGFRL	5157
PDIA2	64714
PDIA3	2923
PDZK1IP1	10158
PDZRN3	23024
PECAM1	5175
PEG3	5178
PFDN2	5202
PGLYRP4	57115
PGM5	5239
PHACTR1	221692
PIK3AP1	118788
PIK3CG	5294
PIK3R5	23533
PIK3R6	146850
PILRA	29992
PIM2	11040
PIP4K2A	5305
PKD2L1	9033
PKHD1L1	93035
PKIB	5570
PKP3	11187
PLA1A	51365
PLA2G2D	26279
PLA2G4E	123745
PLA2G7	7941
PLAC9	219348
PLCB2	5330
PLCB4	5332
PLCL1	5334
PLCL2	23228
PLD4	122618
PLEK2	26499
PLEK	5341
PLEKHM3	389072
PLEKHN1	84069
PLEKHO1	51177
PLEKHO2	80301
PLIN3	10226
PLVAP	83483
PLXDC1	57125
PLXNA4	91584
PLXNC1	10154
PLXND1	23129
PML	5371
PMP22	5376
PNLIPRP3	119548

PNMA2	10687
PNOC	5368
PODN	127435
POSTN	10631
POU2AF1	5450
POU2F2	5452
PLPP3	8613
PLPP4	196051
PLPP7	84814
PPBP	5473
PPFIA2	8499
PPL	5493
PPM1H	57460
PPM1M	132160
PPP1R13L	10848
PPP1R16B	26051
PPP1R9A	55607
PRAM1	84106
PRELP	5549
PREX1	57580
PREX2	80243
PRF1	5551
PRG2	5553
PRKAR2A	5576
PRKAR2B	5577
PRKCB	5579
PRKCQ	5588
PRKG1	5592
PRL	5617
PRLR	5618
PROCR	10544
PROM1	8842
PRRX1	5396
PRSS27	83886
PRTG	283659
PSAP	5660
PSMB10	5699
PSMB8	5696
PSMB9	5698
PSMC1	5700
PSMC2	5701
PSMC3	5702
PSMC4	5704
PSMC5	5705
PSMC6	5706
PSMD10	5716
PSMD11	5717
PSMD13	5719
PSMD14	10213
PSMD1	5707
PSMD2	5708

PSMD3	5709
PSMD4	5710
PSMD5	5711
PSMD6	9861
PSMD7	5713
PSMD8	5714
PSME1	5720
PSME2	5721
PSME3	10197
PSTPIP1	9051
PTAFR	5724
PTCRA	171558
PTGDR	5729
PTGDS	5730
PTGER2	5732
PTGFR	5737
PTGIR	5739
PTGIS	5740
PTH1R	5745
PTPN22	26191
PTPN6	5777
PTPN7	5778
PTPRB	5787
PTPRC	5788
PTPRCAP	5790
PTPRJ	5795
PTPRM	5797
PTPRO	5800
CAVIN1	284119
PUS10	150962
PVR	5817
PVRIG	79037
NECTIN2	5819
NECTIN3	25945
PYHIN1	149628
QPRT	23475
RAB20	55647
RAB33A	9363
RAB37	326624
RAB39A	54734
RAB39B	116442
RAB42	115273
RAB8B	51762
RAD23B	5887
RAD54L2	23132
RAET1E	135250
RAET1G	353091
RAET1L	154064
RAI2	10742
RAMP3	10268
RAPGEF2	9693

RAPGEF6	51735
RARRES2	5919
RARRES3	5920
RASAL3	64926
RASGRF2	5924
RASGRP2	10235
RASGRP3	25780
RASGRP4	115727
RASL12	51285
RASSF2	9770
RASSF3	283349
RASSF4	83937
RASSF5	83593
RASSF6	166824
RBM38	55544
RBP5	83758
RC3H2	54542
RCAN2	10231
RCN3	57333
RCSD1	92241
RDH12	145226
RECK	8434
REL	5966
RELB	5971
RELN	5649
RENBP	5973
REST	5978
RFTN1	23180
RFX5	5993
RFXANK	8625
RFXAP	5994
RGL1	23179
RGL4	266747
RGPD1	400966
RGS13	6003
RGS1	5996
RGS18	64407
RGS5	8490
RHOD	29984
RHOH	399
RHOJ	57381
RIF1	55183
RIMKLA	284716
RIN1	9610
RIN3	79890
CARMIL2	146206
RNASE1	6035
RNASE2	6036
RNASE6	6039
RNASE7	84659
RNF125	54941

RNF166	115992
RNF180	285671
RNF222	643904
LAMTOR2	28956
ROBO4	54538
ROCK2	9475
ROR1	4919
RPS6KA4	8986
RRN3P2	653390
RSAD2	91543
RTKN2	219790
RTN1	6252
RTP4	64108
RUFY4	285180
NA	84127
RUNX1T1	862
RUNX3	864
S100A12	6283
S100A16	140576
S100A2	6273
S100A7	6278
S100A7A	338324
S100A8	6279
S100A9	6280
S100B	6285
S1PR1	1901
S1PR4	8698
SALL2	6297
SAMD14	201191
SAMD3	154075
SAMD9	54809
SAMD9L	219285
SAMHD1	25939
SAMSN1	64092
SARDH	1757
SASH3	54440
SBNO1	55206
SBSN	374897
SCARF1	8578
SCARF2	91179
SCEL	8796
SCML4	256380
SCN7A	6332
SCUBE3	222663
SDC2	6383
SDCBP2	27111
SDR9C7	121214
SDS	10993
SDSL	113675
SEC24A	10802
SEC24D	9871

SECISBP2L	9728
SECTM1	6398
SELENBP1	8991
SELL	6402
SELP	6403
SELPLG	6404
43344	1731
43349	23157
SERINC5	256987
SERPINA1	5265
SERPINE1	5054
SERPINF1	5176
SERPING1	710
SFMBT2	57713
SFN	2810
SFRP2	6423
SFTPB	6439
SGCD	6444
SGIP1	84251
POMK	84197
PEAK1	79834
SH2B3	10019
SH2D1A	4068
SH2D2A	9047
SH2D3C	10044
SH2D5	400745
SHE	126669
SEM1	7979
SIGLEC10	89790
SIGLEC11	114132
SIGLEC12	89858
SIGLEC14	100049587
SIGLEC1	6614
SIGLEC5	8778
SIGLEC6	946
SIGLEC7	27036
SIGLEC8	27181
SIGLEC9	27180
SIGLEC17P	284367
SIRPB1	10326
SIRPB2	284759
SIRPG	55423
SIT1	27240
SKAP1	8631
SLA2	84174
SLA	6503
SLAMF1	6504
SLAMF6	114836
SLAMF7	57823
SLAMF8	56833
SLC10A2	6555

SLC11A1	6556
SLC12A3	6559
SLC15A3	51296
SLC17A9	63910
SLC18A2	6571
SLC1A7	6512
SLC24A4	123041
SLC25A45	283130
SLC29A3	55315
SLC2A5	6518
SLC34A2	10568
SLC39A2	29986
SLC45A3	85414
SLC6A12	6539
SLC7A7	9056
SLC8A1	6546
SLCO2B1	11309
SLCO5A1	81796
NA	342615
SLIT2	9353
SLIT3	6586
SLURP1	57152
SMAP2	64744
SMPDL3B	27293
SNAI3	333929
SNED1	25992
SNRPF	6636
SNTB1	6641
SNX20	124460
SOD3	6649
SON	6651
SOX17	64321
SOX5	6660
SP100	6672
SP110	3431
SP140	11262
SPAG4	6676
SPARC	6678
SPARCL1	8404
SPATA13	221178
SPI1	6688
SPIB	6689
SPN	6693
SPNS3	201305
SPOCK2	9806
SPON1	10418
SPRR1A	6698
SPRR1B	6699
SPRR2D	6703
SPRR2E	6704
SPRR2G	6706

SPRY1	10252
SRGN	5552
SSC5D	284297
SSTR3	6753
ST3GAL2	6483
ST3GAL5	8869
ST3GAL6	10402
ST6GAL1	6480
ST6GALNAC3	256435
ST8SIA4	7903
STAB1	23166
STAC3	246329
STAP1	26228
STARD13	90627
STARD8	9754
STAT1	6772
STAT4	6775
STAT5A	6776
STK17B	9262
STK33	65975
STRN	6801
STXBP6	29091
SUCNR1	56670
SULF1	23213
SULT1C2	6819
SULT1C4	27233
SULT2B1	6820
SUSD3	203328
SVOPL	136306
SYNE1	23345
SYT11	23208
SYTL3	94120
TAGAP	117289
TAOK1	57551
TAP1	6890
TAP2	6891
TAPBP	6892
TAPBPL	55080
TARP	445347
TBC1D10C	374403
TBCEL	219899
TBX21	30009
TBXA2R	6915
TBXAS1	6916
TCEAL7	56849
TCIRG1	10312
TCL1A	8115
TCN2	6948
TEK	7010
TESC	54997
TFEC	22797

TGFB1	7040
TGFB2	7042
TGFB3	7043
TGFBI	7045
TGFBR1	7046
TGFBR2	7048
TGFBRAP1	9392
TGM1	7051
TGM2	7052
THBS1	7057
THBS2	7058
THEMIS	387357
THPO	7066
THSD7A	221981
THY1	7070
TIE1	7075
TIFAB	497189
TIGIT	201633
TIMD4	91937
PAM16	51025
TIMM50	92609
TIMP2	7077
TIMP3	7078
TLR10	81793
TLR1	7096
TLR4	7099
TLR5	7100
TLR7	51284
TLR8	51311
TLR9	54106
TM4SF18	116441
TM6SF1	53346
DCSTAMP	81501
TMC8	147138
TMEM106A	113277
TMEM119	338773
TMEM140	55281
IGFLR1	79713
TMEM150B	284417
TMEM156	80008
TMEM170B	100113407
TMEM176A	55365
TMEM176B	28959
TMEM200A	114801
TMEM204	79652
TMEM229B	161145
TMEM233	387890
TMEM26	219623
TMEM47	83604
TMEM79	84283
SYNDIG1	79953

TMIGD2	126259
TNF	7124
TNFAIP6	7130
TNFAIP8L2	79626
TNFRSF10A	8797
TNFRSF10B	8795
TNFRSF10C	8794
TNFRSF10D	8793
TNFRSF11A	8792
TNFRSF11B	4982
TNFRSF12A	51330
TNFRSF13B	23495
TNFRSF13C	115650
TNFRSF14	8764
TNFRSF17	608
TNFRSF18	8784
TNFRSF1A	7132
TNFRSF1B	7133
TNFRSF25	8718
TNFRSF4	7293
TNFRSF6B	8771
TNFRSF8	943
TNFRSF9	3604
TNFSF10	8743
TNFSF11	8600
TNFSF12	8742
TNFSF12-TN	407977
TNFSF13	8741
TNFSF13B	10673
TNFSF14	8740
TNFSF15	9966
TNFSF18	8995
TNFSF4	7292
TNFSF8	944
TNFSF9	8744
TNIK	23043
TNIP3	79931
TNN	63923
TNNT2	7139
TNS3	64759
TOX	9760
TPK1	27010
TPSAB1	7177
TPSB2	64499
TPSD1	23430
TPSG1	25823
TRAF1	7185
TRAF3IP3	80342
TRANK1	9881
TRAT1	50852
TREM1	54210

TREM2	54209
TREML1	340205
TRIM21	6737
TRIM22	10346
TRIM61	391712
TRPC4AP	26133
TRPV2	51393
TRPV3	162514
TSHR	7253
TSHZ3	57616
TSLP	85480
TSPAN11	441631
TSPAN32	10077
TSPAN4	7106
TTBK2	146057
TTC16	158248
TTC21B	79809
TTC24	164118
TTC37	9652
TUBA4A	7277
TUBB6	84617
TXK	7294
NME8	51314
TYMP	1890
TYROBP	7305
UBA7	7318
UBASH3A	53347
UBD	10537
UBE2L6	9246
UBR1	197131
UBXN11	91544
UBXN1	51035
UCP2	7351
UHMK1	127933
ULBP1	80329
ULBP2	80328
ULBP3	79465
UNC13D	201294
UNC5C	8633
UNC93B1	81622
USHBP1	83878
USP12	219333
USP51	158880
UTS2	10911
VAMP5	10791
VASH1	22846
VAV1	7409
VCAM1	7412
VCAN	1462
VEGFA	7422
VEGFB	7423

VEGFC	7424
VENTX	27287
VGLL3	389136
VIM	7431
VMO1	284013
VNN2	8875
VPREB3	29802
VPS37D	155382
VSIG4	11326
VWF	7450
WARS	7453
WAS	7454
WDFY4	57705
WFDC12	128488
WIPF1	7456
WISP1	8840
WNT2	7472
XAF1	54739
XBP1	7494
XCL1	6375
XCL2	6846
XCR1	2829
XKR8	55113
XPNPEP2	7512
ZAP70	7535
ZBP1	81030
ZBTB10	65986
ZBTB32	27033
ZC3H12D	340152
ZCCHC24	219654
ZDHHC20	253832
ZEB1	6935
ZEB2	9839
ZFPM2	23414
ZKSCAN1	7586
ZMYND15	84225
ZNF185	7739
ZKSCAN8	7745
ZNF215	7762
ZNF366	167465
ZNF423	23090
ZNF469	84627
ZNF521	25925
ZNF620	253639
ZNF660	285349
ZNF671	79891
ZNF683	257101
ZNF804A	91752
ZNF80	7634
ZNF827	152485
ZNF831	128611

ZNF835	90485
LRP1	4035
EIF2A	83939
EIF2AK3	9451
EIF2AK2	5610
EIF2AK4	440275
EIF2AK1	27102
HMGB1	3146
ANXA1	301
PANX1	24145
P2RY2	5029
IFNA1	3439
IFNA2	3440
TLR3	7098
IFNAR1	3454
IFNAR2	3455
VTCN1	79679
HHLA2	11148
