# Supplementary material for: The Immune Subtypes and Landscape of Advanced-Stage Ovarian Cancer
Source: Vaccines (Basel). 2022 Sep 2;10(9):1451. doi: 10.3390/vaccines10091451 (PMC9501495; doi:10.3390/vaccines10091451)
Supplement: Supplementary file 1 [file vaccines-10-01451-s001.zip › Supplementary Materials File/Supplementary Materials File S2.pdf]

Supplementary Table 1. Primers for qRT-PCR analysis.

**Gene**	**Primer**	**Sequence (5' to 3')**
GAPDH	Forward	CAGCCTCAAGATCATCAGCA
	Reverse	TGTGGTCATGAGTCCTTCCA
CD2	Forward	CAGCCTGAGTGCAAAATTCA
	Reverse	CTCTGTGGGCTCTTGTCTCC
CD3D	Forward	GCAATACCAGCATCACATGG
	Reverse	GTCTCATGTCCAGCAAAGCA
CD3E	Forward	TGAGGGCAAGAGTGTGTGAG
	Reverse	TAGTCTGGGTTGGGAACAGG
