# Supplementary material for: The Immune Subtypes and Landscape of Advanced-Stage Ovarian Cancer
Source: Vaccines (Basel). 2022 Sep 2;10(9):1451. doi: 10.3390/vaccines10091451 (PMC9501495; doi:10.3390/vaccines10091451)
Supplement: Supplementary file 1 [file vaccines-10-01451-s001.zip › Supplementary Materials File/Supplementary Materials File S3.pdf]

Tag	IS2	IS3	IS1
UBE4B	2	0	2
ZMYM4	2	1	2
MACF1	7	5	3
UBL4B	3	1	0
ITGA10	3	3	2
ADAR	3	1	2
SIPA1L2	4	2	1
ZNF669	4	0	0
NBAS	4	2	0
LRP2	9	4	4
NCKAP1	2	1	2
ANKMY1	2	0	2
MST1R	6	2	4
CACNA1D	4	0	4
PIK3CA	5	3	0
TLR6	2	0	2
UGT2A1	2	3	0
SH3PXD2B	2	1	2
THSD7A	4	1	0
PCLO	9	6	2
ZSCAN21	5	0	0
PRSS58	2	1	1
GCN1L1	3	3	2
PXN	2	2	1
NOP9	4	0	0
VRTN	4	0	0
DYNC1H1	11	2	3
MCTP2	3	1	0
ZP2	2	1	1
TP53	104	72	43
CTC1	3	0	1
CNTNAP1	1	1	4
PPM1E	2	1	1
MUC16	13	9	7
ARHGAP35	2	3	3
YTHDF1	4	1	1
PPM1F	2	1	1
SMC1A	4	4	0
OCRL	1	2	1
SAGE1	2	0	2
LCT	5	3	5
TTN	39	26	25
COL4A3	2	1	1
FLNB	8	6	0
TRIM42	1	2	1
EIF5A2	3	1	0
WDFY3	5	6	6
HIPK2	4	3	1
KMT2C	5	7	6
ANK1	2	4	4

CNTRL	1	3	2
KAT6B	4	3	0
SACS	5	5	2
STON2	1	3	2
FUK	1	4	2
CAMKK1	2	1	1
NF1	8	4	3
SLC1A6	2	2	0
RPN2	4	1	0
CACNA1F	2	2	1
ADGRL2	1	2	1
OBSCN	5	5	1
RYR2	8	9	5
EIF2AK3	4	2	1
BMPR2	0	4	0
PIKFYVE	6	1	3
SETD2	0	4	1
ROBO1	5	4	1
ITK	1	4	3
EPHA7	3	2	2
CNTNAP2	3	4	1
MPDZ	2	3	1
PALM2-AKAI	0	4	1
CDH23	5	2	0
CYP2C19	1	1	2
SLK	2	2	1
KMT2D	4	5	0
MED13L	3	1	2
CENPJ	4	1	1
NBEA	2	2	1
SYNE2	9	4	5
TRIP11	2	2	0
ZNF222	0	4	0
PHKA2	1	2	1
NRK	1	2	1
CCNL2	1	2	1
S1PR1	1	1	2
MAGI3	2	4	2
ADAM30	1	3	0
FLG2	12	7	4
ATP8B2	3	1	0
FLAD1	3	1	0
GON4L	2	3	1
URB2	5	2	1
TRIM67	1	3	0
SPRTN	2	1	1
LYST	2	4	0
PLD5	1	3	0
USP34	2	3	4
ATG9A	3	1	4
COL7A1	9	2	1

STAB1	2	2	2
CASR	2	4	0
SEMA5B	0	3	2
EPHB1	3	2	1
PPM1L	1	3	0
FNDC3B	3	1	1
EIF2B5	7	3	2
SLIT2	3	2	0
HERC3	2	2	1
ADAMTS12	2	2	0
DMXL1	3	1	0
KDM3B	3	2	3
PCDHB5	2	3	0
HIST1H1C	3	1	1
ZKSCAN4	0	3	2
TINAG	0	2	2
MDN1	6	8	5
THBS2	2	2	1
SDK1	2	4	2
TNRC18	2	2	3
NOD1	3	2	0
OGDH	0	2	2
ZAN	3	3	1
MGAM	2	2	2
RAB11FIP1	3	2	1
RGS22	2	3	1
KCNV1	1	3	1
DENND3	3	3	0
VLDLR	0	3	1
BNC2	2	2	0
KIF24	3	3	1
PIGO	4	2	1
FRMPD1	2	4	2
PRUNE2	10	6	6
GNA14	0	1	3
SVEP1	3	4	3
TNC	3	3	2
DNM1	1	1	2
SETX	2	2	1
LARP4B	1	2	1
SVIL	0	5	3
MGEA5	1	2	1
DHX32	3	2	1
KCNA4	3	2	0
KMT2A	6	4	4
CD163L1	2	2	3
HECTD4	5	2	1
DHX37	2	3	2
TSC22D1	2	2	0
F10	2	1	2
RPGRIP1	2	4	2

SYNE3	4	2	0
MGA	7	7	2
MAPKBP1	4	2	0
PLA2G4F	1	2	1
CLK3	1	2	1
UNC45A	4	1	0
IL4R	0	3	2
DRC7	3	1	1
DNAH2	4	4	2
CHD3	0	2	2
KIAA0100	2	3	2
EPB41L3	2	2	1
TRAPPC8	1	3	1
ZNF426	3	3	0
MYO9B	2	1	3
HPN	2	1	1
PROKR2	1	1	2
PLCB1	5	1	0
NCOA3	2	3	1
TMPRSS15	1	2	1
GAB4	1	2	1
PI4KA	2	2	1
THOC5	0	3	2
CPT1B	0	3	1
PGAM4	1	2	2
TBX22	3	2	3
POU3F4	2	2	1
IRS4	3	4	1
PAK3	1	2	1
NAA10	1	1	3
TKTL1	2	2	0
F8	3	3	2
KIF1B	6	0	0
DPYD	3	1	1
PEAR1	5	0	0
TBC1D8	2	2	1
SLITRK3	4	1	0
ZBTB49	3	1	0
PPEF2	3	1	0
MMRN1	4	0	2
MTHFD1L	1	2	1
CNGA4	1	3	0
SHANK2	4	1	0
ARID4A	6	1	1
CILP	2	1	1
CREBBP	6	1	3
TOP2A	8	5	1
TBCD	4	1	1
NOTCH3	3	1	1
CYP4F11	3	1	0
NCOA6	3	3	0

CTCFL	3	2	0
TBC1D8B	1	3	0
LUZP4	4	0	0
CENPF	3	0	2
OR2T1	3	0	1
COL3A1	4	0	2
SPHKAP	3	6	3
IL17RB	3	0	1
FAM193A	2	1	3
FRAS1	6	3	2
RAPGEF2	1	0	3
CMYA5	3	2	4
VCAN	4	5	4
PCDHA13	1	1	2
DNAH8	4	1	2
ZNF318	9	2	3
PKHD1	3	6	4
HGF	2	1	1
SAMD9	5	1	3
TRRAP	8	3	5
SRPK2	1	1	2
CSMD3	13	12	8
ITIH2	2	1	3
PLCE1	1	1	3
ABCC2	2	0	3
PPFIA1	3	0	2
NUMA1	3	1	3
ARID2	2	2	2
AKAP11	1	1	2
ATP7B	1	2	1
SMEK1	1	2	1
WDR20	3	1	1
IGHM	2	1	1
CASC5	2	1	1
EPB42	1	1	2
SLX4	6	3	1
ZKSCAN2	2	1	3
CHD9	4	5	1
ITGA2B	1	2	1
MALT1	2	1	1
PLA2G4C	1	0	3
CHD6	7	0	4
PCNT	3	5	1
USP9X	4	1	1
HTATSF1	2	3	3
CLSTN1	2	1	1
ADGRB2	4	4	1
GRIK3	1	1	2
HECTD3	2	0	3
NASP	1	2	1
ATP1A4	3	0	2

ZNF281	2	0	2
PCNXL2	2	1	4
KIF26B	4	3	4
NLRP3	4	1	2
APOB	5	9	5
ALK	1	1	3
BIRC6	6	1	4
FSHR	2	2	3
MOGS	3	0	3
POLR1A	3	2	1
CNTNAP5	6	4	2
MYO3B	3	0	3
HECW2	2	1	3
ABCA12	4	1	3
RPL32	1	1	2
KCNH8	2	1	2
SLC6A20	0	0	4
ITIH1	4	1	1
ARHGAP31	4	2	4
PARP14	1	4	4
PLCH1	2	4	4
MUC4	0	3	1
UGT2B4	2	0	2
NPFFR2	1	2	2
NAA11	1	2	1
FAT4	7	3	1
NAA15	1	0	3
SNX25	1	1	2
EXOC3	0	3	1
CTNND2	3	1	2
PRDM9	2	2	3
SPEF2	3	1	1
C5orf42	3	1	1
ADAMTS6	1	0	4
BDP1	3	0	2
HARS	4	0	1
PCDHB11	4	1	1
PCDHGA10	2	1	1
PCDHGB7	2	2	1
FAT2	3	4	3
IRF4	2	1	2
HIVEP1	3	2	3
HLA-E	1	1	2
TRERF1	1	1	2
TDRD6	1	3	3
MCM3	1	1	2
KLHL31	3	1	1
COL19A1	3	1	2
CD109	1	2	1
TNFAIP3	1	2	2
PLEKHG1	2	2	6

ARID1B	7	4	2
HDAC9	2	0	2
DNAH11	6	4	4
STAG3	1	3	1
RELN	5	5	3
AASS	2	0	3
FLNC	4	2	2
TNPO3	1	2	2
AGK	1	1	2
KCNU1	2	1	2
PRKDC	4	2	3
TRPA1	3	0	1
TG	4	4	5
TOPORS	1	1	2
ZBTB5	2	2	3
DAPK1	4	0	1
PTCH1	2	4	2
ANGPTL2	2	1	1
GARNL3	4	0	3
BEND7	2	1	2
GAD2	2	2	1
PCDH15	5	3	1
TET1	3	4	4
ZSWIM8	2	3	2
MKI67	7	3	6
MUC5B	1	1	3
PIK3C2A	2	1	1
QSER1	1	1	4
F2	3	0	2
AGBL2	0	2	2
CCDC87	1	1	2
FAT3	11	8	5
MMP8	2	2	1
SIK3	2	1	3
NOP2	2	1	1
A2ML1	3	0	3
CAPRIN2	2	1	1
ABCD2	3	0	1
ADAMTS20	1	1	2
BIN2	2	0	2
CS	1	1	2
STAT6	2	2	1
MGAT4C	2	0	3
FGD6	0	2	2
NOS1	2	0	2
SBNO1	0	1	3
DNAH10	8	3	5
AACS	2	2	2
STARD13	0	1	3
PNN	1	1	2
FANCM	3	1	1

PYGL	1	0	3
ZFYVE26	4	2	3
DICER1	1	2	2
NPAP1	3	0	3
TTBK2	4	1	3
CA12	2	1	1
SLCO3A1	1	1	2
PPL	1	0	3
ABCC1	6	1	2
SMG1	4	3	2
AC004381.6	3	1	1
RBBP6	1	1	2
SF3B3	2	0	2
HSD17B2	1	2	1
DNAH9	5	3	3
STAT3	1	1	2
TEX14	2	1	2
TANC2	4	0	1
KIAA0195	6	2	3
RNF213	8	2	3
ZNF521	5	2	2
EPG5	2	0	2
INSR	3	1	3
ZNF791	4	0	1
CATSPERG	1	2	1
PLEKHG2	4	1	2
CYP2A13	1	2	1
PPP1R15A	0	0	4
SHANK1	1	0	3
LILRA5	1	1	3
PEG3	4	1	2
ZNF551	2	2	5
SLC9A8	3	0	2
NFATC2	2	1	2
SON	4	2	1
TRPM2	2	0	5
DIP2A	1	2	2
CCT8L2	4	1	2
LRRC75B	4	0	1
GAS2L1	0	2	2
TRIOBP	2	2	1
EP300	4	0	3
SMC1B	1	2	1
ZBED4	1	1	2
ZBED1	5	1	3
DCAF8L1	3	1	1
ITIH6	1	0	3
FRMPD3	1	2	3
RGAG1	1	1	2
KIAA1210	5	2	3
TENM1	4	7	3

AFF2	1	2	1
MAGEA1	1	2	1
PADI1	4	0	0
C1orf87	1	3	0
TTF2	3	0	1
ADAMTSL4	3	1	2
ASPM	3	4	1
CRB1	4	3	3
COL4A4	6	2	1
ANKHD1-EIF	3	3	1
UHRF1BP1	5	1	0
DST	5	3	3
HIP1	3	3	2
CACNA2D1	1	1	2
MUC17	5	5	4
DOCK4	5	2	0
FAM135B	5	4	0
SPATA31E1	3	2	0
GAPVD1	1	1	2
FRMPD2	2	2	0
PKD2L1	4	3	0
PYGM	3	0	1
USP15	3	1	0
TPP2	2	1	2
SPTB	3	4	1
RYR3	4	2	2
GPR139	2	2	0
PRPF8	7	2	1
SUPT5H	4	2	1
PREX1	5	1	0
SCN7A	3	1	2
DGKD	5	0	0
TRANK1	5	3	3
PTPRD	2	1	2
KRAS	2	0	2
RNF31	5	0	0
UNC79	4	3	2
DMXL2	5	2	0
MYH11	11	4	2
CDK12	6	6	3
MYOM1	2	3	1
DOPEY2	4	3	2
MTHFR	2	0	2
FHL3	2	0	2
PTPRF	3	1	1
MBD5	4	0	0
PCDH7	3	1	1
PKD2	3	0	2
PCDHGB5	3	1	0
HIST1H2AE	1	3	0
BRD2	2	2	0

ST18	4	2	1
ZNF462	3	3	2
KIF18A	3	0	1
NRDE2	4	0	0
RASGRF1	1	2	1
MYO18A	3	2	1
SMARCA4	3	1	1
KIAA1683	3	2	1
ZNF208	1	1	2
PAF1	3	0	1
DYRK1B	2	2	0
TMPRSS2	2	1	2
FLNA	2	2	0
CPT2	1	1	2
USH2A	15	2	3
XIRP2	8	2	0
FAM124B	4	0	0
ITPR1	5	0	0
RREB1	3	1	0
SAMD9L	3	2	3
ZFHX4	6	5	1
UBR5	5	3	2
GABBR2	4	0	1
MADD	4	2	1
HSPG2	8	4	2
LRP1B	12	4	3
MED12L	5	2	0
TRMT44	3	1	0
MAP3K1	2	1	2
MYH1	3	5	2
FARSA	3	1	1
ACTRT2	1	2	1
CGN	6	0	1
DYSF	2	2	1
DPP10	2	2	0
WDR33	3	1	1
ZNF804A	2	3	0
INPP5D	4	1	2
CSRNP1	3	0	2
DNAH1	8	2	2
FRMD4B	4	0	0
GOLGB1	4	4	1
PAPD7	2	2	1
HECW1	6	2	0
WDR91	4	1	1
ADAM7	3	1	0
ZNF16	3	0	2
ACTN3	3	0	1
MON2	2	2	2
NAV3	5	0	2
KCNH5	1	0	3

SETBP1	3	3	1
LTN1	2	2	0
SYNJ1	2	3	0
FAM47C	4	0	1
HUWE1	4	5	3
LAS1L	3	2	0
CAMTA1	3	4	0
MFN2	5	1	2
CROCC	3	1	1
KIF17	2	2	1
NCF2	3	3	1
FMN2	2	1	2
TRAPPC12	3	2	0
FAM171B	1	3	0
FANCD2	4	1	0
IQSEC1	1	3	1
DOCK3	4	1	2
ENAM	1	3	0
TULP1	0	3	1
COL27A1	2	1	1
AKNA	2	2	1
NUP160	0	3	2
WARS	2	1	1
AOC2	3	3	4
DSG4	1	2	1
MYO1F	4	1	0
MAN2B1	4	2	1
CACNA1A	2	3	1
EPS15L1	3	1	2
PLCB4	1	2	1
ZNFX1	9	3	0
PABPC5	3	2	1
L1TD1	3	1	0
POGZ	6	0	0
LAMC2	2	1	1
ADCY3	5	3	1
HAT1	2	1	1
FN1	3	1	0
PHLDB2	3	3	0
KLB	1	0	3
PCDHGC5	3	1	1
BTN1A1	4	1	1
HOXA2	3	1	2
DLGAP2	3	1	1
KANK1	2	2	3
ITGA8	4	0	0
DNM1L	2	3	0
BRCA2	7	1	3
LPIN2	5	0	0
FBXO27	2	1	1
EYA2	3	0	1

ZNF217	2	0	3
CFAP47	2	2	0
ECE1	4	1	2
ANKRD35	0	3	1
INSRR	2	2	1
DNAH7	0	5	3
FGA	1	1	2
PCDHB7	2	2	0
C5	4	3	0
PLEKHA7	4	1	0
KIAA1551	0	3	1
LRP1	6	4	3
DNAH3	6	5	7
ZNF516	1	2	1
MTOR	3	4	6
IGSF21	2	2	0
EIF4G3	1	3	1
ZSCAN20	3	3	1
RAD54L	1	1	2
PI4KB	3	1	1
FLG	11	7	7
TNN	2	4	0
KIF21B	2	2	2
AGBL5	0	2	2
RANBP2	3	2	1
SCN3A	3	5	1
TTC21B	1	3	1
DOCK10	0	5	0
MYRIP	1	1	2
LTF	3	1	0
HPS3	2	2	1
GPR149	5	4	0
CCDC39	3	1	1
HTT	4	2	2
JAKMIP1	2	2	0
KIAA1109	3	3	4
ZNF827	2	1	1
DCHS2	1	4	0
TAS2R1	2	1	1
ENC1	4	3	1
APC	4	3	3
PCDHB8	2	2	0
PDE6A	1	2	1
NSD1	1	2	1
PTCHD4	3	1	2
COL9A1	2	1	1
SYNE1	9	8	3
PHF14	2	1	1
ABCB4	3	4	0
COL1A2	3	1	0
OPN1SW	2	3	0

PLXNA4	0	5	2
CSMD1	3	8	0
NCOA2	1	2	3
DCAF4L2	1	1	2
DDX58	1	3	0
CUBN	5	3	1
SH3PXD2A	1	2	1
SORCS3	0	2	2
SLCO1C1	2	3	0
SCN8A	6	4	0
ACACB	4	3	3
TMEM132B	0	4	1
RB1	5	3	0
PCK2	0	3	1
AXIN1	1	2	2
SRRM2	2	2	2
ZSCAN32	4	1	0
GRIN2A	3	5	0
CDH8	3	2	1
CDH16	3	1	1
CEP192	2	2	1
COL5A3	0	7	3
FKBP8	2	1	1
TSHZ3	4	8	1
TRPM4	2	1	1
KLK7	1	2	1
SIGLEC10	3	3	2
TMC2	3	3	1
HEPH	1	2	1
NLGN3	2	2	0
BRWD3	2	2	1
SLITRK4	1	4	0
HIVEP3	2	5	1
INTS3	2	2	1
NUP210L	1	3	1
BRINP3	2	3	0
ITPKB	2	2	0
PRKCD	1	3	0
ADGRG7	3	3	0
DNAH5	8	8	2
CDH10	4	2	1
FBXL21	1	2	1
PCDHB10	1	2	3
EPHA1	1	2	1
DLG5	2	5	2
IGSF22	4	3	0
MYCBP2	4	3	2
USP7	1	3	0
ZNF492	1	3	0
ZNF91	2	1	2
MXRA5	3	4	2

TAF1	3	2	0
AQP10	3	1	0
ASH1L	4	0	1
FAM129A	4	2	1
FOSL2	3	1	1
RIF1	2	2	2
ZDBF2	4	2	0
UGT1A9	3	1	0
USP19	4	1	2
COL6A6	3	2	4
SYNGAP1	1	3	1
ZNF479	2	3	0
LAMB1	4	2	2
AOC1	3	1	0
BMS1	5	0	1
PALD1	1	3	1
SORL1	6	1	0
CNTN1	2	2	2
CYFIP1	2	2	0
UNC13C	5	0	2
LINS	3	1	2
ZNF646	3	3	1
LAMA3	4	4	1
NOL4	4	1	2
PKN1	2	1	1
NCAN	1	2	2
ATP1A3	2	1	1
DHX34	1	1	2
MKL1	3	0	1
ATRX	5	4	3
PTPRZ1	5	4	3
PAXIP1	3	1	0
RIMS2	3	1	2
GRIN2B	3	0	1
DGKH	3	2	1
WWP2	2	1	1
AGL	3	1	1
HMCN1	6	6	5
TMEM214	4	0	2
GPR75	1	0	3
GIGYF2	0	2	4
CENPE	1	1	2
PCDHGB1	2	2	2
TNXB	2	5	1
PREX2	4	3	1
DOLK	2	1	1
TMEM132D	2	2	3
SLITRK1	4	2	1
NIN	1	1	2
NID2	5	1	2
EML2	3	0	1

LAIR1	2	1	1
KIF16B	3	0	2
RALGAPA2	3	2	2
ZBTB21	2	0	2
COL4A6	2	1	3
CLCN6	2	2	1
PADI3	1	3	1
MYOM3	4	1	1
CSMD2	4	3	1
KDM4A	3	1	2
GJA8	3	2	2
SELENBP1	1	2	2
GPR161	2	0	2
PLXNA2	4	1	1
GREB1	2	2	1
HEATR5B	4	1	0
MAP4K3	2	2	1
SLC20A1	2	0	2
NEB	7	4	1
KLHL23	3	1	0
COL5A2	4	0	0
ANKZF1	2	1	2
PTPRN	2	1	2
SLC22A14	2	2	0
TRAK1	2	1	1
ZNF445	3	1	3
MAP4	3	3	1
ERC2	3	2	0
CCDC37	3	0	1
IGSF10	3	1	2
ETV5	4	0	1
DMP1	3	1	0
FAT1	8	1	1
PDZD2	6	3	1
AGXT2	3	2	0
NUP155	2	2	0
AFF4	4	1	1
JADE2	2	1	1
TENM2	5	4	1
GFPT2	3	2	0
ITPR3	6	7	2
SLC26A8	3	2	1
USP49	4	2	0
UBR2	2	0	2
CUL7	4	0	0
DOPEY1	2	1	2
HOXA1	2	0	2
ABCA13	5	5	1
COBL	2	2	0
CUX1	1	3	1
CHRNB3	2	1	1

CHD7	6	1	5
CNBD1	3	1	0
TRPS1	7	2	1
TRAPPC9	2	1	1
FAM120A	1	2	1
PTPN3	1	4	0
SPTAN1	3	3	2
NUP188	5	1	2
GPR107	3	0	1
RAPGEF1	4	1	2
EHMT1	4	2	0
ARHGAP21	3	1	0
RET	3	2	1
ADAMTS14	3	2	0
PIK3AP1	5	2	1
BTRC	2	0	2
RAG1	2	1	2
KBTBD4	3	1	1
AHNAK	6	4	3
JRKL	1	3	0
PCSK7	1	0	3
CACNA1C	7	1	0
LTBR	2	1	2
ERBB3	2	3	1
PRDM4	1	2	1
TPCN1	2	3	1
KNTC1	2	2	1
EP400	5	3	2
ATP11A	6	0	2
RP11-468E2.	2	1	1
NYNRIN	3	1	1
MLH3	4	1	0
PLCB2	3	1	2
BAHD1	2	2	0
MAP1A	8	1	1
TMC3	3	1	0
ZNF710	2	0	2
MAN2A2	2	2	3
TSC2	1	3	2
CNOT1	4	0	1
MYO1C	2	2	0
ITGAE	3	1	3
KLHL10	2	2	0
STRN4	3	1	0
ZNF772	3	0	1
ZNF135	3	0	1
TGM3	3	2	1
HSPA12B	3	1	0
SLX4IP	3	0	2
JAG1	3	1	1
ASXL1	4	1	0

ZHX3	5	3	0
ARFGEF2	3	2	1
PTGIS	2	1	3
ATP9A	3	1	1
CBLN4	3	1	0
NCAM2	4	1	0
GRIK1	4	0	0
BCOR	3	2	0
PODN	3	2	1
DAB1	2	1	1
ADCY10	3	0	2
PPP1R12B	2	3	0
NCKAP5	3	3	3
CCDC80	3	1	3
EVC2	4	1	2
SLC6A3	3	1	0
ZBED9	3	2	0
TIAM2	2	2	2
ZNF107	3	0	1
CYP3A43	3	1	0
DOCK5	3	5	0
LRP4	2	2	0
OR5M11	2	1	1
FLT3	2	1	1
FLT1	2	3	0
ZNF106	3	1	2
SLC6A2	4	1	0
LRRC36	2	2	0
ACTG1	2	1	1
SCAF1	2	0	2
NLRP4	3	2	0
MC3R	3	1	0
PDE4DIP	1	3	0
PAPPA2	2	3	0
PSME4	0	4	0
POTEF	0	3	1
ITGA4	1	2	1
ABCB6	4	2	1
PASK	2	3	1
USP4	1	1	2
ITIH4	1	3	1
ROBO2	1	3	1
NIPBL	1	4	1
FBXO38	3	2	2
DOCK2	3	2	2
PDE10A	1	3	0
TBRG4	3	1	0
OR2AE1	2	1	1
VPS13B	3	4	2
CERCAM	2	2	2
MRC1	1	2	2

KIAA1217	4	2	1
NAV2	0	2	2
PDZRN4	2	2	0
UHRF1BP1L	2	2	0
PARP4	2	1	3
LRRK1	0	3	1
SUPT6H	1	2	1
C3	0	3	1
ZNF560	2	2	1
ADGRE3	3	4	0
DSCAM	1	3	2
MAP3K15	0	2	2
DMD	5	4	0
CCNB3	4	2	0
NPHP4	1	1	2
CASZ1	1	2	1
NBPF10	3	1	0
ARHGEF11	2	3	2
KCNH1	0	3	2
MAP3K19	3	1	3
SCN10A	4	3	0
DCBLD2	0	3	1
PPP2R3A	1	1	2
SORBS2	2	3	0
LPA	3	4	1
RP1	3	2	1
E2F5	3	1	0
COL14A1	0	4	1
GANAB	2	3	0
GALNT6	2	2	1
CCNA1	2	1	1
RTN1	2	3	0
AHNAK2	9	4	4
HERC2	5	1	0
DPP8	2	2	0
LCTL	1	3	0
BFAR	2	2	0
PER1	3	2	1
MYH4	5	8	2
NAGLU	4	1	3
ADAM11	1	2	1
CDC27	1	1	2
TTLL6	1	2	2
DNAH17	5	5	3
NPHS1	1	2	1
RYR1	10	3	3
ZNF343	1	2	1
SEZ6L	3	2	1
DLG3	2	2	1
PCDH19	2	3	0
ACTRT1	4	1	0

LRRC7	3	3	2
ACTN2	2	2	0
OR2G6	1	6	0
SDC1	1	1	3
OTOF	4	2	0
DCTN1	3	2	1
FAP	1	3	0
NEK10	1	2	2
MME	1	2	1
ANK2	2	4	2
SULF1	2	3	2
FKBP15	1	3	0
NCAM1	3	2	1
EPS8	1	3	0
KRT4	1	3	1
GOLGA3	2	3	0
TUBA3C	1	2	1
NALCN	8	2	1
SRCAP	4	3	3
MYH2	0	4	2
ATAD5	0	3	1
ACLY	2	2	0
RIMS4	3	4	0
USP26	2	2	4
KIF2C	3	1	1
CAD	4	2	3
PREPL	3	1	1
ALMS1	5	3	4
SLC5A7	2	2	1
PDE11A	4	3	1
SLC6A6	2	2	0
SIDT1	4	2	0
STXBP5L	4	1	2
PEX5L	2	2	0
FBN2	5	2	1
PPARGC1B	3	1	0
FAM120B	3	0	1
CAMSAP1	2	3	3
DOCK1	4	0	2
INCENP	3	0	2
NCAPD2	3	1	1
RIMBP2	2	2	0
TEP1	2	1	3
ARHGEF40	3	0	2
PAPLN	2	0	2
IFT140	4	0	1
GPR179	2	1	2
ITGB4	1	1	3
CCDC40	2	2	0
RELB	3	1	3
NLRP5	3	1	0

SIGLEC1	3	1	1
MCM3AP	8	2	3
L3MBTL2	3	2	1
F5	4	3	0
QARS	2	2	0
TENM3	3	2	1
ALPK3	3	1	2
IGHV3OR16-	2	2	1
TLDC1	2	1	3
MED13	3	0	1
ASXL3	4	4	3
NWD1	1	3	0
OVGP1	1	2	1
PLB1	2	3	1
FBXO11	2	1	1
ADAMTS9	3	2	3
MYLK	4	3	2
EDIL3	2	1	1
PCDHB2	3	2	0
CASP8AP2	6	1	1
NEUROD6	3	0	1
NUP205	2	3	3
ZFPM2	3	3	0
PKHD1L1	6	3	3
DSCAML1	4	1	2
LRRIQ1	3	0	1
AKAP13	5	4	3
ABCA3	3	1	0
C16orf62	3	2	0
PALB2	5	0	1
CCDC116	2	0	2
KDM5C	5	1	1
DOCK11	6	1	1
DDR2	1	2	1
PRRC2C	4	0	3
KCNH7	1	1	2
XRN1	2	1	2
PPARGC1A	3	0	1
FASTKD3	3	0	1
TFAP2D	2	1	2
POT1	1	2	1
GRM8	2	0	3
C11orf30	2	1	2
ATP2B1	2	0	2
DLG4	3	1	2
ZNF101	3	0	2
TPTE	1	1	2
NPR1	0	3	1
BBX	2	0	2
BOD1L1	1	1	2
ADAMTS19	2	2	1

COL15A1	3	1	1
MYO3A	4	1	3
KIAA1549L	1	2	3
PCDH9	3	3	1
ATXN2L	1	2	1
TARBP1	1	3	0
GPR55	3	1	2
TTLL3	1	3	0
GPRIN3	2	3	1
BRD9	2	2	0
MROH2B	2	2	0
ABCB5	2	1	3
BAZ1B	2	2	0
DENND2A	2	1	1
UBE3C	3	3	0
FBXO18	3	3	0
ANKRD30A	1	5	0
MYOF	1	3	1
NRAP	4	2	0
TACC2	3	2	0
SIDT2	0	4	3
ALG10B	1	1	2
PKD1L2	3	1	0
UNC13A	4	3	0
CLTCL1	5	2	1
AIM1L	4	0	1
TNPO2	4	0	1
ESF1	1	1	2
CHEK2	2	0	2
ARHGAP29	4	1	0
PTPN14	4	0	2
IRS1	5	1	1
HDLBP	2	0	2
BRCA1	2	6	2
MYH9	5	0	2
CACHD1	3	1	1
KMT2E	1	2	2
ZNF777	2	1	1
DDIAS	2	0	2
EXPH5	0	1	3
ELMSAN1	2	0	4
DNMT1	4	1	4
ZNF441	1	2	2
ATP13A1	2	1	2
DNAJC28	1	1	2
KCNS3	0	2	2
MYH15	2	2	4
DTX3L	1	4	1
SI	7	3	3
ADAM29	3	0	3
ADGRF5	0	2	3

GRIK2	3	0	1
LAMA2	4	2	3
PTPRN2	1	2	3
SECISBP2	0	2	2
MUSK	1	1	2
ANK3	2	2	4
DNA2	0	1	3
MARK2	2	0	2
PEAK1	1	4	1
MYCBPAP	0	0	4
PTCHD1	2	1	1
CXCR4	3	0	1
PAX3	2	1	2
N4BP2	2	2	2
MYO10	1	0	3
GRM1	1	1	2
KIAA1549	3	1	1
EXTL3	2	1	1
EYA1	1	0	3
TAF1L	3	4	2
CNTN5	2	2	1
COL4A2	3	1	2
RORA	0	2	2
MOV10L1	2	2	2
GRID1	1	3	0
TRPM1	4	0	1
DOCK7	2	1	1
CEP350	6	2	2
AHCTF1	1	3	0
SCN1A	5	2	2
SLC12A8	2	1	1
SORCS2	5	1	0
CPZ	3	2	0
POLR2B	4	0	1
LRBA	2	1	2
CDH6	4	0	1
CHD1	2	1	1
PCDHAC1	5	1	1
RIMS1	5	0	1
BACH2	4	0	1
LIMK1	4	0	0
AKAP9	4	0	1
HAS2	2	1	1
PSAP	2	2	1
C10orf12	1	2	1
MYO7A	3	1	1
POLE	2	2	1
COL4A1	7	2	1
CHD8	5	0	3
MOK	4	0	0
SYNM	3	1	3

NCOR1	6	1	1
GPATCH8	5	0	1
ADGRE2	2	2	0
MYBPC2	3	1	1
FRMPD4	3	2	1
ADGRG4	3	1	1
SETD5	2	4	0
POLQ	0	2	2
TACR3	3	1	1
ARFGEF3	2	2	0
ENPP2	1	3	1
DYNC2H1	0	3	1
VWF	3	4	1
PAN2	1	3	0
RTF1	2	2	0
VPS13C	0	4	3
ANKRD27	3	3	0
GPATCH1	2	2	0
NLRP11	3	1	0
PHACTR3	1	2	1
DGKK	4	1	1
FAAH2	1	2	1
CELSR2	1	4	2
CACNA1E	1	3	0
CACNA1S	3	2	5
GLI2	3	2	2
RAD54L2	2	2	1
WDR55	1	2	2
PCDHA10	1	2	1
ASCC3	2	2	0
UST	2	1	1
KIAA0196	2	1	1
THBS1	0	3	1
CLN6	1	1	2
ERN2	2	4	0
FANCA	2	3	0
MYH8	4	2	0
RP11-385D1	1	2	1
ATP9B	3	2	1
FCGBP	7	6	3
DGCR8	1	2	1
ZCCHC12	3	2	1
PTCHD2	2	2	1
TMCO4	3	2	1
HRNR	6	2	1
TNR	0	3	1
LAMC1	2	1	2
NPAS2	3	1	0
FIGN	1	3	0
TATDN2	2	3	1
CCR5	3	1	1

CPOX	3	1	0
PARP9	0	3	1
TRIO	2	3	2
PRRC2A	0	4	1
VCPIP1	1	2	1
NR4A1	3	1	0
CDK17	0	3	1
LMO7	1	2	2
CDH11	0	2	2
HYDIN	3	3	1
SMCR8	2	2	0
LENG8	4	1	0
SALL4	1	5	1
ITSN1	1	3	1
HTR1D	2	1	1
GOLGA4	3	3	0
BSN	3	3	0
KIF21A	0	4	1
IL21R	2	2	1
GPR142	0	3	1
SRP68	2	2	1
ZNF236	5	4	1
ZNF536	5	2	1
ZNF528	2	2	1
ZNF835	2	1	4
SZT2	4	4	0
SRSF11	2	2	0
MYOC	3	2	1
GPR155	0	3	1
ING5	2	2	0
NOTCH4	4	2	1
ADGRB3	1	2	1
PDE1C	0	3	1
TRIM56	2	2	0
CYP11B1	1	4	2
PARP10	1	2	2
CCBL1	2	2	1
C10orf71	0	3	2
AGAP6	2	2	1
PDE6C	2	3	0
SOX5	3	2	0
TBC1D4	3	1	1
MYH7	1	1	3
P4HB	2	2	1
LAMA1	5	4	1
ZNF226	2	2	0
NLRP13	4	1	0
RNPEP	3	3	0
KIDINS220	4	0	2
SOS1	2	2	0
ANKRD36	3	1	0

UBA7	4	0	0
PKD1L1	4	2	0
WNK1	2	1	2
PROCA1	4	0	0
ZNF28	3	1	0
CTPS2	3	0	1
IQGAP3	2	1	2
HOXD10	0	2	2
ZNF608	1	3	2
DLC1	0	3	2
TEK	1	1	2
FBN1	1	1	2
EMC1	3	2	0
WNT2B	1	2	1
CLCN1	2	1	1
FAM171A1	2	5	0
NEBL	1	2	1
NELL1	2	2	0
ME3	3	2	0
ROBO3	1	1	3
KSR2	1	2	1
CHD2	2	2	0
PDILT	0	3	1
USP31	2	1	1
ZNF564	1	3	0
GPR50	0	3	2
PLXNA3	1	2	1
MFSD2A	2	1	2
ZNF644	2	1	2
SERPINC1	1	0	3
MAT2A	0	2	2
C3orf30	2	0	2
COL6A5	1	1	2
EGFLAM	2	1	3
MAP1B	3	1	1
VARS2	3	1	2
FHL5	2	1	1
LMTK2	3	1	1
PIK3CG	4	0	2
SGK223	1	3	3
MROH5	1	3	1
PCSK5	2	1	1
TBC1D2	2	1	2
INVS	1	1	3
ASTN2	0	2	2
ABL1	3	2	2
ZNF365	2	0	2
JMJD1C	3	4	3
FAM35A	0	2	2
RRP12	1	2	3
ATRNL1	3	0	2

GRIA4	1	1	3
KCNJ1	1	1	2
PTPRB	1	3	4
MPHOSPH8	1	1	2
SLC7A1	1	1	2
FREM2	2	5	3
ACIN1	1	1	2
FBXO34	3	0	1
BDKRB1	1	1	2
WDR25	3	0	1
MYO5C	1	1	2
MYO5A	2	2	4
NEDD4	2	1	1
ADCY9	2	2	2
MKL2	1	1	2
TNRC6A	1	2	3
ZFHX3	8	3	1
ARHGEF15	0	2	2
ABCA10	3	0	3
FHOD3	2	1	2
CPAMD8	4	2	1
ITPKC	2	2	1
NLRP2	2	1	3
ZNF543	2	3	3
ZNF671	0	0	4
MYO18B	6	4	1
FAM47B	2	1	1
HTR2C	3	2	1
RNF113A	3	0	2
BCORL1	3	2	2
IGSF1	2	3	3
NES	0	2	2
PTPN7	3	0	1
CCDC108	1	3	1
TNIK	0	2	2
IQGAP2	2	1	3
VPS52	2	1	1
KIFC1	3	2	1
IGF2R	3	1	2
CD163	1	1	2
TAF15	0	2	2
HLF	3	0	1
NPC1	2	1	1
MAGEC2	1	1	3
KIAA1324L	3	0	1
SMURF1	4	2	0
PXDNL	3	0	1
CACNA1B	2	0	2
PCDH20	3	3	4
EFCAB6	5	1	1
PRDM16	4	1	1

UBR4	6	4	1
KIAA1324	2	1	2
CR1	3	1	1
HK2	3	1	0
CPS1	4	0	0
TGFBR2	2	1	1
CLASP2	3	0	1
MCF2L2	2	1	1
TACC3	2	1	1
ADGRV1	4	3	4
TCOF1	4	1	0
CUL9	5	2	1
SLC29A1	2	0	2
TMEM8B	2	2	0
SKIDA1	3	0	1
SORCS1	4	1	0
ZBTB16	1	4	0
TECTA	7	3	3
ITPR2	3	2	0
LIG4	4	0	0
AP1G2	2	1	1
AKAP6	6	2	2
CAPN3	2	2	0
BNC1	4	2	1
TTC23	4	0	0
NUP93	3	0	1
SLC12A3	4	2	0
RAPGEFL1	2	1	1
CYP4F2	3	3	1
MAST3	4	0	2
NLRP12	3	0	2
CSE1L	1	3	0
TRAPPC10	3	1	2
EIF3L	1	1	2
L1CAM	1	3	0
SPTA1	5	5	1
SCN5A	5	1	1
ATP10B	1	2	1
EPHB6	2	1	2
KLHL38	3	1	2
ESPL1	4	1	3
ATP8A2	3	1	1
ATP2A1	1	1	2
JAK3	4	3	1
ZNF133	5	1	0
OPRD1	1	2	1
FPGT	2	3	0
ERICH3	3	1	2
EPS8L3	1	1	2
AHCYL1	2	2	0
KDM5B	0	2	2

AVPR1B	0	3	1
DISP1	4	1	1
TTC13	1	1	2
MYT1L	1	2	1
MGAT5	3	3	0
TANC1	1	2	2
MAP2	6	4	1
SCN11A	2	4	0
ZKSCAN7	1	2	1
ALDH1L1	1	3	1
ADGRL3	5	3	0
SLC4A4	1	3	0
SHROOM3	1	4	1
HTR1A	3	1	0
ADAM19	3	1	0
KIAA0319	0	3	2
XXbac-BPG1	1	2	1
ABCC10	3	1	0
PHF3	1	3	0
HIVEP2	7	1	1
AHR	1	3	0
TSPAN33	0	2	2
NCAPG2	1	2	1
DCSTAMP	2	1	1
LRP12	2	1	1
ACO1	2	1	2
CDK5RAP2	2	3	1
PRRC2B	2	1	2
COL5A1	2	3	0
ANKRD26	1	4	1
SLC29A2	2	2	1
SPTBN2	3	4	3
HEPHL1	3	3	0
CIT	1	1	2
RBM23	1	1	2
ZC2HC1C	1	2	1
ISM2	2	2	0
UBR7	1	3	0
OCA2	5	2	1
TLN2	3	3	3
MYO9A	1	1	2
IDH3A	1	2	1
NTRK3	4	1	3
CAMTA2	2	3	2
SPAG5	3	2	2
AOC3	3	1	0
ALPK2	2	4	0
PIK3R2	4	2	1
MYH14	1	3	0
ZNF160	1	3	0
CEP250	2	1	3

DLGAP4	3	1	0
SLC35E4	1	1	2
DDX17	1	1	2
USP51	2	1	1
PRPS1	2	1	2
ITSN2	2	3	0
PCDHGA11	3	1	1
PDCD11	2	3	0
CACNA1G	6	3	3
C19orf44	0	4	1
DIDO1	5	2	1
TRMU	2	2	0
FBN3	2	3	1
PRDM2	4	1	0
CDC20	3	1	0
AP4B1	2	2	0
CFH	4	2	0
C2orf78	1	1	2
ZEB2	0	3	1
FSIP2	1	1	2
ADAMTS16	1	3	0
FER1L6	2	6	0
VWA2	1	2	1
TRIM51	0	4	1
OSBP	2	2	1
STIP1	2	2	2
METTL21B	1	3	0
AGBL1	3	2	0
GNAL	1	4	0
MCOLN1	2	3	0
ZNF473	3	3	1
C20orf194	4	2	1
CD93	3	1	0
CASS4	3	1	2
ENOX2	2	1	1
EPHA2	3	1	0
SP100	2	1	1
LATS1	5	0	1
SUN1	3	0	1
IGHG4	4	0	0
CYP1A1	3	2	2
ZZEF1	4	1	0
ARHGAP36	2	1	1
VAV3	2	1	1
HEATR1	4	1	1
XIRP1	4	2	1
DZIP1L	2	0	2
MLLT4	3	1	0
AGTPBP1	3	1	0
TYR	2	3	0
KIAA2022	3	1	0

WDTC1	2	1	1
COL16A1	4	2	1
PYHIN1	3	0	1
ARHGAP30	2	0	2
PPFIA4	3	1	0
PTPRG	5	0	1
KALRN	2	3	1
PTPN13	4	0	1
SH3TC2	3	0	2
TAF6	4	1	1
MATN2	2	2	1
ABRA	2	1	1
C2CD3	5	0	0
TENM4	3	3	0
DLG2	3	0	1
PLXNC1	2	0	3
STAB2	2	1	1
HCN4	2	2	0
GAN	1	2	1
SIGLEC8	3	0	3
JADE3	4	0	0
AIM1	6	2	3
PPP1R3A	7	1	2
ZFAND1	2	2	1
ZNF503	1	2	1
VWA3A	3	1	1
RNF20	0	4	0
CCT2	2	1	2
TCEB3	4	2	0
HIPK1	2	3	1
CFAP45	1	3	0
ABL2	4	1	1
SPTBN1	4	3	2
CTNNA2	3	0	1
MYO7B	3	0	4
P2RY13	2	2	0
TFRC	2	1	1
FNDC9	3	1	0
GABRG2	2	1	1
SLC2A12	3	1	0
SCRIB	2	2	0
TLE4	2	0	2
FNBP1	2	1	1
SBF2	2	1	2
HIPK3	3	1	1
CKAP5	2	3	0
USP5	3	1	2
ZFC3H1	2	3	0
RASAL1	2	1	2
RNF17	3	0	1
GJD2	3	1	0

EIF2AK4	2	1	1
ERCC4	3	0	1
PTPRT	6	2	2
WDR45	3	1	0
ORC1	1	1	2
SCAMP3	1	3	0
TRIP12	2	2	0
COL6A3	2	7	0
ALB	2	2	1
ZNF425	2	3	1
RP1L1	4	2	1
ASAP1	2	1	3
ABCA1	2	2	1
DNTT	2	3	0
KBTBD7	1	2	1
COL1A1	3	1	0
SPEN	7	4	2
LRRC41	5	1	0
WDR6	3	0	1
KIAA2018	1	0	3
ATR	4	0	0
CABS1	3	1	0
LARP1	3	1	0
LAMB4	5	0	0
NRP1	2	2	0
ZCCHC8	3	0	1
IGF1R	4	0	0
ABCA9	5	2	0
MAGEB1	2	1	1
MAST2	3	1	2
IL12RB2	4	0	0
COL2A1	4	1	1
EWSR1	2	1	1
LRIG2	1	2	1
CA14	3	1	1
NKTR	2	1	1
REV3L	2	2	0
UTRN	4	2	1
MYOM2	1	2	2
APLP2	1	1	3
NT5DC3	1	2	1
CCDC63	1	3	0
MPHOSPH9	1	1	2
TMC8	0	1	3
MYO5B	2	2	1
THBS4	3	1	1
CDK13	1	2	1
KDM5A	3	1	1
DNAJC11	3	1	3
COL11A1	1	1	3
MOV10	2	2	1

SPAG17	1	1	3
DCST2	2	0	2
GTF3C2	1	1	2
STK36	2	0	2
SLC34A2	2	1	2
MDC1	1	2	2
WDR27	2	0	2
MCM7	1	2	1
COL22A1	4	1	2
VCP	1	2	2
ZNF518A	1	2	1
LZTS2	2	0	2
INPP5F	0	1	3
DMBT1	1	1	2
SSRP1	2	0	2
DDB1	2	1	1
CPT1A	1	3	3
VPS11	4	1	3
MYO1H	1	2	3
FRY	2	2	2
ZBTB25	0	2	2
C15orf59	2	0	2
MYH13	1	2	4
MYH3	4	2	1
SETD4	1	1	2
ASMT	3	0	1
ZXDA	2	1	2
MAMLD1	1	2	1
PRPS1L1	3	2	0
VPS13A	1	3	0
OTOGL	2	3	0
CLIP1	3	2	1
MYO16	2	3	2
KIAA1755	1	2	1
SCN9A	2	1	1
CDH17	0	3	3
ARHGEF10	3	1	1
CHAMP1	4	0	0
KCNQ2	2	2	1
SLC5A3	2	2	0
TXNRD2	4	0	0
FARP2	3	0	1
DROSHA	3	1	0
DSP	4	3	1
PTK7	2	2	1
SPATA31A6	2	2	0
AMBRA1	3	1	0
UBC	3	1	0
LRRTM4	2	2	1
ZSWIM2	3	1	0
RNF216	2	1	1

ZKSCAN1	2	1	1
TTC17	4	0	0
NEURL4	4	0	0
NFE2L1	3	1	1
FPGT-TNNI3	2	2	0
CFAP46	2	1	1
ERCC5	1	1	2
UBE2O	3	2	0
CTAGE1	1	2	1
FAM120C	1	5	0
MYCL	4	1	2
COL24A1	3	2	0
TMEM131	5	3	0
RUFY1	3	1	1
HACE1	3	1	0
COPS6	3	0	1
LRRK2	3	3	3
TICRR	3	2	2
FLCN	3	1	0
MIB1	1	1	2
MUM1	4	0	0
SLC44A2	3	1	0
ZNF665	4	0	0
ZNF512	4	0	0
SLC9A4	3	1	1
BCS1L	5	1	2
TMPPE	3	0	1
ABCC12	2	1	1
PTPRS	2	0	3
C5AR2	1	2	1
RPRD2	2	2	1
THBS3	2	2	0
GALNT13	1	2	1
ACVR2B	3	1	0
RB1CC1	3	4	0
ADCY8	0	2	2
TRPM3	2	3	1
WNK3	1	3	2
ABCA4	1	3	2
TPO	2	1	1
SLC8A1	0	2	2
SNRNP200	3	2	1
SLC11A1	3	0	1
TKT	1	1	2
ABCC5	0	2	2
PCDHA3	2	0	2
PDGFRB	1	1	2
HIST1H1A	2	0	2
VARS	1	3	2
ZNF12	3	1	2
MROH1	4	0	2

EPC1	2	4	1
DCHS1	3	2	1
FAM186B	2	1	1
NCOR2	1	2	2
MAB21L1	4	0	1
NLRC5	4	1	2
FAM134C	2	0	2
TOB1	2	1	2
ZNF816	1	2	2
PTPRH	1	0	3
ZFX	1	1	2
C2orf71	2	0	2
MAST4	1	1	2
IGSF3	3	0	1
IFT172	2	3	1
TET3	3	0	1
CNGA3	2	1	1
PKP4	2	2	1
NR1I2	0	2	2
ECE2	2	1	2
KDR	0	2	2
MAGI2	2	1	3
TRUB2	2	1	1
FAM208B	2	0	2
ARHGAP32	3	0	1
E2F7	0	2	2
CNGB1	1	1	2
DSG1	1	1	2
RTTN	1	2	1
ZC3H7B	1	1	2
CNKSR2	3	0	2
ARSJ	3	1	1
ERMARD	2	1	1
EGFR	4	0	3
KIAA1462	3	2	2
PZP	4	1	1
ATG2B	2	1	1
PCDHB9	3	2	0
TRPC5	4	0	0
KCNQ3	3	1	0
UTP20	3	4	1
CECR1	0	1	3
WWC3	1	1	2
GBP3	2	2	0
KCNA3	3	1	0
EFTUD1	3	1	1
ABCA6	4	0	0
ANO3	3	1	0
SKIV2L	2	1	1
TRIM13	2	2	1
DHX38	2	2	1

PKDREJ	3	1	3
CD101	3	2	1
KLHL40	2	0	2
CLVS1	3	1	0
SLCO1A2	4	1	0
MBD1	4	0	0
LOXL4	4	1	0
FAM155B	3	1	0
LRRC4C	2	1	1
CDC42BPB	2	1	2
C2orf42	0	3	1
KIF5A	2	3	1
MORC2	5	2	0
RLF	3	3	1
DENND4B	3	1	0
FSTL5	3	0	1
C7	3	1	2
C10orf2	2	1	2
TMC7	2	1	2
ABCC11	3	0	1
BPTF	2	2	0
CNBD2	2	2	0
SULF2	3	1	0
SCNN1G	2	0	2
C8B	2	1	1
EXOC4	2	1	1
DUS2	2	0	2
PHF20	1	2	1
OR7D4	3	1	0
PPP2R1A	3	1	0
SLC4A10	2	2	1
KCNQ5	3	1	0
ZFP64	2	1	1
PLOD1	2	2	0
ADCY2	4	1	2
ATG9B	2	2	0
KRT6B	0	3	1
AMBN	1	1	2
GFRA1	3	0	1
OAS2	2	1	2
ZMYM3	3	0	1
BCAR3	2	1	1
MEGF10	3	1	0
ZSCAN31	2	2	1
RETSAT	1	2	1
TCF20	4	2	0
DNMBP	2	2	0
NRXN1	1	1	3
COL17A1	2	1	1
ADAM12	2	3	1
AARS	3	2	1

INPP5K	2	0	2
NETO1	3	1	0
CIC	3	2	0
KHDRBS1	3	1	0
INADL	4	0	0
JAK1	3	0	1
ERBB4	2	0	2
SH3BP4	4	1	0
NISCH	3	1	1
NDST1	4	0	1
HKDC1	3	0	1
PHF21A	2	0	2
SDS	1	1	2
MTHFD1	1	3	0
ZNF407	4	1	1
TSHZ2	3	3	0
ATAD2	1	2	2
GLB1L3	2	1	1
BTK	2	2	1
MIA3	0	2	2
FGD5	3	2	2
FRYL	2	0	2
USP54	0	3	1
TRPC6	2	1	1
DIAPH2	1	2	1
HTR3A	3	2	0
PPRC1	3	0	1
CDH3	2	0	2
SYCP1	4	0	0
SLC45A1	2	0	2
CELA2A	2	2	2
COPA	2	0	2
C2orf16	1	1	2
FAM13A	2	1	1
ZNF366	3	0	1
ERAP1	2	1	1
ARID5B	2	1	1
TRPC4	3	0	3
PDIA3	3	0	1
ZNF224	2	2	1
CECR2	2	1	1
COL4A5	0	2	2
C3orf20	1	1	2
MAML3	3	1	2
SPG7	2	0	2
GAS2L2	2	1	1
HUNK	0	2	2
CFAP43	4	0	1
DNHD1	3	2	0
JMJD6	4	0	1
IL1R2	2	1	1

PDZRN3	3	1	3
PCDHB13	2	2	2
RINT1	2	2	1
SLC13A1	2	1	1
AKR1B15	2	1	1
SLC4A2	1	1	2
ARHGAP20	2	1	1
WNK4	4	0	0
IRF8	2	2	0
INPP5B	3	2	0
LRRIQ3	0	3	1
ZNF711	2	1	1
DCC	1	2	1
PARS2	1	2	1
ZGRF1	1	2	2
TUBGCP4	1	2	1
EPHB2	2	1	1
RASGEF1B	2	1	1
ADAMTSL3	2	2	1
CDH12	2	2	0
RBM28	1	2	1
MAML2	2	2	0
PLA1A	4	0	0
ECSIT	2	1	1
FZD7	3	1	1
ANGEL1	3	1	2
TSHZ1	4	1	0
ASCC2	1	1	2
DOCK8	4	1	1
ATM	4	0	2
BRWD1	3	1	0
RGAG4	2	1	1
FGR	3	1	0
UNC13B	4	0	0
EDRF1	3	2	0
MAP6	5	0	0
HOOK2	4	0	0
NPC1L1	1	2	1
ARID4B	1	3	0
USP29	3	1	0
PTCH2	2	1	1
CTIF	4	0	0
AKAP8L	4	0	0
CD99L2	1	2	1
MAPK8IP1	1	1	2
IGDCC4	2	1	1
MYL4	1	2	1
PROX1	2	2	1
TMEM63C	2	1	1
CILP2	2	1	1
INO80D	3	0	1

GLI3	2	1	1
ERCC6	4	1	0
KCNJ15	2	2	1
MAP3K14	2	1	1
ZCCHC6	3	0	1
SYT12	3	0	1
ZNF214	3	1	0
ZBP1	1	2	1
CTTNBP2	0	3	1
PLEC	3	0	1
LGR4	2	1	1
VPS13D	3	1	1
AGO3	2	1	1
SLC36A3	2	1	1
NCOA7	4	0	0
