# Supplementary material for: The Immune Subtypes and Landscape of Advanced-Stage Ovarian Cancer
Source: Vaccines (Basel). 2022 Sep 2;10(9):1451. doi: 10.3390/vaccines10091451 (PMC9501495; doi:10.3390/vaccines10091451)
Supplement: Supplementary file 1 [file vaccines-10-01451-s001.zip › Supplementary Materials File/Supplementary Materials File S4.pdf]

| Tag | IS1 | IS2 | IS3 | IS1.pvalue | IS2.pvalue | IS3.pvalue |
|---|---|---|---|---|---|---|
| ZNF669 | 0 | 4 | 0 | 1 | 0.04969476 | 1 |
| ZSCAN21 | 0 | 5 | 0 | 1 | 0.02319089 | 1 |
| NOP9 | 0 | 4 | 0 | 1 | 0.04969476 | 1 |
| VRTN | 0 | 4 | 0 | 1 | 0.04969476 | 1 |
| CNTNAP1 | 4 | 1 | 1 | 0.01719984 | 0.98031283 | 0.90372207 |
| TTN | 25 | 39 | 26 | 0.02423107 | 0.87270494 | 0.82375754 |
| BMPR2 | 0 | 0 | 4 | 1 | 1 | 0.00989992 |
| SETD2 | 1 | 0 | 4 | 0.68565433 | 1 | 0.0372897 |
| PALM2-AKAI | 1 | 0 | 4 | 0.68565433 | 1 | 0.0372897 |
| ZNF222 | 0 | 0 | 4 | 1 | 1 | 0.00989992 |
| COL7A1 | 1 | 9 | 2 | 0.94063267 | 0.04775759 | 0.93937204 |
| GNA14 | 3 | 0 | 1 | 0.02798844 | 1 | 0.78826387 |
| KIF1B | 0 | 6 | 0 | 1 | 0.01077066 | 1 |
| PEAR1 | 0 | 5 | 0 | 1 | 0.02319089 | 1 |
| LUZP4 | 0 | 4 | 0 | 1 | 0.04969476 | 1 |
| RAPGEF2 | 3 | 1 | 0 | 0.02798844 | 0.92596011 | 1 |
| PLA2G4C | 3 | 1 | 0 | 0.02798844 | 0.92596011 | 1 |
| PCNXL2 | 4 | 2 | 1 | 0.03403359 | 0.92274025 | 0.93527534 |
| SLC6A20 | 4 | 0 | 0 | 0.00159837 | 1 | 1 |
| NAA15 | 3 | 1 | 0 | 0.02798844 | 0.92596011 | 1 |
| ADAMTS6 | 4 | 1 | 0 | 0.00676642 | 0.96174606 | 1 |
| PLEKHG1 | 6 | 2 | 2 | 0.00607831 | 0.98574145 | 0.88408615 |
| QSER1 | 4 | 1 | 1 | 0.01719984 | 0.98031283 | 0.90372207 |
| SBNO1 | 3 | 0 | 1 | 0.02798844 | 1 | 0.78826387 |
| STARD13 | 3 | 0 | 1 | 0.02798844 | 1 | 0.78826387 |
| PYGL | 3 | 1 | 0 | 0.02798844 | 0.92596011 | 1 |
| PPL | 3 | 1 | 0 | 0.02798844 | 0.92596011 | 1 |
| PPP1R15A | 4 | 0 | 0 | 0.00159837 | 1 | 1 |
| SHANK1 | 3 | 1 | 0 | 0.02798844 | 0.92596011 | 1 |
| ZNF551 | 5 | 2 | 2 | 0.01954739 | 0.97458768 | 0.84176412 |
| TRPM2 | 5 | 2 | 0 | 0.00457453 | 0.92274025 | 1 |
| ITIH6 | 3 | 1 | 0 | 0.02798844 | 0.92596011 | 1 |
| PADI1 | 0 | 4 | 0 | 1 | 0.04969476 | 1 |
| DGKD | 0 | 5 | 0 | 1 | 0.02319089 | 1 |
| RNF31 | 0 | 5 | 0 | 1 | 0.02319089 | 1 |
| MBD5 | 0 | 4 | 0 | 1 | 0.04969476 | 1 |
| NRDE2 | 0 | 4 | 0 | 1 | 0.04969476 | 1 |
| USH2A | 3 | 15 | 2 | 0.82033461 | 0.00923052 | 0.99645945 |
| XIRP2 | 0 | 8 | 2 | 1 | 0.03638213 | 0.88408615 |
| FAM124B | 0 | 4 | 0 | 1 | 0.04969476 | 1 |
| ITPR1 | 0 | 5 | 0 | 1 | 0.02319089 | 1 |
| CGN | 1 | 6 | 0 | 0.80364034 | 0.04552642 | 1 |
| FRMD4B | 0 | 4 | 0 | 1 | 0.04969476 | 1 |
| KCNH5 | 3 | 1 | 0 | 0.02798844 | 0.92596011 | 1 |
| ZNFX1 | 0 | 9 | 3 | 1 | 0.04775759 | 0.79816612 |
| POGZ | 0 | 6 | 0 | 1 | 0.01077066 | 1 |
| KLB | 3 | 1 | 0 | 0.02798844 | 0.92596011 | 1 |
| ITGA8 | 0 | 4 | 0 | 1 | 0.04969476 | 1 |
| LPIN2 | 0 | 5 | 0 | 1 | 0.02319089 | 1 |
| MTOR | 6 | 3 | 4 | 0.02966465 | 0.98477121 | 0.64397576 |

| | | | | | | |
|---|---|---|---|---|---|---|
| DOCK10 | 0 | 0 | 5 | 1 | 1 | 0.00305248 |
| DCHS2 | 0 | 1 | 4 | 1 | 0.96174606 | 0.0372897 |
| PLXNA4 | 2 | 0 | 5 | 0.43807713 | 1 | 0.03570068 |
| CSMD1 | 0 | 3 | 8 | 1 | 0.95714204 | 0.00557193 |
| TMEM132B | 1 | 0 | 4 | 0.68565433 | 1 | 0.0372897 |
| COL5A3 | 3 | 0 | 7 | 0.3360708 | 1 | 0.0136293 |
| TSHZ3 | 1 | 4 | 8 | 0.95342736 | 0.93915507 | 0.02363918 |
| SLITRK4 | 0 | 1 | 4 | 1 | 0.96174606 | 0.0372897 |
| SORL1 | 0 | 6 | 1 | 1 | 0.04552642 | 0.93527534 |
| GPR75 | 3 | 1 | 0 | 0.02798844 | 0.92596011 | 1 |
| GIGYF2 | 4 | 0 | 2 | 0.01719984 | 1 | 0.62385829 |
| COL5A2 | 0 | 4 | 0 | 1 | 0.04969476 | 1 |
| FAT1 | 1 | 8 | 1 | 0.9038719 | 0.03638213 | 0.98057794 |
| CUL7 | 0 | 4 | 0 | 1 | 0.04969476 | 1 |
| PTPN3 | 0 | 1 | 4 | 1 | 0.96174606 | 0.0372897 |
| PCSK7 | 3 | 1 | 0 | 0.02798844 | 0.92596011 | 1 |
| CACNA1C | 0 | 7 | 1 | 1 | 0.02379796 | 0.95657713 |
| MAP1A | 1 | 8 | 1 | 0.9038719 | 0.03638213 | 0.98057794 |
| GRIK1 | 0 | 4 | 0 | 1 | 0.04969476 | 1 |
| PSME4 | 0 | 0 | 4 | 1 | 1 | 0.00989992 |
| COL14A1 | 1 | 0 | 4 | 0.68565433 | 1 | 0.0372897 |
| OR2G6 | 0 | 1 | 6 | 1 | 0.98990826 | 0.0048341 |
| ANKRD30A | 0 | 1 | 5 | 1 | 0.98031283 | 0.01365312 |
| EXPH5 | 3 | 0 | 1 | 0.02798844 | 1 | 0.78826387 |
| ELMSAN1 | 4 | 2 | 0 | 0.01719984 | 0.86891223 | 1 |
| DNA2 | 3 | 0 | 1 | 0.02798844 | 1 | 0.78826387 |
| MYCBPAP | 4 | 0 | 0 | 0.00159837 | 1 | 1 |
| MYO10 | 3 | 1 | 0 | 0.02798844 | 0.92596011 | 1 |
| EYA1 | 3 | 1 | 0 | 0.02798844 | 0.92596011 | 1 |
| LIMK1 | 0 | 4 | 0 | 1 | 0.04969476 | 1 |
| MOK | 0 | 4 | 0 | 1 | 0.04969476 | 1 |
| CACNA1S | 5 | 3 | 2 | 0.03301647 | 0.92997259 | 0.88408615 |
| PRRC2A | 1 | 0 | 4 | 0.68565433 | 1 | 0.0372897 |
| SALL4 | 1 | 1 | 5 | 0.80364034 | 0.98990826 | 0.03570068 |
| KIF21A | 1 | 0 | 4 | 0.68565433 | 1 | 0.0372897 |
| ZNF835 | 4 | 2 | 1 | 0.03403359 | 0.92274025 | 0.93527534 |
| UBA7 | 0 | 4 | 0 | 1 | 0.04969476 | 1 |
| PROCA1 | 0 | 4 | 0 | 1 | 0.04969476 | 1 |
| FAM171A1 | 0 | 2 | 5 | 1 | 0.92274025 | 0.03570068 |
| SERPINC1 | 3 | 1 | 0 | 0.02798844 | 0.92596011 | 1 |
| ZNF671 | 4 | 0 | 0 | 0.00159837 | 1 | 1 |
| CPS1 | 0 | 4 | 0 | 1 | 0.04969476 | 1 |
| ZBTB16 | 0 | 1 | 4 | 1 | 0.96174606 | 0.0372897 |
| LIG4 | 0 | 4 | 0 | 1 | 0.04969476 | 1 |
| TTC23 | 0 | 4 | 0 | 1 | 0.04969476 | 1 |
| C19orf44 | 1 | 0 | 4 | 0.68565433 | 1 | 0.0372897 |
| FER1L6 | 0 | 2 | 6 | 1 | 0.95533232 | 0.01434153 |
| TRIM51 | 1 | 0 | 4 | 0.68565433 | 1 | 0.0372897 |
| GNAL | 0 | 1 | 4 | 1 | 0.96174606 | 0.0372897 |
| IGHG4 | 0 | 4 | 0 | 1 | 0.04969476 | 1 |
| C2CD3 | 0 | 5 | 0 | 1 | 0.02319089 | 1 |

| | | | | | | |
|---|---|---|---|---|---|---|
| JADE3 | 0 | 4 | 0 | 1 | 0.04969476 | 1 |
| RNF20 | 0 | 0 | 4 | 1 | 1 | 0.00989992 |
| MYO7B | 4 | 3 | 0 | 0.03403359 | 0.73434218 | 1 |
| COL6A3 | 0 | 2 | 7 | 1 | 0.97458768 | 0.00553238 |
| KIAA2018 | 3 | 1 | 0 | 0.02798844 | 0.92596011 | 1 |
| ATR | 0 | 4 | 0 | 1 | 0.04969476 | 1 |
| LAMB4 | 0 | 5 | 0 | 1 | 0.02319089 | 1 |
| IGF1R | 0 | 4 | 0 | 1 | 0.04969476 | 1 |
| IL12RB2 | 0 | 4 | 0 | 1 | 0.04969476 | 1 |
| TMC8 | 3 | 0 | 1 | 0.02798844 | 1 | 0.78826387 |
| INPP5F | 3 | 0 | 1 | 0.02798844 | 1 | 0.78826387 |
| MYH13 | 4 | 1 | 2 | 0.03403359 | 0.98990826 | 0.71440246 |
| CHAMP1 | 0 | 4 | 0 | 1 | 0.04969476 | 1 |
| TXNRD2 | 0 | 4 | 0 | 1 | 0.04969476 | 1 |
| TTC17 | 0 | 4 | 0 | 1 | 0.04969476 | 1 |
| NEURL4 | 0 | 4 | 0 | 1 | 0.04969476 | 1 |
| FAM120C | 0 | 1 | 5 | 1 | 0.98031283 | 0.01365312 |
| MUM1 | 0 | 4 | 0 | 1 | 0.04969476 | 1 |
| ZNF665 | 0 | 4 | 0 | 1 | 0.04969476 | 1 |
| ZNF512 | 0 | 4 | 0 | 1 | 0.04969476 | 1 |
| PTPRH | 3 | 1 | 0 | 0.02798844 | 0.92596011 | 1 |
| TRPC5 | 0 | 4 | 0 | 1 | 0.04969476 | 1 |
| CECR1 | 3 | 0 | 1 | 0.02798844 | 1 | 0.78826387 |
| ABCA6 | 0 | 4 | 0 | 1 | 0.04969476 | 1 |
| MBD1 | 0 | 4 | 0 | 1 | 0.04969476 | 1 |
| INADL | 0 | 4 | 0 | 1 | 0.04969476 | 1 |
| SYCP1 | 0 | 4 | 0 | 1 | 0.04969476 | 1 |
| WNK4 | 0 | 4 | 0 | 1 | 0.04969476 | 1 |
| PLA1A | 0 | 4 | 0 | 1 | 0.04969476 | 1 |
| UNC13B | 0 | 4 | 0 | 1 | 0.04969476 | 1 |
| MAP6 | 0 | 5 | 0 | 1 | 0.02319089 | 1 |
| HOOK2 | 0 | 4 | 0 | 1 | 0.04969476 | 1 |
| CTIF | 0 | 4 | 0 | 1 | 0.04969476 | 1 |
| AKAP8L | 0 | 4 | 0 | 1 | 0.04969476 | 1 |
| NCOA7 | 0 | 4 | 0 | 1 | 0.04969476 | 1 |
