# Supplementary material for: The Immune Subtypes and Landscape of Advanced-Stage Ovarian Cancer
Source: Vaccines (Basel). 2022 Sep 2;10(9):1451. doi: 10.3390/vaccines10091451 (PMC9501495; doi:10.3390/vaccines10091451)
Supplement: Supplementary file 1 [file vaccines-10-01451-s001.zip › Supplementary Materials File/Supplementary Materials File S5.pdf]

Tag	green	turquoise	yellow	black	brown	blue
TCGA-04-13	0.00607834	-0.063829	-0.0614019	0.04757502	0.03914654	0.00364739
TCGA-04-13	-0.040053	-0.0310256	-0.0829003	0.15393931	0.08063647	-0.0348575
TCGA-04-13	-0.0780937	-0.1042595	-0.0126354	-0.0597557	-0.0529966	0.13950849
TCGA-04-15	-0.0181764	0.01468949	0.02461677	-0.0605702	0.05016675	-0.0512894
TCGA-04-15	-0.0713388	-0.101582	-0.0318285	0.016653	-0.0418331	0.12048949
TCGA-04-16	-0.0230416	0.00339027	0.01708007	-0.0409575	-0.0872591	0.03046756
TCGA-04-16	-0.0994311	-0.1122562	-0.0454115	-0.0127734	-0.0220875	0.10847949
TCGA-09-03	-0.1185836	-0.0409404	-0.0838403	0.19784824	0.07065794	0.00850145
TCGA-09-16	-0.0541812	-0.0484526	-0.0425994	0.06109146	0.00315683	0.06165167
TCGA-09-16	-0.040384	0.05248784	0.0277471	-0.0165545	-0.0121667	-0.0438436
TCGA-09-16	0.07736708	-0.0075971	0.02946222	-0.0014382	-0.0447368	0.05396078
TCGA-09-16	0.0901178	0.02914376	0.04076766	-0.0476781	-0.0692981	-0.0140179
TCGA-09-16	0.06106025	0.02806908	0.06826081	-0.0257953	-0.0046232	-0.0646411
TCGA-09-20	0.07751669	0.00524873	-0.0222641	-0.0274116	-0.0465482	0.00962793
TCGA-09-20	0.1187437	0.03337404	0.03044393	-0.0512442	-0.0519013	-0.0165339
TCGA-09-20	0.02593828	0.005265	0.04428423	-0.0527965	-0.019639	-0.008503
TCGA-09-20	-0.087163	-0.0480739	-0.0572112	0.12646382	0.06246345	-0.0506912
TCGA-10-09	-0.0217877	-0.076779	-0.0562016	0.04812566	-0.0355906	0.03958838
TCGA-10-09	0.06232269	-0.0108849	0.02537053	-0.0487959	-0.0767041	0.09178521
TCGA-10-09	0.08470003	-0.0924419	-0.0526023	0.05717796	0.01604951	0.07180643
TCGA-10-09	0.06695239	0.0025573	0.07212248	-0.0313816	-0.1041253	0.07624224
TCGA-10-09	0.05723543	-0.1179016	-0.0136384	-0.0125649	-0.0667475	0.13167183
TCGA-13-07	0.03838524	-0.0477007	-0.0005202	-0.0601175	-0.0595365	0.07595532
TCGA-13-07	0.00669202	-0.0226382	0.00228263	-0.0442619	-0.0102428	0.04867893
TCGA-13-07	-0.010805	0.0361742	-0.006073	0.01906627	-0.0017548	-0.0006621
TCGA-13-07	0.00578035	-0.0101158	0.01954504	-0.0890636	-0.0162179	0.05661992
TCGA-13-07	-0.0328624	0.01026651	-0.0436563	-0.0499497	-0.0271903	0.04280641
TCGA-13-07	-0.0033657	-0.0405706	-0.0714976	0.06999903	0.05721073	-0.0160184
TCGA-13-07	-0.0388441	-0.0668587	-0.0123049	-0.0637716	-0.0339339	0.09704141
TCGA-13-08	0.04423004	-0.1243015	-0.0661849	0.00968862	-0.029109	0.08841524
TCGA-13-08	-0.0878561	0.0222309	-0.0328209	0.01583134	0.09147212	-0.0825532
TCGA-13-08	0.04217033	0.04974399	0.0457255	0.02335185	0.03482793	-0.0358878
TCGA-13-08	0.03989735	-0.0483602	0.01310454	0.02427187	-0.0393827	0.09217741
TCGA-13-08	0.04503067	0.01890806	0.04913325	-0.0523476	0.00991069	0.02594498
TCGA-13-09	-2.03E-05	-0.0309667	-0.0257163	0.05062223	0.03808585	0.05105474
TCGA-13-09	0.06624569	-0.0433705	0.00896923	-0.0185418	-0.0420925	0.05303855
TCGA-13-09	0.05323593	-0.0557539	-0.0005412	-0.0677465	-0.0258556	0.02480078
TCGA-13-09	0.01482219	0.03160519	-0.0176555	0.02133868	0.07193328	-0.0713475
TCGA-13-09	-0.0126042	0.07435292	0.01458539	-0.0239807	0.01699482	0.00326924
TCGA-13-09	0.02425478	-0.078458	0.00171683	0.0320569	-0.0163217	0.05914107
TCGA-13-14	-0.0119434	-0.0034048	0.00273719	0.03816518	0.04111169	0.00237824
TCGA-13-14	-0.0419252	-0.1085133	-0.0302835	-0.0082866	-0.0378989	0.12558036
TCGA-13-14	-0.0412489	-0.1150114	-0.0558724	-0.0247639	-0.011194	0.09898884
TCGA-13-14	0.08333389	0.00232984	-0.0006981	0.02918483	0.01912577	-0.0213402
TCGA-13-14	-0.060836	0.11233074	0.04459348	-0.0007755	0.05995063	-0.0918841
TCGA-13-14	0.02394968	-0.0017084	-0.0097117	0.0258939	0.03890402	0.0100531
TCGA-13-14	-0.1025808	-0.0351311	-0.0814256	0.02291924	0.11904479	-0.0204033
TCGA-13-14	-0.0629745	-0.0678643	-0.046685	0.02739534	0.07412378	0.03079458
TCGA-13-14	-0.0189679	-0.0640385	-0.0394147	-0.0418363	-0.0714903	0.09300368
TCGA-13-14	0.0794103	-0.0572654	-0.0339314	-0.0240091	-0.0271746	0.0669757

TCGA-13-14	-0.0068872	-0.0396998	-0.0002908	-0.0652693	-0.0101213	0.04514299
TCGA-13-14	0.13701373	0.04477762	0.10714326	-0.0581428	-0.0969476	0.05468397
TCGA-13-14	0.05093724	-0.0150732	0.02049462	0.08480311	0.01377206	0.01882279
TCGA-13-15	-0.0717181	-0.0352048	-0.0228564	0.00306376	0.05432307	0.04698394
TCGA-13-15	-0.0317601	-0.0270434	-0.0075068	-0.0215388	0.07498529	-0.0482048
TCGA-13-15	0.05643556	-0.0393456	0.00480484	-0.0686917	-0.0671404	0.04617832
TCGA-13-15	0.07078713	0.04944726	0.09147811	-0.0420871	0.01189706	-0.0037963
TCGA-13-15	-0.0405732	-0.0041077	0.00590142	-0.0211048	0.04934932	0.00851503
TCGA-13-15	0.04359085	-0.0397526	0.02658363	0.00109281	-0.0379772	0.04059906
TCGA-13-15	0.03209638	-0.0290332	-0.0418688	0.02796645	0.03566248	-0.0005532
TCGA-13-15	-0.0094344	0.00036423	0.00867293	-0.0145788	0.02113836	0.01326118
TCGA-13-20	0.05868415	0.03711329	0.09263372	-0.0489808	-0.018013	0.00627216
TCGA-20-16	0.00026432	0.02849336	0.01220991	-0.0070695	0.00016924	-0.0008947
TCGA-23-10	0.039435	-0.0306585	-0.001494	-0.019673	-0.0428348	0.08365623
TCGA-23-10	-0.1097485	-0.0687508	-0.0359361	0.03407387	-0.032069	0.11508229
TCGA-23-10	0.02936346	0.01087796	0.00665098	0.16328042	0.02868896	-0.0313452
TCGA-23-10	-0.0016439	0.04815583	-0.0236744	-0.0415293	0.00934739	0.00124723
TCGA-23-10	0.03657063	-0.0375907	0.06704946	-0.0390669	-0.0559361	0.0641235
TCGA-23-10	0.11098153	-0.0375261	-0.067016	0.0105365	-0.0103955	-0.0115065
TCGA-23-11	-0.08336	-0.1138807	-0.070698	0.09189578	-0.0141275	0.08603681
TCGA-23-11	0.02588407	-0.0803366	-0.0589336	0.0134766	-0.0051358	0.0412481
TCGA-23-11	-0.0838697	-0.0948944	-0.1055834	0.0747378	0.05251189	-0.0044319
TCGA-23-11	-0.0089807	0.07047416	-0.0093368	0.01055111	0.05927197	-0.0385178
TCGA-23-11	0.03806478	0.0150703	0.01234027	-0.0089754	-0.0210464	0.00891917
TCGA-23-11	-0.0274227	0.02284079	0.04394286	0.02104105	-0.018184	-0.005739
TCGA-23-11	0.08548902	0.0335006	0.05231623	-0.0151555	-0.0157149	-0.0359497
TCGA-23-11	0.10024196	0.07523391	0.09427876	-0.0293247	-0.0870986	-0.0279454
TCGA-23-20	0.00829913	0.0350206	-0.0216134	-0.020499	-0.0170345	-0.0281537
TCGA-23-20	0.07963779	0.03647177	-0.0242498	0.01397461	0.0125317	-0.0206647
TCGA-24-09	0.05116536	0.00095621	0.03084188	-0.0036433	-0.0879002	0.09362659
TCGA-24-09	-0.0596755	-0.0213164	-0.0589543	0.01154996	0.06862624	0.02431836
TCGA-24-09	0.03437891	-0.0571254	-0.0047974	0.02135448	-0.0370709	0.04846096
TCGA-24-09	0.0105332	-0.0687069	-0.0196006	0.03454905	0.02740952	0.02503989
TCGA-24-11	-0.0084432	0.04425614	0.06681617	-0.0222915	-0.0330782	0.00313649
TCGA-24-11	-0.0452173	-0.0228705	0.01581994	-0.0348539	0.01490394	6.25E-05
TCGA-24-14	0.12399723	-0.0154183	-0.0258286	0.01399792	-0.0608186	0.01321543
TCGA-24-14	0.02323558	0.00165179	-0.0218903	-0.0637795	-0.0646244	0.02895278
TCGA-24-14	0.0908008	0.06460859	0.064589	-0.0017029	0.01280511	-0.0904734
TCGA-24-14	-0.0256493	-0.0255621	-0.0219907	0.01386244	0.0667167	-0.0367208
TCGA-24-14	0.03181303	-0.0791897	-0.0535707	0.0424456	-0.0273108	0.07019202
TCGA-24-14	0.05410298	-0.0398383	-0.0149918	0.05093259	-0.0153978	-0.0066615
TCGA-24-14	0.03165687	-0.0209528	-0.0603064	0.09510599	0.03733021	-0.048225
TCGA-24-14	-0.0061063	-0.0200798	-0.0655795	-0.0219902	-0.0301621	0.04563487
TCGA-24-14	-0.1056249	-0.1028782	-0.0759107	0.07505387	0.02376587	0.03754078
TCGA-24-14	-0.0301092	0.03390359	0.00545897	-0.0512584	-0.0343402	0.05259115
TCGA-24-14	-0.0675649	0.06804656	-0.0332074	-0.0305681	0.1057141	-0.0990171
TCGA-24-14	0.00680866	0.03747948	-0.0084256	0.01974677	0.06418925	-0.0572198
TCGA-24-14	0.02939645	0.00602866	0.06460036	-0.0409574	-0.0542297	0.04418519
TCGA-24-14	0.035944	-0.0343739	0.01829728	-0.0401984	-0.0505158	0.01478186
TCGA-24-14	0.04203238	0.08321754	0.10626017	0.02266037	-0.0616782	-0.0546082
TCGA-24-14	0.01605833	0.02177248	0.00823007	-0.0507233	-0.0857243	0.05123032

TCGA-24-14	0.00525055	0.05561134	-0.0522281	0.04231926	-0.0529204	-0.0342579
TCGA-24-15	0.00360034	-0.0854759	-0.0476986	-0.0015437	-0.0558384	0.07669762
TCGA-24-15	-0.0689842	0.04086581	-0.0559615	-0.0014297	0.09492505	-0.0568166
TCGA-24-15	0.08933875	-0.0064609	0.07884666	-0.0375563	-0.0588804	0.00203343
TCGA-24-15	-0.0656035	0.02746307	-0.0370849	0.07726386	0.08600055	-0.0846013
TCGA-24-15	0.01953558	0.06732343	0.0685711	-0.0799209	-0.067798	-0.0339962
TCGA-24-15	-0.0066494	-0.0260597	-0.0425023	0.04828171	-0.014345	0.04705432
TCGA-24-15	0.00709858	0.09569627	0.15242248	-0.093126	-0.0700199	-0.05365
TCGA-24-15	-0.0068859	-0.033698	-0.0591305	0.01165005	-0.0277747	0.02487658
TCGA-24-15	0.0626996	-0.0562701	-0.0321839	0.00199088	-0.0498568	0.04625321
TCGA-24-15	-0.0290806	0.01371853	0.00741637	-0.0120917	0.02433728	-0.0369559
TCGA-24-15	-0.0229547	0.01337331	-0.0208609	0.03335769	0.08880669	-0.0694507
TCGA-24-15	0.04806654	0.05851572	0.04942972	-0.1374175	-0.1122491	0.03696739
TCGA-24-15	-0.0455084	-0.0927732	-0.0800826	0.0279368	-0.0010062	0.03410084
TCGA-24-16	0.03659943	-0.0312416	-0.0294748	0.03780533	-0.0461528	0.01557623
TCGA-24-16	0.09922275	-0.0106147	-0.0125694	-0.0500873	-0.0391168	-0.003418
TCGA-24-16	-0.0322293	-0.0551343	-0.04332	-0.0110684	-0.0401484	0.06401371
TCGA-24-18	0.02301933	0.06364077	0.10665221	-0.051239	-0.012269	-0.0432354
TCGA-24-18	0.01066468	0.06404599	0.05618759	-0.0291417	-0.0450325	-0.0144696
TCGA-24-18	-0.0437519	-0.0577725	-0.0870257	0.07418605	-0.0141651	0.02714058
TCGA-24-18	0.06238888	0.04399472	0.03604076	-0.1145994	-0.0031609	-0.0193065
TCGA-24-18	0.00952586	0.09356934	0.13829393	-0.037485	-0.0388644	-0.0682152
TCGA-24-19	-0.0027118	-0.028154	-0.0711413	-0.0101742	0.04437547	-0.0148532
TCGA-24-19	0.06099161	0.08126513	0.08051139	-0.0247225	-0.0540992	-0.0365685
TCGA-24-19	-0.026844	0.01082127	-0.0367155	0.0624981	0.07441788	-0.065546
TCGA-24-19	0.03810048	0.10028854	0.05800761	0.02328499	0.0118278	-0.0654197
TCGA-24-20	-0.0422966	-0.0209071	-0.0383225	-0.0094415	0.0363456	-0.0189312
TCGA-24-20	0.07766165	0.03619454	0.05206976	-0.0327067	-0.0442847	-0.013975
TCGA-24-20	-0.030675	-0.0765929	-0.0796187	0.04192297	-0.00177	0.00781636
TCGA-24-20	-0.0913329	-0.0013081	-0.0453201	-0.0474356	0.0390035	0.00371129
TCGA-24-20	-0.0599413	0.04876833	0.01286017	0.1354626	0.07773212	-0.0872606
TCGA-24-20	-0.0682293	-0.0515968	-0.0992157	0.05114758	-0.0132897	0.00282345
TCGA-24-20	-0.0643053	-0.0299927	-0.0435306	-0.0458066	-0.0403354	0.02527585
TCGA-24-22	-0.0781328	0.03409983	-0.1163774	0.00318288	0.02921906	-0.0870702
TCGA-24-22	-0.0064286	0.05929273	-0.043288	-0.0253545	0.08447982	-0.0879591
TCGA-24-22	0.0191033	0.08219211	-0.0241925	0.01750428	-0.0320134	-0.0351615
TCGA-24-22	0.05758484	-0.0711088	-0.0359651	0.08871507	-0.0166542	0.0257274
TCGA-24-22	0.04429755	-0.0263205	-0.0308433	0.00452384	0.06602306	-0.0033958
TCGA-25-13	0.01262103	0.00394769	-0.0500905	-0.0054301	-0.0296853	0.00382184
TCGA-25-13	0.07685176	0.06760105	-0.0408111	-0.0123144	-0.010052	-0.0551336
TCGA-25-13	0.02912926	-0.0953414	-0.0804079	0.03891397	-0.032187	0.06012347
TCGA-25-13	-0.0436614	-0.0542012	0.01490293	0.01577771	0.01860173	-0.0021116
TCGA-25-13	-0.0449698	-0.0669121	-0.0552044	0.00693025	0.04599114	-0.0163095
TCGA-25-13	-0.0412179	0.02111963	-0.0679793	-0.0316298	0.0288073	-0.0373624
TCGA-25-13	0.05848085	0.00991238	-0.0005043	0.0596891	-0.0414452	-0.0062189
TCGA-25-13	0.04622026	0.02825301	-0.0700864	-0.0083182	0.00367775	-0.0510521
TCGA-25-13	-0.0751237	0.01048287	-0.0362926	-0.0030404	0.07517633	-0.0830956
TCGA-25-16	0.03477756	0.06903816	0.07227736	-0.0428322	0.07580984	-0.104019
TCGA-25-16	-0.0328305	0.06152299	0.01860382	-0.0067886	0.12174373	-0.1505346
TCGA-25-16	-0.0392985	-0.0222557	-0.0418018	-0.0194929	-0.0158521	0.00479211
TCGA-25-16	-0.0025749	0.03584866	0.05553367	-0.0304291	0.08279532	-0.116196

TCGA-25-16	-0.0360823	0.09379587	-0.022932	-0.0264856	-0.030605	-0.0593949
TCGA-25-16	-0.0601766	-0.0910638	-0.0757845	0.04020771	-0.0153667	0.05790772
TCGA-25-16	-0.0020395	0.03147112	0.03346587	-0.0042813	0.07971625	-0.0929086
TCGA-25-16	-0.1065629	-0.1163778	-0.0577468	0.00630045	-0.0154785	0.11634448
TCGA-25-16	0.0105731	0.04673292	0.01388681	-0.0367913	0.05874806	-0.0784938
TCGA-25-18	-0.032924	0.01700433	-0.0532655	-0.00055	-0.0388314	-0.0134213
TCGA-25-18	-0.0343086	-0.0297795	0.01657893	-0.0237038	0.01243794	0.01903378
TCGA-25-20	-0.0993866	0.01367758	-0.0426055	0.09785837	0.15772665	-0.1020569
TCGA-25-23	0.04480106	0.02299913	-0.0008504	-0.0816892	-0.068452	-0.0073737
TCGA-25-23	0.03271552	0.12316715	0.01271093	-0.0454768	-0.0628008	-0.0392686
TCGA-25-23	-0.0636321	0.00269604	-0.0574669	-0.0208843	0.11025278	-0.0761624
TCGA-25-24	0.01806273	0.09884115	0.01262932	0.03752756	0.05304904	-0.0925034
TCGA-25-24	0.02062928	0.00717071	-0.0184323	0.01384329	0.05102759	-0.045785
TCGA-25-24	-0.0001221	0.04983733	0.01864614	-0.0183474	0.00470722	-0.0285655
TCGA-29-16	0.01708189	-0.0049661	-0.0007861	-0.0001069	-0.0173034	-0.0120642
TCGA-29-16	0.03116506	-0.0123455	-0.0101119	0.03075454	0.04400253	0.00569622
TCGA-29-16	-0.0413125	0.06628431	-0.0167987	0.03177545	0.06088164	-0.0767426
TCGA-29-16	0.03602193	-0.0297656	0.02146486	0.00211047	-0.07206	0.07411993
TCGA-29-17	-0.0028056	-0.0315981	-0.0164716	0.0052549	-0.015827	0.00838186
TCGA-29-17	-0.0836634	0.03122698	-0.0372344	0.04532949	0.07132023	-0.0377081
TCGA-29-17	-0.0035979	0.09342037	0.11759849	-0.0802332	0.01588955	-0.0724182
TCGA-29-17	-0.0036665	0.04244141	0.05207698	-0.0642166	-0.0617599	0.04743511
TCGA-29-17	0.01470278	-0.0154312	0.01552508	-0.0389157	0.05722243	-0.0037063
TCGA-29-17	0.00664794	0.04495126	0.05595452	-0.0233069	0.08081967	-0.0326134
TCGA-29-17	0.02703106	0.02010219	0.0130173	-0.0477919	0.00574342	0.0204609
TCGA-29-17	-0.0882067	-0.1004751	-0.0973535	0.11777644	0.02636469	0.03730538
TCGA-29-17	0.0057085	-0.0139222	-0.0080468	-0.0303505	0.0278622	-0.0156753
TCGA-29-17	0.0077001	0.01917828	0.02879167	-0.0278356	0.03670289	0.01019165
TCGA-29-17	0.03600978	0.01067451	0.07568841	0.04926474	-0.0463955	-0.0144739
TCGA-29-17	0.03931266	0.09474884	0.15685673	-0.0433094	-0.0690329	-0.0216806
TCGA-29-17	0.02670432	0.08645038	0.1151448	-0.0801493	-0.0868461	0.00035188
TCGA-29-17	0.04833454	0.07348265	0.12112791	-0.0509875	-0.0447412	-0.0103272
TCGA-29-24	-0.0705379	-0.0585401	-0.0674292	0.01258932	0.07485703	0.01270447
TCGA-29-24	-0.0580306	-0.0296813	0.00540816	0.00166185	-0.0118693	0.02116705
TCGA-29-24	0.03640558	0.11873185	0.1352725	-0.0755846	-0.0345897	-0.0371621
TCGA-29-24	-0.0107679	0.05367375	0.1164571	-0.0702313	-0.045547	0.00098016
TCGA-30-17	-0.0763938	-0.0533159	-0.0217697	-0.0449114	-0.046394	0.09517102
TCGA-30-17	0.02239065	0.01620221	0.01948852	0.01254502	0.0475403	-0.0153763
TCGA-30-18	-0.0661153	-0.114242	-0.0380073	0.00082373	-0.0231871	0.12981123
TCGA-30-18	0.04277306	0.07724565	0.14499196	-0.0713465	-0.0336252	-0.0146826
TCGA-30-18	-0.0938088	-0.0529708	-0.0664615	0.04503756	0.12337177	0.0052313
TCGA-30-18	-0.0302163	-0.0057677	-0.0063301	0.02336781	0.06618627	-0.0484771
TCGA-30-18	0.01396585	-0.0079642	0.06477558	-0.035232	-0.0632302	0.05735517
TCGA-31-19	-0.0429575	0.01041247	-0.0024357	0.01743144	0.02374013	-0.0291464
TCGA-31-19	0.01611862	0.05560751	0.00320094	0.02633229	0.05704632	-0.0717874
TCGA-31-19	0.10784011	0.02344497	0.05754619	0.00885824	-0.0887319	0.01841495
TCGA-31-19	0.05125215	0.04214106	0.02952642	-0.007413	0.03992657	-0.0865185
TCGA-31-19	0.01686618	0.11363323	0.09490568	-0.009524	0.00227322	-0.0834545
TCGA-31-19	-0.0006464	0.127677	0.14345905	0.02091481	0.0189678	-0.0927143
TCGA-31-19	-0.0047066	0.05222166	0.00039582	0.01560642	0.06904101	-0.1124748
TCGA-36-15	-0.0319291	0.05600793	0.05500633	-0.0792893	0.06182912	-0.0934485

TCGA-36-15	0.0472184	-0.0787147	-0.0610469	0.03916126	-0.0210872	0.03344591
TCGA-36-15	-0.0684358	0.06192417	0.05087634	0.01615196	0.09446217	-0.1009863
TCGA-36-15	-0.0445223	0.02539907	0.01711452	-0.0632401	0.07253646	-0.0643646
TCGA-57-15	-0.0866613	-0.0132511	-0.0022192	-0.0034668	0.03455731	0.01003222
TCGA-57-15	0.05208143	-0.0699226	-0.0436415	0.06572345	0.01154431	0.05337691
TCGA-57-15	0.02779351	0.02253131	-0.023043	-0.028084	-0.0508399	0.00949827
TCGA-57-15	-0.0658164	0.03119742	-0.0031585	0.02420174	0.0996101	-0.095896
TCGA-57-15	0.06673254	-0.0261828	-0.0034382	-0.0404259	-0.0737348	0.02072766
TCGA-57-19	0.0283985	0.00600553	-0.0710785	-0.0410305	-0.0325091	-0.0415695
TCGA-59-23	0.03746532	0.00450078	-0.0102645	0.0808352	0.00693962	-0.0163268
TCGA-59-23	0.05940722	-0.0081448	-0.0294694	-0.0365847	-0.0248087	0.00324332
TCGA-59-23	-0.0009695	0.0551224	0.05071892	-0.0028282	-0.0090266	-0.0542057
TCGA-59-23	0.00021823	0.04188791	0.04071296	-0.0014745	0.06802328	-0.0908396
TCGA-59-23	0.02224779	-0.0066215	0.00568374	-0.0186176	0.00671276	-0.0155805
TCGA-59-23	-0.0299381	-0.060837	-0.0682517	-0.0091203	0.00048356	0.01365692
TCGA-61-17	-0.0789434	-0.0053888	-0.0616517	0.29395014	0.11400003	-0.0728837
TCGA-61-17	0.0144362	-0.0028255	-0.012459	-0.0137634	0.01794391	-0.0269583
TCGA-61-17	0.00116335	0.03037753	0.02160986	0.02278809	0.01725392	0.00496734
TCGA-61-17	-0.0588421	-0.0084719	-0.0425197	-0.0171396	-0.0328689	0.00604257
TCGA-61-17	0.00143027	0.04187173	-0.0287887	0.05760842	-0.0290797	-0.0313534
TCGA-61-19	-0.0815453	-0.0866217	-0.0649913	0.07209426	0.02302859	0.01243004
TCGA-61-19	0.11144176	0.07370895	0.15433924	-0.0375786	-0.1134145	0.01804484
TCGA-61-19	0.00352017	0.02776685	0.00743792	-0.0080701	-0.0294506	0.01202321
TCGA-61-19	-0.0203328	0.06285681	0.00240805	-0.017572	0.06464483	-0.0724171
TCGA-61-19	-0.0624534	0.02960239	-0.0590996	0.07372221	0.04886074	-0.0562485
TCGA-61-20	0.01714777	0.01603112	0.0169353	-0.0069799	-0.0051357	-0.0007928
TCGA-61-20	-0.0589875	0.06406789	-0.0128966	0.02302182	0.06343826	-0.1108878
TCGA-61-20	-0.0567856	0.02837312	0.00385161	0.02937316	0.06911537	-0.0418975
TCGA-61-20	-0.0670121	-0.0433426	-0.0789899	0.02415148	-0.051608	0.04459779
TCGA-61-20	-0.0137787	-0.061887	-0.0040426	0.02956394	-0.0896836	0.13009277
TCGA-61-20	-0.0935221	-0.1161098	-0.1213499	0.0877485	0.03100265	0.00402059
TCGA-61-21	-0.0789256	-0.0517704	-0.0547571	0.0118393	0.06770395	0.00495988
TCGA-61-21	-0.1189298	-0.0107689	-0.114304	0.17481511	0.0917093	-0.0380876
TCGA-61-21	-0.0597049	-0.0227384	-0.042262	-0.0724888	-0.0392124	0.03719009
TCGA-61-21	-0.0169527	0.00718497	-0.0003021	0.03538081	-0.0287673	-0.030364
TCGA-WR-A	0.04433961	-0.1118544	-0.0598834	0.03536734	-0.0359489	0.06902288
TCGA-04-13	-0.0589602	0.0299492	0.05905136	-0.0198511	0.06518154	-0.0061804
TCGA-04-13	-0.0372011	0.00880023	0.02242712	-0.0149513	0.02055147	0.01223868
TCGA-04-13	-0.0936677	-0.089687	-0.0306774	-0.0020597	0.00897984	0.11481327
TCGA-04-13	0.03885979	0.00347381	0.01283968	0.0082585	-0.0073454	0.00019712
TCGA-04-13	-0.0413298	-0.127787	-0.0601019	0.07233145	-0.0249164	0.09935807
TCGA-04-13	0.07785372	0.04511486	0.08259732	-0.0235372	-0.0695975	-0.0048821
TCGA-04-15	-0.0305859	-0.1389986	-0.1145687	0.11753477	0.03294983	0.01373963
TCGA-04-15	-0.0303052	0.00064021	-0.019508	-0.040762	-0.0487423	0.07734838
TCGA-04-16	0.01779934	-0.0321146	0.01584492	-0.0374882	-0.0706743	0.06424314
TCGA-09-03	0.0057193	0.14215997	-0.0017428	-0.1077837	-0.1062119	-0.0131764
TCGA-09-03	-0.0198134	0.01465948	0.03605449	-0.0648545	-0.0949934	0.07460802
TCGA-09-16	0.03434135	0.00779112	0.01078832	0.02561776	-0.028175	0.04173161
TCGA-09-16	-0.0104228	-0.001	0.05503477	0.00173367	-0.0074883	-0.0292345
TCGA-09-16	0.035211	0.05953597	-0.0180607	-0.1005453	-0.0789449	-0.0109387
TCGA-09-16	-0.007945	-0.0165094	-0.0475858	0.07959572	-0.0303169	-0.0053029

TCGA-09-20	-0.1246917	-0.0823249	-0.036427	0.04130379	-0.0013169	0.0881045
TCGA-09-20	-0.0206947	0.0305714	0.03020669	-0.0540823	-0.0565548	0.02803083
TCGA-10-09	-0.0489803	-0.0873965	-0.0446123	0.03463352	0.02907063	0.06497213
TCGA-10-09	0.04577526	0.03014441	0.06447601	-0.0587055	-0.0558791	0.05981732
TCGA-13-07	-0.0812864	0.00283452	0.02578589	0.02383648	0.05142981	0.01144074
TCGA-13-07	-1.28E-06	0.03187836	0.03580891	-0.0813404	-0.0984738	0.08772799
TCGA-13-07	0.02479155	-0.005354	-0.0020876	-0.0665926	-0.029346	0.05952548
TCGA-13-07	0.0295097	0.08049177	0.14039816	0.04673903	0.05324368	-0.0868582
TCGA-13-07	0.03666989	0.09636125	0.04976271	-0.0517352	-0.0650197	0.02305758
TCGA-13-07	0.07370652	0.02611429	0.06381541	-0.0669369	-0.0946529	0.06226423
TCGA-13-08	-0.0164045	0.07054	0.03795969	0.00728931	-0.0876536	0.03447887
TCGA-13-08	0.05413805	0.00250021	0.1003408	-0.0856101	0.00301681	0.03089074
TCGA-13-08	0.04568182	0.09059447	0.12237014	-0.0871796	-0.062701	0.01227434
TCGA-13-08	0.05838619	-0.0147368	-0.0058056	-0.0284564	-0.0258624	0.06017976
TCGA-13-08	-0.0970643	-0.1242191	-0.0747447	0.08578482	0.02586249	0.08977505
TCGA-13-08	0.04596718	-0.0029451	0.07244708	-0.040826	0.00922533	0.01033082
TCGA-13-09	0.04291533	-0.0013137	-0.0010501	0.0026851	0.07979972	-0.0008034
TCGA-13-09	0.08443702	-0.0056922	0.06902545	0.08225048	-0.0475235	0.00501171
TCGA-13-09	0.10472428	-0.016577	0.04409921	-0.0179938	-0.080758	0.07244522
TCGA-13-09	-0.01968	0.05855164	0.08064426	-0.0406849	-0.0158106	-0.018235
TCGA-13-14	0.0192204	-0.0480444	-0.0297504	0.05185982	0.01746815	0.03584568
TCGA-13-14	-0.036153	0.01038699	0.01982723	0.04616227	0.06458678	-0.0496419
TCGA-13-14	-0.025787	-0.0225341	-0.0107766	0.06600243	0.1030928	-0.0328879
TCGA-13-14	-0.080463	-0.0780094	-0.033658	-0.0385603	0.02088377	0.07945155
TCGA-13-14	0.08260526	-0.0135519	0.06803201	-0.0465328	-0.0309727	0.0248683
TCGA-13-14	0.05458925	0.02131177	0.06687441	-0.0919039	-0.0422621	0.02370582
TCGA-13-14	0.00081701	0.03691262	0.11275912	-0.0308189	0.03397684	-0.0655489
TCGA-13-A5	0.07662593	-0.0686748	-0.0196151	-0.00638	-0.0449953	0.05104855
TCGA-20-09	0.03166479	0.13032573	0.11685495	-0.0791679	-0.0889662	0.02407185
TCGA-20-16	-0.0465037	0.02605824	0.03981412	0.03045785	0.05183661	-0.0458209
TCGA-20-16	-0.0219458	-0.0386059	0.00394031	0.01569208	0.0123781	-0.0064037
TCGA-20-16	-0.0041598	-0.0545342	-0.0163442	0.11880275	-0.0071297	0.07304343
TCGA-23-10	0.08930461	-0.0489337	-0.0039839	-0.0310296	-0.0833801	0.07582892
TCGA-23-10	0.02866963	0.04331234	0.05736114	-0.0267285	0.00977057	-0.016982
TCGA-23-10	0.04623456	-0.0196533	-0.0090244	0.00647679	-0.0182508	0.02125046
TCGA-23-11	-0.0659605	0.02033853	-0.0053133	0.04659314	0.02422836	-0.0198872
TCGA-23-11	-0.0825598	-0.029886	-0.0616039	-0.0312313	0.01696338	0.07388117
TCGA-23-11	0.03811219	-0.0141582	-0.0477133	-0.0126826	-0.0067633	-0.0142644
TCGA-23-20	-0.0459745	0.01270734	0.06617181	0.02107727	0.07753643	-0.0800241
TCGA-24-11	0.02351805	-0.0742261	-0.0073064	0.05282832	-0.0473805	0.08188288
TCGA-24-14	-0.0482223	0.02094444	-0.0275177	0.16779174	0.11613524	-0.1056256
TCGA-24-14	-0.0827431	0.03696943	0.02195497	-0.0072691	0.08761032	-0.0745298
TCGA-24-14	0.04777373	0.07848811	0.0621927	-0.0462657	-0.0616935	-0.0075404
TCGA-24-14	0.04136491	-0.0276042	-0.0109923	0.00143818	-0.0418253	0.01377798
TCGA-24-15	0.02549526	0.00121034	-0.0440573	0.00257806	-0.0471402	-0.0032445
TCGA-24-18	0.10585628	0.0293448	0.01454267	-0.0520229	-0.1081521	0.07977748
TCGA-24-18	-0.0595256	0.01928397	0.00155184	-0.0673506	0.04617839	-0.0322732
TCGA-24-20	-0.0928471	-0.0689219	-0.0566305	0.11869954	0.00896321	0.04918027
TCGA-24-20	0.00400835	0.01850687	0.02853718	-0.0622579	-0.0631544	0.02643086
TCGA-24-22	0.01425651	0.02376836	0.07442922	-0.0164523	0.02705957	-0.0527986
TCGA-24-22	-0.1004439	-0.0199016	-0.0559996	0.14966145	0.11743787	-0.0746401

TCGA-25-13	-0.0142926	-0.0010113	-0.0499423	0.00564072	0.09220152	-0.077888
TCGA-25-13	0.01392238	-0.0140467	-0.0572143	-0.0178856	-0.0466508	0.02383533
TCGA-25-13	-0.0218774	-0.0578422	-0.0936875	0.05322247	0.041399	-0.0064128
TCGA-25-13	-0.0519067	0.05771178	-0.0108955	-0.0047024	0.062208	-0.0838471
TCGA-25-13	-0.0410153	0.02487818	-0.0430024	0.00546606	0.05298552	-0.0510326
TCGA-25-23	-0.0822406	-0.0193566	-0.0111541	-0.0187392	-0.0044542	0.01210714
TCGA-25-23	0.07990254	0.01735031	0.04653103	-0.0145429	-0.0175264	-0.0112337
TCGA-25-23	0.04052988	0.027049	0.01599352	0.00522406	0.02367203	-0.0519154
TCGA-25-24	0.01545951	-0.0044283	-0.0556279	-0.0461479	-0.0583269	0.019018
TCGA-29-16	0.06964137	0.08384478	0.04251659	-0.0340245	-0.0049502	-0.0366961
TCGA-29-16	-0.0852208	0.07871331	0.01685953	-0.0824403	0.0071184	-0.0523981
TCGA-29-16	0.00099074	0.00276193	-0.0245409	-0.0073665	0.03501827	-0.0334518
TCGA-29-16	0.10164926	-0.0625199	-0.0400483	0.01634936	-0.0632602	0.04019266
TCGA-29-17	0.00338729	0.03590314	0.03731984	-0.0194616	0.00976703	0.01059738
TCGA-29-17	0.04451676	0.05807478	0.08166013	0.01065364	-0.0161559	-0.0434123
TCGA-29-17	-0.0659631	-0.0221049	-0.0988559	0.09435052	-0.0152918	0.02509414
TCGA-29-17	-0.0484655	-0.018454	-0.0530582	0.09967332	0.07884196	-0.0027885
TCGA-29-17	-0.0287017	0.05034952	-0.0064993	-0.0640474	-0.0031657	-0.0482395
TCGA-29-A5	-0.0311311	-0.0556377	-0.060672	0.03573176	0.09429274	-0.0559434
TCGA-30-18	-0.059174	0.0224222	0.00971078	-0.0032798	0.07281234	-0.0153318
TCGA-30-18	-0.0857628	-0.0098232	-0.0255992	0.13162384	0.10385199	-0.0054514
TCGA-36-15	0.00902601	0.02891088	0.02044324	-0.0208922	-0.0024482	-0.04093
TCGA-36-15	0.09634314	-0.0212902	-0.018958	0.00196828	-0.0508772	0.03157572
TCGA-36-15	0.02996882	0.01808894	-0.0045	0.01453484	0.03319331	-0.0430823
TCGA-59-23	0.07701483	-0.0150913	0.01102934	-0.0152633	-0.0008876	-0.0419768
TCGA-61-17	0.00324214	-0.0215175	-0.0485509	-0.0306935	0.04499917	0.00741241
TCGA-61-17	-0.0218983	0.02524169	-0.0303331	0.03224924	0.0859795	-0.056168
TCGA-61-17	0.11184993	-0.0059207	0.01573616	-0.0366151	-0.0816216	0.06286536
TCGA-61-17	0.07201232	-0.015366	0.01278705	-0.010703	-0.0307629	-0.0467702
TCGA-61-19	0.00069433	0.04025767	-0.0073924	0.00189867	-0.0356323	-0.0286826
TCGA-61-19	-0.024835	0.06827994	0.05674312	-0.0627765	-0.0765307	-0.0489603
TCGA-61-20	-0.0035341	0.07264736	0.09014675	-0.0811238	0.03308637	-0.0803043
TCGA-61-21	0.04446415	0.00820102	-0.004484	-0.0475359	-0.0708858	0.00505755

red
0.03007442
-0.0403412
0.05919813
-0.0285214
0.01457512
-0.0035178
0.05447525
0.00596684
0.00709299
0.0177398
-0.0357122
0.0130154
-0.0101573
0.05040073
0.01151665
-0.060613
0.09615952
0.05616654
-0.0380786
-0.0043597
-0.0338442
0.06835039
0.03862596
-0.0140007
-0.0979886
-0.0377211
-0.0422707
-0.006615
0.00086706
0.09074704
-0.0026378
-0.0851661
-0.051476
-0.0670001
-0.0525926
-0.0292439
0.03431002
-0.0123784
-0.1368537
0.02499718
-0.0206696
0.06989465
0.04753189
-0.0134999
-0.0710562
-0.0251558
-0.0456994
0.00973162
0.02109977
0.05477028

0.00246444
-0.0258494
-0.02113
-0.0296243
0.02144028
0.00356129
-0.1328542
-0.0600506
-0.0026144
0.04371468
-0.0202686
-0.0003928
-0.0160099
-0.040645
0.01069337
-0.0749022
-0.0614494
-0.0068572
0.06679961
0.07371964
0.04891442
0.08791913
-0.0999489
-0.017784
-0.0662751
-0.0159748
-0.0123502
-0.0308847
0.00806045
-0.0616987
0.00183895
0.01573597
-0.0017331
-0.0148506
0.00581976
0.03964112
0.00317507
-0.0156802
0.03003093
0.13564345
0.04179414
0.05136112
0.01263676
0.0751947
0.00184041
-0.0312659
-0.0150427
-0.0195521
0.05463121
-0.0189032
0.02364629

0.10688375
0.06025501
-0.046272
0.02314785
-0.0122651
-0.0178854
0.05665062
-0.0459265
0.11180127
0.07358508
-0.0115233
-0.0384021
0.0028779
0.1028187
0.03611798
0.04318118
0.05907242
-0.0210816
-0.0248939
0.0564766
-0.0153457
-0.0547376
-0.0202669
-0.0646946
0.03334943
-0.0524991
0.00858829
0.02572934
0.07790785
0.00425167
-0.0795224
0.08131636
0.036041
0.03151729
0.02547794
0.03376495
0.05452579
-0.0091306
0.04119273
-0.0183436
0.0938441
0.06751354
0.04450182
0.03913986
0.04144315
0.01447586
0.00562268
-0.0598944
-0.0421479
0.08633016
-0.009442

-0.053753
0.08356377
-0.0028741
0.12557349
-0.0187221
0.05602795
-0.0168361
-0.0089597
0.01886592
-0.0057576
-0.0101852
-0.0635801
-0.0098532
-0.0153529
0.00792393
-0.0409275
0.01656568
-0.0128741
0.01966488
0.0079594
-0.0453063
-0.0434986
-0.0626095
-0.1055289
-0.0774416
0.09087302
0.00411616
-0.1161384
0.01080021
-0.0930506
-0.0731768
-0.0276095
0.03566498
0.06537035
-0.1068405
-0.055345
-0.0078895
-0.0560559
0.06073558
-0.0847216
0.03874451
0.02721045
-0.0450209
0.03766573
-0.0151423
0.01279131
0.0081041
-0.0420206
-0.1178235
-0.0127229
0.00449447

0.12184394
-0.0635613
-0.0379008
0.0533595
0.05295746
0.03945448
-0.0195752
0.0466412
0.07970405
0.03661821
0.04046755
-0.0021194
-0.0239907
-0.008561
0.05590671
-0.0458898
0.0445936
-0.063647
0.00220077
0.02068047
0.091173
-0.0523141
-0.0538042
-0.0518375
-0.0226541
0.02641472
-0.0242645
-0.0505168
0.11260965
0.09475662
0.1449158
0.03079376
0.04285011
0.03221231
0.06640879
0.14261963
-0.0653116
-0.0827636
0.02266315
-0.0460683
0.08883738
-0.0184973
0.14065359
-0.0242156
0.00751743
-0.0489043
-0.0482891
-0.0835399
-0.0173488
0.04266704
0.07127394

0.03294578
-0.0703536
0.01086666
-0.0849629
-0.0236016
-0.0757787
-0.0500607
-0.1471859
-0.0862544
-0.0064642
-0.0772331
-0.0602086
-0.0500981
-0.0681711
0.07993581
-0.008426
-0.0283836
-0.00827
-0.0342514
-0.0954429
0.02839774
-0.0263298
-0.0347305
0.00758298
-0.0549756
-0.0404769
-0.1031401
0.08313912
-0.1334409
-0.0168596
0.08954199
-0.0115046
0.03278738
-0.0871064
-0.0128273
-0.0144485
-0.0162231
0.02769951
-0.0009319
0.03572992
-0.0295255
-0.0022851
-0.050667
0.09213245
0.02091104
-0.0143913
-0.0129225
0.11552179
0.00410826
-0.0353947
-0.0202651

0.01601969
0.08203796
0.0304757
0.00339955
0.02152506
0.05013096
-0.0097868
0.01079049
0.08483161
-0.0567375
-0.0698155
-0.0205234
0.08297453
-0.0738479
0.00347754
0.04046019
-0.0498278
-0.0122525
0.15725424
-0.0535662
-0.0814053
0.01734706
0.05042908
-0.0196902
0.02374075
-0.0085594
-0.0260432
-0.028998
0.0456576
0.02252688
0.05040053
-0.0628008
0.0394021
