# Supplementary material for: The Immune Subtypes and Landscape of Advanced-Stage Ovarian Cancer
Source: Vaccines (Basel). 2022 Sep 2;10(9):1451. doi: 10.3390/vaccines10091451 (PMC9501495; doi:10.3390/vaccines10091451)
Supplement: Supplementary file 1 [file vaccines-10-01451-s001.zip › Supplementary Materials File/Supplementary Materials File S6.pdf]

Module	Correlation	
A2M	turquoise	0.244779
ABCA6	turquoise	-0.453832
ABCA8	turquoise	-0.3620334
ABCA9	turquoise	-0.397225
ABI3	turquoise	0.8872393
ABI3BP	turquoise	-0.2579843
ACE	turquoise	-0.2482712
ACHE	turquoise	-0.2575429
ACP5	turquoise	0.19311436
ACSS3	turquoise	-0.3061258
ACTN1	turquoise	-0.3623198
ACVR1	turquoise	-0.4566445
ACVR2A	turquoise	-0.6491958
ADAM8	turquoise	0.47577829
ADAMDEC1	turquoise	0.53696895
ADAMTS5	turquoise	-0.3337902
ADAP2	turquoise	0.82445838
ADAT1	turquoise	-0.3416082
ADCY4	turquoise	-0.0941375
AFF3	turquoise	-0.4176454
AGAP2	turquoise	0.11976428
AIF1	turquoise	0.87772407
CRYBG2	turquoise	-0.3602339
AKAP12	turquoise	-0.256814
AKNA	turquoise	0.07970975
ALDH1A1	turquoise	-0.2448225
ALOX5	turquoise	0.57345005
ALOX5AP	turquoise	0.83338062
AMHR2	turquoise	-0.4160814
JAML	turquoise	0.58335303
AMPH	turquoise	-0.1795589
ANGPTL1	turquoise	-0.3798434
ANK2	turquoise	-0.4581663
ANKRD44	turquoise	-0.1563836
AOAH	turquoise	0.76055177
AP1S2	turquoise	-0.3500975
AP3B1	turquoise	-0.5694899
APBB1IP	turquoise	0.90456169
APOBR	turquoise	0.68835319
APOC1	turquoise	0.53240738
APOE	turquoise	0.31500635
AQP9	turquoise	0.15659684
ARHGAP15	turquoise	0.64414797
ARHGAP18	turquoise	0.21927863
ARHGAP22	turquoise	-0.2992145
ARHGAP30	turquoise	0.57963731
ARHGAP6	turquoise	-0.285168
ARHGAP9	turquoise	0.86208705
ARHGDIB	turquoise	0.64344953
ARHGEF37	turquoise	-0.2397301

ARID5A	turquoise	-0.1059034
ARRB1	turquoise	0.13815818
ASXL3	turquoise	-0.6702803
ATE1	turquoise	-0.5712441
ATP8A1	turquoise	-0.2740826
ATP8B4	turquoise	0.19307837
B3GAT1	turquoise	-0.35654
BEND5	turquoise	-0.5570252
BEX5	turquoise	-0.3820655
BIN2	turquoise	0.90820956
BMP2	turquoise	-0.3061568
BMP7	turquoise	-0.4335237
BMPR1A	turquoise	-0.5996529
BMPR1B	turquoise	-0.4819693
BMPR2	turquoise	-0.5681546
BNC2	turquoise	-0.2964655
BST1	turquoise	0.22647056
BTK	turquoise	0.90590446
BTN2A2	turquoise	-0.1874512
VSIR	turquoise	0.40089831
CEP128	turquoise	-0.5826874
C15orf48	turquoise	0.36807843
MILR1	turquoise	0.62259323
SCIMP	turquoise	0.76115537
LDLRAD4	turquoise	-0.0468519
PEAK3	turquoise	0.19648566
C1orf116	turquoise	0.03629211
C1orf162	turquoise	0.71858453
THEMIS2	turquoise	0.15259994
C1QA	turquoise	0.81921198
C1QB	turquoise	0.83846828
C1QC	turquoise	0.85875542
TRABD2A	turquoise	-0.3765605
C3	turquoise	0.32132375
C3AR1	turquoise	0.92254617
C5AR1	turquoise	0.54048179
CREBRF	turquoise	-0.3955423
C6orf132	turquoise	-0.2943222
CCDC170	turquoise	-0.42207
C7	turquoise	-0.2710034
CYSRT1	turquoise	-0.4149241
CACNA1C	turquoise	-0.4021738
CACNA2D2	turquoise	-0.5633838
CACNA2D4	turquoise	0.24101616
CALR	turquoise	-0.6060959
CANX	turquoise	-0.4852245
CASS4	turquoise	0.20425526
CCDC102B	turquoise	-0.4761576
CCL14	turquoise	-0.3885539
CCL17	turquoise	0.18125349
CCL18	turquoise	0.19881883

CCL3	turquoise	0.52485857
CCL3L1	turquoise	0.35891092
CCL3L3	turquoise	0.35891092
CCL4	turquoise	0.70435981
CCL4L1	turquoise	0.51891678
CCR10	turquoise	-0.4618435
CCR1	turquoise	0.76149137
CCR5	turquoise	0.84532878
CCRL2	turquoise	0.65929021
CD14	turquoise	0.85754236
CD163	turquoise	0.79074954
CD180	turquoise	0.81535639
CD1A	turquoise	0.26169059
CD1B	turquoise	0.06706999
CD1C	turquoise	0.35734665
CD1E	turquoise	0.32773142
CD200	turquoise	-0.4197351
CD207	turquoise	-0.0420332
CD22	turquoise	-0.2735311
CD28	turquoise	0.0971462
CD300A	turquoise	0.82625128
CD300C	turquoise	0.81901682
CD300E	turquoise	0.32892134
CD300LB	turquoise	0.15324008
CD300LF	turquoise	0.82448853
CD33	turquoise	0.7282997
CD37	turquoise	0.85020786
CD48	turquoise	0.8362089
CD4	turquoise	0.77629378
CD53	turquoise	0.95440649
CD68	turquoise	0.57706187
CD69	turquoise	0.48059283
CD84	turquoise	0.85855905
CD86	turquoise	0.93740902
CDC42SE2	turquoise	-0.362805
CDH20	turquoise	-0.3492325
CDH3	turquoise	-0.4278682
CDH6	turquoise	-0.2023069
CDK15	turquoise	-0.4863316
CEACAM19	turquoise	-0.4044378
CEACAM21	turquoise	0.20793283
ADA2	turquoise	0.08012082
CERKL	turquoise	0.20082256
CHRDL1	turquoise	-0.1705624
CHST13	turquoise	-0.2019363
CHST2	turquoise	-0.1463493
CLCF1	turquoise	-0.247947
CLEC10A	turquoise	0.46557669
CLEC11A	turquoise	-0.240406
CLEC4A	turquoise	0.78126569
CLEC4E	turquoise	0.53771499

CLEC5A	turquoise	0.62851327
CLIC2	turquoise	0.27374756
CLIC3	turquoise	-0.2354319
CLIP3	turquoise	-0.4538673
CLOCK	turquoise	-0.3627498
CNTFR	turquoise	-0.4073
COL18A1	turquoise	-0.5126408
CORO1A	turquoise	0.61033881
COTL1	turquoise	0.21825849
CPNE5	turquoise	-0.140651
CPXM1	turquoise	-0.4070099
CR1	turquoise	-0.2459416
CR2	turquoise	-0.4248192
CRTAM	turquoise	0.25139679
CRYBB1	turquoise	0.27894313
CSF1	turquoise	0.54952589
CSF1R	turquoise	0.85696775
CSF2RA	turquoise	0.78102291
CSF2RB	turquoise	0.76244886
CSF3R	turquoise	0.52671722
CSGALNACT2	turquoise	-0.2261532
CTF1	turquoise	-0.4452201
CTSE	turquoise	-0.0202928
CTSL	turquoise	0.11460692
CTSS	turquoise	0.70403295
CTTNBP2	turquoise	-0.28341
CX3CL1	turquoise	0.08514477
CX3CR1	turquoise	0.44035702
CXCL1	turquoise	0.17392985
CXCL3	turquoise	0.00552648
CXCL5	turquoise	0.01286433
CXCL6	turquoise	0.03655833
CXCR4	turquoise	-0.204207
CXorf21	turquoise	0.79032058
CYBB	turquoise	0.91841213
CYFIP2	turquoise	-0.5373825
CYP27A1	turquoise	-0.2305427
CYTH4	turquoise	0.89506143
CYTIP	turquoise	0.6941597
DBH	turquoise	-0.5782059
DCBLD1	turquoise	-0.33224
DENND1C	turquoise	-0.1731331
DIXDC1	turquoise	-0.6080829
DLC1	turquoise	-0.3026696
DMKN	turquoise	-0.5029942
DOCK10	turquoise	0.58374634
DOCK2	turquoise	0.83165897
DOCK8	turquoise	0.5846832
DOK2	turquoise	0.7410333
DOK3	turquoise	0.64120358
DOK6	turquoise	-0.6151296

DPEP1	turquoise	-0.1875066
DPEP2	turquoise	0.33959207
DPP8	turquoise	-0.5228171
DUOXA1	turquoise	-0.3881929
DUSP4	turquoise	-0.129029
E2F5	turquoise	-0.5097366
EBF1	turquoise	-0.1752914
EBF2	turquoise	-0.3856759
EBI3	turquoise	0.6964471
EDA	turquoise	-0.3462501
EDAR	turquoise	-0.2732215
EDNRB	turquoise	-0.2067746
EFEMP2	turquoise	-0.4329732
EMILIN2	turquoise	0.01569283
ADGRE2	turquoise	0.54700284
ENO3	turquoise	-0.4735214
ENPEP	turquoise	-0.3330384
ENPP4	turquoise	-0.421065
EPOR	turquoise	-0.43992
EVI2A	turquoise	0.75783313
EVI2B	turquoise	0.90205795
EVPL	turquoise	-0.2552982
FCMR	turquoise	0.32501575
PCED1B	turquoise	0.2908892
FAM13C	turquoise	-0.4969999
FAM171B	turquoise	-0.1772165
TVP23A	turquoise	-0.348225
CALHM6	turquoise	0.49386101
STRIP2	turquoise	-0.3870057
FAM49A	turquoise	0.05009176
MINDY2	turquoise	-0.4107956
FAM78A	turquoise	0.63458938
FBLN5	turquoise	-0.3063733
FBP1	turquoise	0.3435449
FCER1A	turquoise	0.30275005
FCER1G	turquoise	0.79015866
FCGBP	turquoise	0.30076392
FCGR1A	turquoise	0.85789728
FCGR1B	turquoise	0.51080396
FCGR1CP	turquoise	0.63093455
FCGR2A	turquoise	0.86412281
FCGR2B	turquoise	0.80496026
FCGR2C	turquoise	0.86412281
FCGR3A	turquoise	0.83076772
FCGRT	turquoise	-0.3338189
FCN1	turquoise	0.37510251
FCRL6	turquoise	0.15000145
FERMT2	turquoise	-0.4335755
FERMT3	turquoise	0.9167622
FGD2	turquoise	0.41560679
FGD3	turquoise	0.06472045

FGL2	turquoise	0.82575662
FGR	turquoise	0.7399801
FHL5	turquoise	-0.4470379
FICD	turquoise	-0.4375692
FKBP11	turquoise	-0.4422189
FKBP7	turquoise	-0.5282351
FLI1	turquoise	0.54140067
ANKRD36BP2	turquoise	-0.4765227
FLVCR2	turquoise	0.12811049
FMNL1	turquoise	0.38634013
FOLR2	turquoise	0.43245527
FPR1	turquoise	0.7317749
FPR3	turquoise	0.77625251
FRZB	turquoise	-0.3309272
FYB1	turquoise	0.88151387
FYN	turquoise	-0.484842
FZD4	turquoise	-0.2623655
GAB3	turquoise	0.70164381
GAPT	turquoise	0.579309
GATA2	turquoise	-0.1978094
GATM	turquoise	-0.2768114
GDF5	turquoise	-0.3986102
GFRA3	turquoise	-0.4860751
GGTA1P	turquoise	0.14175856
GHR	turquoise	-0.3111486
GIMAP1	turquoise	0.52892163
GIMAP2	turquoise	0.49317217
GIMAP4	turquoise	0.81484777
GIMAP6	turquoise	0.71988044
GIMAP7	turquoise	0.60928633
GIMAP8	turquoise	0.57328587
GJA4	turquoise	-0.3752782
GJB3	turquoise	-0.0557871
GJD3	turquoise	-0.3207731
COLGALT2	turquoise	-0.5378118
GMFG	turquoise	0.66092225
GNA15	turquoise	0.78477208
GNGT2	turquoise	0.39355908
GPIHBP1	turquoise	-0.3698239
GPR132	turquoise	0.16179058
ADGRD1	turquoise	-0.5558279
GPR141	turquoise	0.34247697
GPR183	turquoise	0.72545543
GPR34	turquoise	0.76753922
GPR35	turquoise	-0.4325348
GPR65	turquoise	0.82321254
GPR82	turquoise	0.3016271
GPR84	turquoise	0.79997999
GPRIN3	turquoise	0.46158476
GPSM3	turquoise	0.47296676
GSDMA	turquoise	0.06668544

GTF2A1	turquoise	-0.5713104
GUCY1A2	turquoise	-0.2748937
HAMP	turquoise	0.33003713
HAPLN3	turquoise	-0.0507103
HAVCR2	turquoise	0.94340277
HCK	turquoise	0.88639772
HCLS1	turquoise	0.57326857
HDC	turquoise	-0.5208183
HFE	turquoise	-0.1221046
HIPK3	turquoise	-0.3138363
HIST1H2AG	turquoise	-0.2610758
HK3	turquoise	0.65470548
HLA-DQA1	turquoise	0.69641401
HLA-DQA2	turquoise	0.42548874
ARHGAP45	turquoise	0.5961249
HMSD	turquoise	-0.5226568
HNMT	turquoise	0.26813001
HPGD	turquoise	-0.130957
HPGDS	turquoise	0.43752967
HS3ST2	turquoise	-0.1558857
HSP90AB1	turquoise	-0.6333577
HSPA12B	turquoise	-0.3893925
HSPA1L	turquoise	-0.4822571
HSPA2	turquoise	-0.340093
HSPA4	turquoise	-0.5306145
HSPA5	turquoise	-0.5569769
HSPA6	turquoise	0.06454172
HYDIN	turquoise	-0.6204501
ICAM2	turquoise	-0.1624787
IFI30	turquoise	0.3299584
IFNAR1	turquoise	-0.2538572
IGF1	turquoise	-0.2774213
IGSF10	turquoise	-0.16545
IGSF21	turquoise	0.57743458
IGSF6	turquoise	0.74017125
IKZF1	turquoise	0.72300307
IL10RA	turquoise	0.8955783
IL11	turquoise	-0.4187864
IL11RA	turquoise	-0.4276365
IL12RB1	turquoise	0.68185149
IL16	turquoise	0.52121804
IL17RA	turquoise	0.08221038
IL17RB	turquoise	-0.5517643
IL18R1	turquoise	-0.3047245
IL1B	turquoise	0.57071688
IL1RAP	turquoise	-0.2072477
IL1RN	turquoise	0.24546424
IL22RA1	turquoise	-0.3481718
IL23A	turquoise	-0.2125794
IFNLR1	turquoise	-0.4636331
IL2RA	turquoise	0.5549565

IL3RA	turquoise	-0.1667328
IL6ST	turquoise	-0.3081673
CXCL8	turquoise	0.13226108
INHBB	turquoise	-0.2303118
INHBE	turquoise	-0.5235448
INPP5D	turquoise	0.5798444
IQGAP2	turquoise	-0.3824947
IRF8	turquoise	0.67865251
ITGA4	turquoise	0.52429451
ITGAL	turquoise	0.82511752
ITGAM	turquoise	0.81929981
ITGAX	turquoise	0.80168582
ITGB1	turquoise	-0.320193
ITGB2	turquoise	0.87969717
ITGB7	turquoise	0.26238924
JMY	turquoise	-0.5360722
JUP	turquoise	-0.419814
KCNAB2	turquoise	0.37736888
KCND2	turquoise	0.11495069
KCNH2	turquoise	-0.2757018
KCNK13	turquoise	0.22524399
KCNK6	turquoise	-0.0348481
KCNMB1	turquoise	0.22484667
KCNN3	turquoise	-0.4562578
KCNN4	turquoise	-0.0432234
KCNT2	turquoise	-0.46858
KCTD12	turquoise	0.02685719
KIAA1755	turquoise	-0.3382806
KIF21B	turquoise	-0.2961375
KIT	turquoise	-0.4257321
KITLG	turquoise	-0.3506073
KL	turquoise	-0.5252632
KLHL23	turquoise	-0.5955491
KLHL6	turquoise	0.19599543
KLRG1	turquoise	-0.5000296
KRT14	turquoise	-0.0437252
KRT16	turquoise	0.16302909
KRT6B	turquoise	-0.054824
LAIR1	turquoise	0.94780936
LAMA2	turquoise	-0.4549678
LAMC2	turquoise	-0.1605669
LAPTM5	turquoise	0.9318936
LAT2	turquoise	0.85198975
LATS2	turquoise	-0.395598
LCOR	turquoise	-0.5838278
LCP1	turquoise	0.38910381
LCP2	turquoise	0.9420509
LEP	turquoise	-0.4180862
LEPR	turquoise	-0.2695374
LGI2	turquoise	-0.3951827
LHFPL2	turquoise	0.46301045

LILRA1	turquoise	0.44674753
LILRA2	turquoise	0.47577559
LILRA5	turquoise	0.33454875
LILRA6	turquoise	0.26861987
LILRB1	turquoise	0.86606449
LILRB2	turquoise	0.84177176
LILRB3	turquoise	0.46550597
LILRB4	turquoise	0.90743732
LIMD2	turquoise	-0.2392434
LIME1	turquoise	-0.3376856
LIMS1	turquoise	-0.320095
LIPA	turquoise	0.17527035
PCED1B-AS1	turquoise	0.74309118
LINC00654	turquoise	-0.3130809
LINC00926	turquoise	-0.4433055
LOC730101	turquoise	-0.5758557
LPXN	turquoise	0.69030205
LRCH2	turquoise	-0.3611396
LRRC17	turquoise	-0.3117529
LRRC25	turquoise	0.85383146
LSP1	turquoise	0.8044758
LST1	turquoise	0.76446443
LY86	turquoise	0.78468342
LYL1	turquoise	0.41295488
LYN	turquoise	0.33706631
LYPD3	turquoise	-0.1670848
LYZ	turquoise	0.70662146
MAGEL2	turquoise	-0.4256424
MAN1A2	turquoise	-0.5142135
MAOB	turquoise	-0.3130432
MAP1LC3C	turquoise	-0.4198406
MAP7D1	turquoise	-0.2316991
MARCO	turquoise	0.34586403
MBNL3	turquoise	-0.4022718
SLC25A53	turquoise	-0.6389768
MEF2C	turquoise	0.33139572
MET	turquoise	-0.2652832
MFNG	turquoise	0.08105361
MITF	turquoise	-0.1189745
MMP12	turquoise	0.18032488
MMP16	turquoise	-0.5907285
MMP3	turquoise	-0.2618011
MMP9	turquoise	0.45291298
MMRN1	turquoise	-0.2057613
MNDA	turquoise	0.87902253
MPEG1	turquoise	0.82023951
MPL	turquoise	-0.5907333
MPP1	turquoise	0.53992715
MS4A14	turquoise	0.29983712
MS4A4A	turquoise	0.76957604
MS4A6A	turquoise	0.89964438

MS4A7	turquoise	0.76691445
MSR1	turquoise	0.77804739
MVP	turquoise	0.15297503
MYEF2	turquoise	-0.6019955
MYO1F	turquoise	0.73708775
MYO1G	turquoise	0.46848186
MYO9A	turquoise	-0.3775485
N4BP2	turquoise	-0.607686
NAALADL1	turquoise	-0.3864881
NAP1L3	turquoise	-0.5031317
NAPSB	turquoise	0.58816238
NBEA	turquoise	-0.5395417
NCCRP1	turquoise	-0.2216732
NCF1	turquoise	0.81316048
NCF1B	turquoise	0.71238319
NCF1C	turquoise	0.68914653
NCF2	turquoise	0.75304284
NCF4	turquoise	0.86589592
NCKAP1L	turquoise	0.88810095
NEGR1	turquoise	-0.3812137
NFAM1	turquoise	0.76023702
NFYB	turquoise	-0.5714349
NHLRC2	turquoise	-0.6277647
NLRC4	turquoise	0.30566709
NLRP3	turquoise	0.67305115
NR5A2	turquoise	-0.3555701
NRXN3	turquoise	-0.5313397
NTNG2	turquoise	-0.1644567
NTRK1	turquoise	-0.4801534
OGFRL1	turquoise	0.14502036
OLR1	turquoise	0.72968423
OSCAR	turquoise	0.73939322
OSM	turquoise	0.52740463
OSMR	turquoise	0.29452271
OVOL1	turquoise	-0.2744421
P2RX7	turquoise	0.50107237
P2RY12	turquoise	0.38154541
P2RY13	turquoise	0.6970638
PABPC5	turquoise	-0.5601916
PADI2	turquoise	0.15656772
PAFAH1B2	turquoise	-0.5951674
PARVG	turquoise	0.29378732
PATZ1	turquoise	-0.6088945
PBX4	turquoise	-0.5578971
PCDH17	turquoise	-0.2806945
PCDH18	turquoise	-0.3797146
PCYOX1L	turquoise	-0.3880205
PDCD1LG2	turquoise	0.68057865
PDE1A	turquoise	0.00776587
PDE1B	turquoise	-0.1015363
PDE3A	turquoise	-0.4172485

PDE3B	turquoise	-0.3891246
PDE4B	turquoise	0.42345958
PDGFA	turquoise	-0.3289745
PDGFC	turquoise	-0.3230179
PDIA2	turquoise	-0.3815097
PDIA3	turquoise	-0.5054133
PDZK1IP1	turquoise	0.3368247
PEG3	turquoise	-0.4314871
PGM5	turquoise	-0.3152231
PHACTR1	turquoise	-0.2847726
PIK3AP1	turquoise	0.5353752
PIK3CG	turquoise	0.65293964
PIK3R5	turquoise	0.66209005
PIK3R6	turquoise	0.25896746
PILRA	turquoise	0.35552339
PKHD1L1	turquoise	-0.1291166
PKIB	turquoise	0.31861956
PLA1A	turquoise	-0.2554828
PLAC9	turquoise	-0.233223
PLCB2	turquoise	0.39889387
PLCB4	turquoise	-0.6226516
PLCL1	turquoise	-0.496712
PLD4	turquoise	0.43000896
PLEK2	turquoise	0.20018282
PLEK	turquoise	0.89810844
PLEKHO1	turquoise	-0.1937555
PLEKHO2	turquoise	0.4072686
PLIN3	turquoise	-0.1839626
PNMA2	turquoise	-0.3333025
PNOC	turquoise	-0.209807
POU2F2	turquoise	0.03029851
PLPP3	turquoise	-0.3385722
PLPP7	turquoise	-0.3357083
PPBP	turquoise	-0.2168836
PPL	turquoise	-0.2418513
PPM1M	turquoise	0.07069427
PPP1R13L	turquoise	-0.2708328
PPP1R16B	turquoise	0.06671601
PPP1R9A	turquoise	-0.5671156
PRAM1	turquoise	0.19611255
PRELP	turquoise	-0.2344617
PREX2	turquoise	-0.3061341
PRKAR2A	turquoise	-0.4816528
PRKAR2B	turquoise	-0.4061256
PRKCB	turquoise	0.09345654
PRLR	turquoise	-0.4257002
PROM1	turquoise	-0.1952676
PRTG	turquoise	-0.6295371
PSMC1	turquoise	-0.4711617
PSMD11	turquoise	-0.5425492
PSMD1	turquoise	-0.6641817

PSMD2	turquoise	-0.4865828
PSMD3	turquoise	-0.4713388
PSMD5	turquoise	-0.3462829
PSMD7	turquoise	-0.3948297
PSME3	turquoise	-0.5303826
PSTPIP1	turquoise	0.2993229
PTAFR	turquoise	0.64171488
PTGFR	turquoise	-0.2229034
PTH1R	turquoise	-0.3778416
PTPN22	turquoise	0.75791549
PTPN7	turquoise	0.65872701
PTPRC	turquoise	0.92407367
PTPRM	turquoise	-0.2851295
PTPRO	turquoise	0.10766364
PVR	turquoise	-0.2707991
NECTIN2	turquoise	-0.4598401
NECTIN3	turquoise	-0.387777
RAB39B	turquoise	-0.3619866
RAB42	turquoise	0.08158697
RAD23B	turquoise	-0.4853365
RASAL3	turquoise	0.7803693
RASGRP4	turquoise	0.34581945
RASSF4	turquoise	0.33438253
RASSF5	turquoise	0.19637248
RBP5	turquoise	-0.3761083
RCSD1	turquoise	0.70376473
RELB	turquoise	0.01223157
RELN	turquoise	-0.2098122
RENBP	turquoise	0.32625809
REST	turquoise	-0.4672681
RFTN1	turquoise	0.13123099
RFXAP	turquoise	-0.5175459
RGS1	turquoise	0.65260458
RGS18	turquoise	0.68016066
RIF1	turquoise	-0.6238599
RIMKLA	turquoise	-0.4009374
RIN1	turquoise	0.05100613
RNASE2	turquoise	0.67046018
RNASE6	turquoise	0.85383117
RNF180	turquoise	-0.4833247
ROCK2	turquoise	-0.6428194
ROR1	turquoise	-0.4281805
RTKN2	turquoise	-0.5427016
RTN1	turquoise	0.31100173
RUNX1T1	turquoise	-0.2631692
RUNX3	turquoise	0.13748437
S100A12	turquoise	0.08621059
S100A7	turquoise	0.04950505
S100A7A	turquoise	-0.1165569
S100A8	turquoise	0.23450302
S100A9	turquoise	0.28747254

S100B	turquoise	0.11721058
SAMD14	turquoise	-0.3691343
SAMSN1	turquoise	0.88685643
SARDH	turquoise	-0.3582278
SASH3	turquoise	0.94837032
SBSN	turquoise	-0.3022303
SCEL	turquoise	-0.1670117
SCN7A	turquoise	-0.4224351
SCUBE3	turquoise	-0.4458458
SDC2	turquoise	-0.3855234
SDS	turquoise	0.5264058
SELPLG	turquoise	0.82706225
SERINC5	turquoise	-0.5580197
SERPINA1	turquoise	0.5608198
SERPING1	turquoise	-0.2342856
SFMBT2	turquoise	0.40220106
SGIP1	turquoise	-0.3882443
SH2D5	turquoise	-0.6234872
SHE	turquoise	-0.4283546
SIGLEC10	turquoise	0.76332169
SIGLEC11	turquoise	-0.2432017
SIGLEC14	turquoise	0.70476992
SIGLEC1	turquoise	0.68760716
SIGLEC7	turquoise	0.6734081
SIGLEC8	turquoise	0.46932355
SIGLEC9	turquoise	0.86604496
SIRPB1	turquoise	0.19936098
SIRPB2	turquoise	0.76699824
SLA	turquoise	0.8116951
SLAMF8	turquoise	0.84474178
SLC11A1	turquoise	0.40008999
SLC17A9	turquoise	-0.1168572
SLC1A7	turquoise	-0.5969743
SLC25A45	turquoise	-0.3189833
SLC2A5	turquoise	0.5342504
SLC34A2	turquoise	0.12373033
SLC45A3	turquoise	-0.544878
SLC7A7	turquoise	0.68117135
SLCO2B1	turquoise	0.73247551
SLCO5A1	turquoise	-0.5750341
SLIT2	turquoise	-0.399913
SLIT3	turquoise	-0.4272699
SNTB1	turquoise	-0.1510819
SNX20	turquoise	0.87399908
SOD3	turquoise	-0.1678913
SOX5	turquoise	-0.5341906
SPAG4	turquoise	-0.3746649
SPATA13	turquoise	-0.3379079
SPI1	turquoise	0.89640638
SPN	turquoise	0.54419162
SPOCK2	turquoise	-0.1905256

SPON1	turquoise	0.0038901
SPRY1	turquoise	-0.4500467
SRGN	turquoise	0.7436049
SSTR3	turquoise	-0.5377572
ST3GAL5	turquoise	-0.3323556
ST6GALNAC3	turquoise	-0.5776432
ST8SIA4	turquoise	0.19008968
STAB1	turquoise	0.67069779
STK17B	turquoise	0.22907672
STK33	turquoise	-0.6000129
SUCNR1	turquoise	0.19553306
SULT1C2	turquoise	-0.3798381
SYNE1	turquoise	-0.2610917
SYT11	turquoise	-0.3936913
TAGAP	turquoise	0.84359812
TBCEL	turquoise	-0.5689701
TBXAS1	turquoise	0.72591075
TCN2	turquoise	-0.076656
TESC	turquoise	-0.4319519
TFEC	turquoise	0.80926067
TGFB2	turquoise	-0.3736395
TGM1	turquoise	0.02507416
THPO	turquoise	-0.4385547
THSD7A	turquoise	-0.3929341
TIMD4	turquoise	0.31519147
TLR10	turquoise	0.27449606
TLR1	turquoise	0.56831074
TLR4	turquoise	0.67581452
TLR7	turquoise	0.80573882
TLR8	turquoise	0.69502551
TM6SF1	turquoise	0.49607831
TMC8	turquoise	0.5782014
TMEM106A	turquoise	0.21324262
TMEM150B	turquoise	0.67925199
TMEM176A	turquoise	0.29262288
TMEM176B	turquoise	0.39508323
TMEM233	turquoise	-0.5260783
TMEM26	turquoise	-0.3498549
TMEM47	turquoise	-0.3821588
TMEM79	turquoise	-0.3554201
SYNDIG1	turquoise	-0.2235753
TNF	turquoise	0.2103897
TNFAIP8L2	turquoise	0.88691752
TNFRSF10B	turquoise	-0.3413873
TNFRSF11A	turquoise	0.39630472
TNFRSF12A	turquoise	-0.300805
TNFRSF13C	turquoise	-0.4923579
TNFRSF1B	turquoise	0.63844084
TNFRSF25	turquoise	-0.2781356
TNFRSF4	turquoise	0.19070897
TNFSF13B	turquoise	0.73007222

TNFSF14	turquoise	0.13699614
TNFSF8	turquoise	0.32847897
TNN	turquoise	-0.2963539
TNNT2	turquoise	-0.402916
TOX	turquoise	-0.4480271
TRAF1	turquoise	0.10904203
TREM1	turquoise	0.32201752
TREM2	turquoise	0.71380336
TREML1	turquoise	0.15486003
TRPV2	turquoise	0.62278365
TRPV3	turquoise	-0.5418835
TSPAN32	turquoise	-0.3578704
TSPAN4	turquoise	-0.2610853
TTBK2	turquoise	-0.6082132
TTC37	turquoise	-0.4955147
TXK	turquoise	-0.3294208
TYMP	turquoise	0.73911173
TYROBP	turquoise	0.72588698
UHMK1	turquoise	-0.3450836
UNC5C	turquoise	-0.1831642
USHBP1	turquoise	-0.4690314
USP51	turquoise	-0.5452002
VAV1	turquoise	0.46303691
VEGFA	turquoise	-0.3971014
VEGFC	turquoise	-0.3611996
VENTX	turquoise	0.38153117
VMO1	turquoise	0.38286764
VNN2	turquoise	0.44522061
VSIG4	turquoise	0.78829197
WAS	turquoise	0.86268768
WDFY4	turquoise	0.65681966
WNT2	turquoise	-0.1357367
XPNPEP2	turquoise	-0.3059772
ZBTB10	turquoise	-0.4548329
ZDHHC20	turquoise	-0.3593545
ZNF185	turquoise	-0.1938182
ZNF366	turquoise	-0.4726803
ZNF521	turquoise	-0.3171852
ZNF620	turquoise	-0.6028224
ZNF660	turquoise	-0.7248016
ZNF671	turquoise	-0.3251494
ZNF835	turquoise	-0.3039902
EIF2A	turquoise	-0.4984241
EIF2AK3	turquoise	-0.6816578
EIF2AK1	turquoise	-0.6331272
HMGB1	turquoise	-0.5851488
P2RY2	turquoise	-0.1942114
ABCB1	blue	0.25681193
ABCD2	blue	0.91415715
ACAP1	blue	0.27030847
ACOXL	blue	0.65760277

ACSM5	blue	0.51555792
ADCYAP1	blue	0.29831238
ADORA2A	blue	0.91660244
ADRM1	blue	0.71539056
AKAP2	blue	0.78144237
AKAP5	blue	0.48202053
ALOXE3	blue	0.52466986
AMH	blue	0.0976372
AMIGO3	blue	0.96679887
AMPD1	blue	0.67937592
ANKRD55	blue	0.90614327
APOC2	blue	0.36740674
AQP10	blue	0.87441553
AREG	blue	0.13277265
ARHGAP31	blue	-0.6936122
ARHGEF6	blue	-0.7041414
ARL6IP5	blue	0.29545316
ARRDC5	blue	0.47098339
ART4	blue	0.91177243
ASAH1	blue	0.43395363
ASGR2	blue	0.50982067
ASRGL1	blue	0.32709099
ATP10A	blue	-0.2723642
ATP2A3	blue	-0.2499062
AZGP1	blue	0.41756838
BANK1	blue	0.11255878
BATF3	blue	0.26005989
BFSP2	blue	0.712389
BHLHE41	blue	0.1999645
BLK	blue	0.44236981
BTLA	blue	0.46696585
C11orf21	blue	0.33897971
SLIRP	blue	0.74897612
RTRAF	blue	0.68682025
TRIR	blue	0.73349969
MCEMP1	blue	0.21390976
C1orf127	blue	0.68752915
GCSAML	blue	0.59735127
PIK3CD-AS1	blue	0.79857479
C1orf54	blue	0.27509057
RTP5	blue	0.9350432
C4A	blue	-0.1757853
UQCC2	blue	0.68451242
CPED1	blue	-0.2037853
NUGGC	blue	0.17246837
C9orf139	blue	0.72988407
CAMK4	blue	0.47368219
CARD11	blue	-0.2002075
CASP5	blue	0.56616355
CCDC141	blue	0.95724038
CCDC69	blue	-0.1738403

CCL1	blue	0.74024922
CCL16	blue	0.95295724
CCL24	blue	0.09632338
CCR3	blue	0.57020602
CCR6	blue	0.97531135
CCR9	blue	0.90709318
CD160	blue	0.71054256
CD163L1	blue	0.28222378
CD19	blue	0.32970534
CD209	blue	-0.4210624
CD226	blue	0.74847496
CD302	blue	-0.1974144
CD93	blue	-0.6360574
CDSN	blue	0.64526228
CEACAM4	blue	0.36004244
CELF2	blue	-0.2550923
CFP	blue	0.4220199
CHAC1	blue	0.29841485
CHIT1	blue	0.20222275
CLEC4D	blue	0.6806136
CLEC4G	blue	0.51152339
CLEC4M	blue	0.45365106
CLEC6A	blue	0.63020653
CLEC9A	blue	0.59464826
CLNK	blue	0.86277322
CLTB	blue	0.58149279
CMA1	blue	0.49198831
CMKLR1	blue	-0.5162114
CNFN	blue	0.4687274
CNR2	blue	0.91450849
CNTF	blue	0.64629181
COL6A5	blue	0.48637022
CR1L	blue	0.58487315
CRLF2	blue	0.88095639
CSF2	blue	0.6520535
CSF3	blue	0.6338842
CTSB	blue	0.245392
CTSG	blue	0.33639249
CTSO	blue	-0.1721065
CXCR1	blue	0.34469342
CXCR2	blue	0.14202725
CXCR5	blue	0.96302616
CXorf65	blue	0.5302599
CYSLTR2	blue	0.3319548
DAB2	blue	-0.6531929
DHRS1	blue	0.59137181
DHRS9	blue	0.13495879
DNAH8	blue	0.9418584
DNAJC5B	blue	0.33187606
DOK1	blue	0.33519604
DOK5	blue	0.25721204

DSC1	blue	0.39938076
DSG1	blue	0.71371561
EGF	blue	0.64395725
ENPP3	blue	0.42018035
EPCAM	blue	0.60458576
EPO	blue	0.88061124
EPS8L1	blue	0.32775157
ERP27	blue	0.22060109
F13A1	blue	-0.6622973
F5	blue	0.35033852
FABP3	blue	0.4275234
DENND6B	blue	0.46187197
FAM177B	blue	0.54638916
CCSER1	blue	0.59108897
NXPE4	blue	0.89640269
FAM83A	blue	0.09900556
FAM92B	blue	0.40329374
FAS	blue	-0.138765
FCAR	blue	0.08692909
FCER2	blue	0.57588131
FCGR3B	blue	-0.0542762
FCRL1	blue	0.83536057
FCRL2	blue	0.82231739
FCRL3	blue	0.657217
FCRL5	blue	0.28191063
FCRLA	blue	0.4357832
FGD5	blue	-0.1390756
FGF14	blue	0.86697278
VEGFD	blue	0.45714666
FLT3	blue	0.55431495
FLT3LG	blue	0.308783
FPR2	blue	0.21098124
GATA1	blue	0.81925589
GGT1	blue	0.19343976
GHRL	blue	0.38457282
GJA5	blue	-0.2165926
GJB5	blue	0.23613188
GLIPR2	blue	0.13000333
GMPR	blue	0.44152349
GNAI2	blue	0.39170543
GPC5	blue	0.45532306
ADGRF4	blue	0.28097496
GPR137B	blue	-0.1064039
GPR15	blue	0.66334957
GPR157	blue	0.19002993
GPR55	blue	0.68598208
GPR78	blue	0.74094094
GRAP	blue	0.47191882
GRIN3A	blue	0.84582782
HAVCR1	blue	0.78195906
HEPHL1	blue	0.66653784

HERPUD1	blue	0.20045224
HIST1H2AE	blue	0.50466133
HIST1H2AM	blue	0.42177809
HIST1H3H	blue	0.31930138
HSD17B14	blue	0.31202759
HSP90AA1	blue	0.55108599
HSPA1A	blue	0.19098027
HSPA1B	blue	0.57629283
HTR2A	blue	0.83197592
HTRA4	blue	0.35708665
ICAM1	blue	-0.3747396
ICOSLG	blue	0.47473375
IDO2	blue	0.83894408
IFFO1	blue	-0.6127856
IFITM3	blue	0.61797032
IFNB1	blue	0.67828287
IFNE	blue	0.53721819
IFNGR2	blue	0.49825751
IGLL1	blue	0.29381171
IL10	blue	-0.3785404
IL10RB	blue	0.41064707
IL12A	blue	0.20130308
IL12B	blue	0.93007541
IL12RB2	blue	0.43813267
IL13	blue	0.97191492
IL17B	blue	0.52369047
IL19	blue	0.63015139
IL1A	blue	0.3242017
IL36G	blue	0.86915479
IL1R2	blue	0.36198205
IL1RL1	blue	0.58675204
IL20	blue	0.57638636
IL20RA	blue	0.108724
IL20RB	blue	0.45578996
IL22RA2	blue	0.41337617
IL23R	blue	0.95604051
IL24	blue	0.13639197
IL27	blue	0.61505717
IFNL2	blue	0.6106029
IL4	blue	0.87152432
IL4R	blue	-0.4323847
IL5	blue	0.93407277
IL5RA	blue	0.48258218
IL7	blue	0.18006302
IL7R	blue	-0.624658
IL9R	blue	0.67179733
INHBC	blue	0.65893447
IPCEF1	blue	0.40004259
ITGA2B	blue	0.57230906
ITGA9	blue	-0.1262283
ITGAD	blue	0.5665103

ITGB3	blue	-0.1530029
JAK3	blue	-0.1797427
JAKMIP1	blue	0.51422838
JSRP1	blue	0.1420156
KCNJ10	blue	0.31964689
FAM30A	blue	0.50340629
KIR2DL1	blue	0.84433057
KIR2DL3	blue	0.6199561
KIR2DS4	blue	0.62450619
KIR3DL1	blue	0.90729653
KIR3DL2	blue	0.90729738
KLRC2	blue	0.74856163
KLRC4	blue	0.75436603
KLRD1	blue	0.58171221
KLRK1	blue	0.84538104
KRT1	blue	0.74089199
KRT6A	blue	-0.0973368
KRT6C	blue	0.71729002
KRT78	blue	0.63360244
LAD1	blue	0.46571235
LAIR2	blue	0.52216483
LAT	blue	0.75034891
LCN10	blue	0.81727359
LIFR	blue	0.31600907
LILRP2	blue	0.90466494
UNQ6494	blue	0.86237516
FAM83A-AS1	blue	0.33937063
SMIM1	blue	0.53711983
LPL	blue	-0.1489557
NRROS	blue	-0.3151388
LTBR	blue	0.50223085
LTC4S	blue	0.59062881
MAN1A1	blue	-0.4576674
MAP4K1	blue	0.29951715
MEF2B	blue	0.52408432
MIR155HG	blue	0.21217334
MMP25	blue	0.14846499
MRC1	blue	-0.548342
MRO	blue	0.30776723
MRPL27	blue	0.68110253
MRPL55	blue	0.7927612
MRPS12	blue	0.56600333
MRPS21	blue	0.73371562
MS4A2	blue	0.77534559
N4BP2L1	blue	0.29452096
NAIP	blue	0.78880713
NBEAL1	blue	0.76545818
NCR1	blue	0.66094753
NFKB2	blue	0.31563393
NFKBID	blue	0.22698121
NFYC	blue	0.4894332

NIPAL4	blue	0.64710524
NLRC3	blue	0.17777247
NLRP12	blue	0.695446
NRP1	blue	-0.3116501
OTOA	blue	0.87952046
P2RX4	blue	0.38220118
P2RX5	blue	0.47548113
P2RY11	blue	0.36169965
P2RY14	blue	0.16747669
PAG1	blue	-0.1577062
PAK5	blue	0.53309989
PALM2-AKAI	blue	0.85988956
PARM1	blue	-0.1946701
PECAM1	blue	-0.6036908
PFDN2	blue	0.83575769
PGLYRP4	blue	0.41737685
PKD2L1	blue	0.70310818
PLA2G4E	blue	0.95448505
PLA2G7	blue	-0.1413754
PLEKHN1	blue	0.16671226
PLXNC1	blue	-0.4919948
PLXND1	blue	-0.3600016
PPFIA2	blue	0.81140329
PRG2	blue	0.88605796
PRL	blue	0.55364405
PRSS27	blue	0.49954285
PSMC2	blue	0.66559448
PSMC3	blue	0.69349312
PSMC4	blue	0.52027092
PSMC5	blue	0.55687789
PSMC6	blue	0.56266131
PSMD10	blue	0.56245588
PSMD13	blue	0.62546835
PSMD14	blue	0.60619557
PSMD4	blue	0.76399601
PSMD6	blue	0.62755775
PSMD8	blue	0.51839059
PSME1	blue	0.62440698
PTCRA	blue	0.58006418
PTGDR	blue	0.55130875
PTGER2	blue	-0.2671463
PUS10	blue	0.60379283
QPRT	blue	0.49087543
RAB20	blue	0.28764366
RAB33A	blue	0.26295842
RAB37	blue	0.44678208
RAB39A	blue	0.15126322
RAET1E	blue	0.75822057
RAET1G	blue	0.57593068
RAET1L	blue	0.59865899
RAI2	blue	0.31108922

RAPGEF6	blue	0.68497713
RASGRP2	blue	0.22455072
RASSF6	blue	0.4617764
RBM38	blue	0.38656906
RDH12	blue	0.71941659
RFXANK	blue	0.6900782
RGL4	blue	0.62175287
RGPD1	blue	0.95767731
RGS13	blue	0.96016538
RHOD	blue	0.54406073
RIN3	blue	-0.2401116
CARMIL2	blue	0.16754327
RNASE1	blue	0.17492125
RNASE7	blue	0.65536842
RNF222	blue	0.87861377
LAMTOR2	blue	0.75498352
RPS6KA4	blue	0.24268253
RRN3P2	blue	0.51528774
S100A16	blue	0.3395662
S100A2	blue	0.18240427
SAMD3	blue	0.69778514
SCARF1	blue	-0.2470672
SCML4	blue	0.57301538
SDR9C7	blue	0.66666439
SDSL	blue	0.50840867
SFN	blue	0.23036405
SH2B3	blue	-0.6627672
SEM1	blue	0.75812674
SIGLEC12	blue	0.19221831
SIGLEC6	blue	0.59526506
SIGLEC17P	blue	0.40574283
SLC12A3	blue	0.60800053
SLC18A2	blue	0.68374809
SLC24A4	blue	0.94951745
SLC29A3	blue	0.25662451
SLC39A2	blue	0.44149339
SLC6A12	blue	0.10877281
SLURP1	blue	0.12187588
SMPDL3B	blue	0.47759087
SNRPF	blue	0.74746495
SOX17	blue	0.5554892
SPIB	blue	0.26212148
ST3GAL6	blue	0.23715516
STXBP6	blue	0.1928581
SULT2B1	blue	0.25936637
SVOPL	blue	0.33474254
TARP	blue	0.76780324
TCL1A	blue	0.26119584
TGFB1	blue	-0.5643974
TGFBI	blue	-0.6709397
TIFAB	blue	0.57445479

PAM16	blue	0.6506301
TIMM50	blue	0.50953312
TLR9	blue	0.82118249
DCSTAMP	blue	0.37261755
TNFRSF10A	blue	0.3156067
TNFRSF10C	blue	0.18386252
TNFRSF13B	blue	0.85855485
TNFRSF1A	blue	0.41677859
TNFRSF8	blue	-0.109263
TNFSF11	blue	0.38567924
TNFSF12-TN	blue	0.94450973
TNFSF18	blue	0.44555592
TNIP3	blue	0.23395216
TPSAB1	blue	-0.230133
TPSB2	blue	-0.1984529
TPSD1	blue	-0.0340901
TPSG1	blue	0.74996875
TRIM61	blue	0.26723565
TSHR	blue	0.37509194
TSLP	blue	0.7258334
TTC16	blue	0.51172609
TTC24	blue	0.63108064
TUBA4A	blue	0.11684176
NME8	blue	0.32024041
UBXN11	blue	0.60379623
UBXN1	blue	0.75178458
UCP2	blue	0.49336226
ULBP1	blue	0.52232435
ULBP2	blue	0.13509587
UTS2	blue	0.49787546
VASH1	blue	-0.3596097
VEGFB	blue	0.68488669
VPREB3	blue	0.37490771
VPS37D	blue	0.52306238
WFDC12	blue	0.48378914
WIPF1	blue	-0.6943388
XCL1	blue	0.22150241
XKR8	blue	0.42671624
ZBTB32	blue	0.41941777
ZEB2	blue	-0.7483445
ZNF215	blue	0.45187603
ZNF804A	blue	0.60623812
ZNF80	blue	0.9007608
ZNF831	blue	0.67375031
ANXA1	blue	0.17596036
HHLA2	blue	0.60000451
ABCC9	brown	0.270832
ACTA2	brown	0.78930617
ACVRL1	brown	0.4274302
ADAM12	brown	0.84876757
ADAM28	brown	-0.2163508

ADAMTS10	brown	0.54796043
ADAMTS12	brown	0.86375213
ADAMTS14	brown	0.52807524
ADAMTS16	brown	0.46664095
ADAMTS2	brown	0.84794081
ADAMTS4	brown	0.66315318
ADAMTSL2	brown	0.48686565
ADRA2A	brown	0.41725077
AEBP1	brown	0.89732071
AGTR1	brown	0.47300959
ALDH3B1	brown	-0.3621776
ALPK2	brown	0.53813184
ANGPTL2	brown	0.82518762
SOWAHD	brown	-0.4063079
ANTXR1	brown	0.81590365
ANTXR2	brown	0.8263938
AOC3	brown	0.5964773
APLNR	brown	0.64810561
AQP1	brown	0.58387272
ARHGAP25	brown	-0.2546038
ARHGAP4	brown	-0.2678725
ARRB2	brown	-0.3445213
ARSB	brown	0.4298914
CLMP	brown	0.84416283
ASPN	brown	0.7632785
AVPR1A	brown	0.48545339
BCL2L14	brown	-0.551812
BGN	brown	0.77028171
BHLHE22	brown	0.47255487
BICC1	brown	0.72265845
C10orf99	brown	-0.2554111
MEDAG	brown	0.61069166
C19orf38	brown	-0.3502848
C1QTNF7	brown	0.51222847
C5orf56	brown	-0.5032437
ADTRP	brown	-0.2655252
CALB2	brown	0.51284098
CALD1	brown	0.6886066
CARD9	brown	-0.3185672
CCDC80	brown	0.75944583
CCL11	brown	0.50996574
CCL20	brown	-0.2498467
CCL21	brown	0.40316095
CCL23	brown	-0.2927276
CCL25	brown	-0.3237277
CCL26	brown	0.35467589
CCL28	brown	-0.373259
CCR8	brown	-0.1647768
CD1D	brown	-0.3587783
CD200R1	brown	-0.2719042
CD248	brown	0.71000143

CD36	brown	0.46913642
CD40	brown	-0.2413404
CD58	brown	-0.2790389
ADGRE5	brown	-0.2573786
CDH11	brown	0.8810001
CHN1	brown	0.3362517
CHRD	brown	0.4627933
CHRNA6	brown	-0.4684106
CILP	brown	0.53396907
CISH	brown	-0.1997472
CLECL1	brown	-0.4905942
CMAHP	brown	0.34900306
CNRIP1	brown	0.71360265
COL10A1	brown	0.7318787
COL11A1	brown	0.81328566
COL12A1	brown	0.63879466
COL15A1	brown	0.5760824
COL1A1	brown	0.89688065
COL1A2	brown	0.87126193
COL3A1	brown	0.91674539
COL4A1	brown	0.64227071
COL4A2	brown	0.50259754
COL5A1	brown	0.92788645
COL5A2	brown	0.92077343
COL5A3	brown	0.71203989
COL6A1	brown	0.74385294
COL6A2	brown	0.82415822
COL6A3	brown	0.89151057
COL6A6	brown	0.42819198
COL8A1	brown	0.83194244
COLEC12	brown	0.74435065
CORIN	brown	0.44168968
CPA3	brown	0.42211707
CREB3L1	brown	0.74406593
CRISPLD2	brown	0.84323372
CSMD2	brown	0.32140272
CTHRC1	brown	0.71772662
CTSK	brown	0.79379652
CXCL12	brown	0.70020664
CXCL16	brown	-0.3704335
CYBA	brown	-0.4791667
CYP1B1	brown	0.37556038
DACT1	brown	0.78214843
DACT3	brown	0.6582972
DCHS1	brown	0.62280791
DCN	brown	0.82203012
DDR2	brown	0.52766372
DKK2	brown	0.46600772
DOCK11	brown	0.32303012
DPT	brown	0.64498271
ECM2	brown	0.77029068

EDNRA	brown	0.73057295
EHD2	brown	0.54398282
EMILIN1	brown	0.81472109
ADGRE4P	brown	-0.4324665
ENOX1	brown	0.54697685
ENPP2	brown	-0.1045258
ETS1	brown	0.48635326
F2R	brown	0.59214224
FAM107A	brown	-0.2961825
FAM155A	brown	0.40432987
CALHM5	brown	0.46683043
PIEZO2	brown	0.46262065
RIPOR2	brown	-0.245507
FAP	brown	0.83491893
FBLN2	brown	0.49803505
FBN1	brown	0.94530645
FBXL7	brown	0.46162683
FBXO6	brown	-0.4531439
FGF7	brown	0.71187393
FIBIN	brown	0.5832934
FILIP1L	brown	0.79893393
FN1	brown	0.82843663
FNDC1	brown	0.71397251
FSTL1	brown	0.6711996
FSTL3	brown	0.52490019
FUCA1	brown	-0.3495187
FUT7	brown	-0.4734375
GALM	brown	-0.4830733
GALNT15	brown	0.41245558
GAS7	brown	0.45497002
GCSAM	brown	-0.419189
GGT5	brown	0.42142294
GIMAP5	brown	-0.3506122
GJB2	brown	0.56854648
GLT8D2	brown	0.82551443
GMIP	brown	-0.2594395
GNG2	brown	0.50947874
GPBAR1	brown	-0.368606
ADGRG5	brown	-0.394233
ADGRA2	brown	0.72537733
GPR4	brown	0.56082111
GREM1	brown	0.59327587
GVINP1	brown	-0.4471792
HEPH	brown	0.76219045
HIC1	brown	0.81323135
HLA-DPB2	brown	-0.253097
HLX	brown	0.36010268
HMCN1	brown	0.54264229
HRH2	brown	0.31938536
HSD11B1	brown	-0.0239995
HTRA3	brown	0.71171923

HVCN1	brown	-0.1117356
IFNAR2	brown	-0.244994
IFNGR1	brown	-0.2464482
IL6	brown	0.26855378
IL6R	brown	-0.3406308
INHBA	brown	0.80202274
INMT	brown	0.24655057
ISLR	brown	0.5912195
ITGA11	brown	0.86473993
ITGA5	brown	0.75276841
ITGBL1	brown	0.63846936
JAM2	brown	0.36236189
JAM3	brown	0.55756884
KCNE4	brown	0.85021681
KCNJ8	brown	0.31919207
KLK7	brown	-0.2538443
KMO	brown	-0.3867482
LAMA4	brown	0.63933918
LDB2	brown	0.55913841
LHFPL6	brown	0.77680577
LIF	brown	0.27877815
LILRA4	brown	-0.1048501
LMOD1	brown	0.69078989
LINC00426	brown	-0.4727126
LOXL2	brown	0.78197336
LPAR4	brown	0.47215377
PLPPR4	brown	0.62994913
LRMP	brown	-0.2901793
LRRC15	brown	0.7733172
LSAMP	brown	0.40219057
LTBP2	brown	0.49502076
LUM	brown	0.82244195
LYPD5	brown	-0.2922523
MCOLN2	brown	-0.3100572
MEOX2	brown	0.37204373
MFAP4	brown	0.59103388
MICAL2	brown	0.42572503
MLPH	brown	-0.342221
MMP1	brown	0.24588992
MMP14	brown	0.78497893
MMP2	brown	0.88477237
MMRN2	brown	-0.1695195
MRGPRF	brown	0.57424231
MRVI1	brown	0.79092587
MS4A1	brown	-0.0932701
MSRB3	brown	0.78335396
MXRA8	brown	0.55826667
MYO7A	brown	-0.303503
NFKBIE	brown	-0.2962999
NID2	brown	0.66337967
NOX4	brown	0.16851148

NR1H3	brown	-0.4806228
NT5E	brown	0.56693668
NTM	brown	0.73069551
TENM3	brown	0.50286513
OGN	brown	0.53096704
OLFML1	brown	0.74828179
OLFML2B	brown	0.78740324
OLFML3	brown	0.56178837
OMD	brown	0.7083434
P4HA3	brown	0.66300961
PATL2	brown	-0.5399771
PCDHGA12	brown	0.57246855
PCOLCE	brown	0.70312431
PDE6G	brown	-0.1638174
PDGFB	brown	0.31760662
PDGFRA	brown	0.72604042
PDGFRB	brown	0.87194629
PDGFRL	brown	0.35119274
PDZRN3	brown	0.52015709
PKP3	brown	-0.3809774
PLVAP	brown	0.47852232
PLXDC1	brown	0.76239782
PML	brown	-0.3210951
PMP22	brown	0.69353096
PODN	brown	0.77917356
POSTN	brown	0.75742352
PLPP4	brown	0.19861642
PRKCQ	brown	-0.4145637
PRKG1	brown	0.69755446
PROCR	brown	0.27798293
PRRX1	brown	0.81433695
PSMB10	brown	-0.5200978
PSME2	brown	-0.5726864
PTGIR	brown	0.56370654
PTGIS	brown	0.75264858
PTPN6	brown	-0.5009003
CAVIN1	brown	0.63392757
RASGRF2	brown	0.59485144
RASSF2	brown	0.37418642
RCAN2	brown	0.57765618
RCN3	brown	0.63483202
RECK	brown	0.5332758
RFX5	brown	-0.4981656
RNF125	brown	-0.2889207
RNF166	brown	-0.2661211
SCARF2	brown	0.62018602
SDCBP2	brown	-0.3367622
SEC24D	brown	0.33963762
SELENBP1	brown	-0.5610433
SELP	brown	0.26362318
SERPINE1	brown	0.72079897

SERPINF1	brown	0.79458009
SFRP2	brown	0.7142605
SFTPB	brown	-0.2916716
SGCD	brown	0.36300595
SH2D2A	brown	-0.4208188
SIGLEC5	brown	-0.3748867
SKAP1	brown	-0.4926714
SNAI3	brown	-0.338578
SPARC	brown	0.89006156
SPARCL1	brown	0.41333452
SPNS3	brown	-0.4196538
SSC5D	brown	0.72798412
STAC3	brown	-0.3303193
STARD8	brown	0.44870567
STAT5A	brown	-0.2482842
SULF1	brown	0.54385308
SUSD3	brown	-0.3879687
SYTL3	brown	-0.4319768
TAPBP	brown	-0.4233867
TAPBPL	brown	-0.4612988
TBC1D10C	brown	-0.3116409
TCEAL7	brown	0.53989598
TCIRG1	brown	-0.3456188
TGFB3	brown	0.59052147
TGFBR2	brown	0.40183578
TGM2	brown	0.27645824
THBS1	brown	0.77430863
THBS2	brown	0.86652275
THY1	brown	0.33529721
TIMP2	brown	0.69421344
TIMP3	brown	0.74095307
TLR5	brown	-0.2015223
TMEM119	brown	0.45478752
IGFLR1	brown	-0.3157729
TMEM200A	brown	0.74119741
TMEM204	brown	0.55316932
TNFAIP6	brown	0.54828132
TNFRSF14	brown	-0.4412713
TNFSF13	brown	-0.4095948
TNFSF15	brown	-0.3532022
TNFSF4	brown	0.59796271
TPK1	brown	-0.2486753
TSHZ3	brown	0.55454336
TUBB6	brown	0.31877307
UNC13D	brown	-0.1727062
UNC93B1	brown	-0.5070469
VCAM1	brown	0.68945338
VCAN	brown	0.8818876
VGLL3	brown	0.7785708
VIM	brown	0.39969669
XCR1	brown	-0.3928862

ZC3H12D	brown	-0.3011444
ZCCHC24	brown	0.69300498
ZEB1	brown	0.77459317
ZMYND15	brown	-0.4372969
ZNF469	brown	0.77898504
LRP1	brown	0.57556696
ACSL5	green	0.38349784
APOBEC3A	green	0.594213
APOBEC3D	green	0.43301329
APOBEC3G	green	0.57134871
APOL6	green	0.69855915
B2M	green	0.62971099
BATF2	green	0.72053604
BST2	green	0.53127731
BTN3A1	green	0.41357929
BTN3A2	green	0.49956986
BTN3A3	green	0.57443387
RUBCNL	green	-0.3023518
CAV1	green	-0.3522972
CCL7	green	0.39062993
CCL8	green	0.55756667
CD274	green	0.67766605
CD80	green	0.48811563
CMPK2	green	0.85412442
CPVL	green	-0.2280621
CTSZ	green	-0.1288961
CXCL10	green	0.73893631
CXCL11	green	0.76178938
DDX58	green	0.78376356
DDX60	green	0.83118924
DOCK4	green	-0.3690589
EDA2R	green	-0.3826913
EPSTI1	green	0.6948398
ERAP2	green	0.16152844
ETV7	green	0.85519059
GBGT1	green	-0.2458831
GBP1	green	0.69943743
GBP4	green	0.72695062
GNG7	green	-0.2244893
GPC6	green	-0.5287142
HCP5	green	0.69231255
HERC6	green	0.82002581
HGF	green	-0.2211899
HLA-A	green	0.61613474
HLA-B	green	0.68370862
HLA-C	green	0.62363474
HLA-DOB	green	0.51773708
HLA-E	green	0.60947421
HLA-F	green	0.69686506
HLA-G	green	0.45570928
HSH2D	green	0.63652017

IFI27	green	0.73987223
IFI35	green	0.81191858
IFI44	green	0.82695863
IFI44L	green	0.84085348
IFI6	green	0.67196072
IFIH1	green	0.89154659
IFIT2	green	0.79568907
IFIT3	green	0.87648801
IFIT5	green	0.59963446
IFITM1	green	0.54887092
IFNL1	green	0.57064356
IL4I1	green	0.46161516
IRF7	green	0.53884733
IRF9	green	0.4271165
ISG15	green	0.78817469
ISG20	green	0.60721306
JAK2	green	0.48004931
SHISAL1	green	-0.4075719
LAP3	green	0.61917419
LGALS9	green	0.62445114
LGALS17A	green	0.6950044
LRRC32	green	-0.3604596
MGP	green	-0.2491606
MICB	green	0.46792442
MX1	green	0.87196508
NLRC5	green	0.6343708
NMI	green	0.71879802
NOD2	green	0.49455476
NOVA2	green	-0.2789153
OAS1	green	0.88025757
OAS2	green	0.87308882
OAS3	green	0.82681688
OASL	green	0.87168526
PARP12	green	0.77489357
PARP14	green	0.81723516
PARP9	green	0.7990077
PSMB8	green	0.68463877
PSMB9	green	0.7574562
RARRES2	green	-0.3555541
RASGRP3	green	0.28049122
RSAD2	green	0.85113165
RTP4	green	0.72971706
RUFY4	green	0.54690788
SAMD9	green	0.81225454
SAMD9L	green	0.8089297
SAMHD1	green	0.44232379
SECTM1	green	0.51668936
SLC15A3	green	0.66926861
SP100	green	0.7328724
SP110	green	0.73762691
STAP1	green	0.573877

STAT1	green	0.74030671
TAP1	green	0.82597875
TAP2	green	0.73862481
TMEM140	green	0.4953607
TMEM229B	green	0.45483271
TNFRSF10D	green	-0.4834716
TNFSF10	green	0.57462408
TRANK1	green	0.50489691
TRIM21	green	0.67907601
TRIM22	green	0.71869875
UBA7	green	0.5258509
UBE2L6	green	0.74745197
WARS	green	0.49455385
XAF1	green	0.75490408
ZBP1	green	0.8208009
ZFPM2	green	-0.2824553
EIF2AK2	green	0.4984417
TLR3	green	0.61017773
VTCN1	green	0.36618089
ACVR1B	red	0.72059413
ACVR2B	red	0.75032264
ADPRH	red	0.3693569
AHCYL2	red	0.68260441
ANKRD17	red	0.66358427
ANO6	red	0.60734796
ASXL2	red	0.85487689
BCL2A1	red	-0.6363487
BHLHA15	red	0.24727156
BIRC6	red	0.84335788
BMP2K	red	0.37601425
ERCC6L2	red	0.6818457
CAMK1	red	0.33451427
CARD8	red	0.55248343
CCL2	red	-0.5292612
CCNT1	red	0.80422591
CD70	red	-0.2626799
CDKL5	red	0.59724178
CREB1	red	0.79229877
CREBL2	red	0.4372662
CXCL2	red	-0.1915548
ACKR1	red	-0.3434913
DDI2	red	0.65599475
DMXL2	red	0.56979285
DSP	red	0.53160477
DUSP16	red	0.65808352
EFTUD2	red	0.61122062
EGFR	red	0.29334949
ENTPD1	red	0.32313741
EP300	red	0.717258
ERAP1	red	0.29889774
ERN1	red	0.55004948

ETV3	red	0.73653853
FAM168A	red	0.71339704
FMNL3	red	0.47256118
FNBP1	red	0.34578715
FNIP2	red	0.38395615
GIT2	red	0.69141286
GLRX	red	-0.5501165
GNS	red	0.63215792
GYPC	red	-0.3232737
HCST	red	-0.7075529
HSPA8	red	0.43396971
ICK	red	0.53311199
IL13RA1	red	0.1974001
IL1R1	red	0.23920308
IL32	red	-0.4265476
KIAA0754	red	0.53533157
KIAA1549	red	0.74449761
KLHDC10	red	0.62674526
KLHL11	red	0.72097737
LATS1	red	0.70553279
LGMN	red	0.24957106
LIG3	red	0.6815575
LMTK2	red	0.81611746
LRP6	red	0.73444901
LY96	red	-0.7436419
MAP3K2	red	0.58358684
MED13	red	0.70192373
MED13L	red	0.7381712
MFAP3	red	0.57429555
MGAT4A	red	0.28153492
MGAT5	red	0.61162979
MR1	red	0.23199699
MXD1	red	0.61080592
NCOA2	red	0.60583754
NFATC2	red	0.43049026
NFYA	red	0.71805371
NHSL2	red	0.5654311
PIP4K2A	red	0.24019623
PLCL2	red	0.25944559
PLEKHM3	red	0.81293383
PPM1H	red	0.54608054
PREX1	red	0.40143749
PSAP	red	0.33685021
PTGDS	red	-0.154944
PTPRJ	red	0.40984993
PVRIG	red	0.59584818
RAB8B	red	0.23651362
RAD54L2	red	0.77959656
RAPGEF2	red	0.71717435
RASSF3	red	0.59106456
RC3H2	red	0.63037066

REL	red	0.65266217
RGL1	red	0.27777936
SBNO1	red	0.78418514
SEC24A	red	0.51819817
SECISBP2L	red	0.61827464
POMK	red	0.68047655
PEAK1	red	0.5885652
SLC8A1	red	0.31785607
SMAP2	red	0.25630147
SON	red	0.7843194
ST6GAL1	red	0.33844449
STRN	red	0.69051082
TAOK1	red	0.78145307
TGFBRAP1	red	0.79523751
TMEM170B	red	0.6148082
TNFRSF11B	red	-0.1702392
TNFSF12	red	-0.3501355
TNFSF9	red	-0.1735318
TNIK	red	0.24439948
TNS3	red	0.3633027
TRPC4AP	red	0.60784689
TTC21B	red	0.73344479
UBR1	red	0.71256356
ULBP3	red	0.5768301
USP12	red	0.47944829
VAMP5	red	-0.6456695
ZKSCAN1	red	0.7405033
ZKSCAN8	red	0.73752117
ZNF827	red	0.64744768
EIF2AK4	red	0.71509326
PANX1	red	0.49365277
ADAMTS9	yellow	-0.502397
AIM2	yellow	0.61253597
ANKRD22	yellow	0.67424649
ANXA6	yellow	-0.4920083
APBB2	yellow	-0.5048219
APOBEC3H	yellow	0.53925558
APOL3	yellow	0.60007446
BATF	yellow	0.61801081
BDKRB2	yellow	-0.2872673
VSTM4	yellow	-0.5557949
C16orf54	yellow	0.55971471
LAMP5	yellow	-0.4298545
C2	yellow	0.3194404
NDNF	yellow	-0.4736896
CCL13	yellow	0.38848065
CCL19	yellow	0.45825647
CCL22	yellow	0.37833091
CCL5	yellow	0.86988946
CCR2	yellow	0.61384256
CCR4	yellow	0.54851117

CCR7	yellow	0.5529366
CD244	yellow	0.59474081
CD247	yellow	0.87739557
CD27	yellow	0.72461218
CD2	yellow	0.90162692
CD3D	yellow	0.90746652
CD3E	yellow	0.90706991
CD3G	yellow	0.76143027
CD40LG	yellow	0.69411048
CD52	yellow	0.66008189
CD5	yellow	0.76454568
CD6	yellow	0.38870544
CD72	yellow	0.43281583
CD74	yellow	0.56791474
CD7	yellow	0.77130685
CD79A	yellow	0.51418295
CD79B	yellow	0.26940209
CD8A	yellow	0.78901781
CD8B	yellow	0.39429126
CD96	yellow	0.86036551
CIITA	yellow	0.41205588
CLEC12A	yellow	0.2647828
CLIC5	yellow	-0.1923105
COL14A1	yellow	-0.479958
CPZ	yellow	-0.4541453
CST7	yellow	0.6045593
CTLA4	yellow	0.72977749
CTSW	yellow	0.76674699
CXCL13	yellow	0.69050535
CXCL9	yellow	0.75986181
CXCR3	yellow	0.88155408
CXCR6	yellow	0.88777303
ACKR3	yellow	-0.4218388
CYSLTR1	yellow	0.3062595
DAAM2	yellow	-0.3499749
DENND2A	yellow	-0.5903111
DERL3	yellow	0.24665139
ENG	yellow	-0.4391802
EOMES	yellow	0.66484348
EPS8	yellow	-0.2666751
FASLG	yellow	0.83172974
FAT4	yellow	-0.5122693
FLT4	yellow	-0.3563861
FMOD	yellow	-0.4851002
FOXP3	yellow	0.63676011
FSCN1	yellow	-0.4921486
GATA3	yellow	0.17594648
GBP2	yellow	0.53803448
GBP5	yellow	0.70579712
GFI1	yellow	0.5631905
GLIS3	yellow	-0.4015429

GNLY	yellow	0.62270379
GPR171	yellow	0.83034823
GPR174	yellow	0.46068746
GPR18	yellow	0.5272549
GPR25	yellow	0.43239572
GRAP2	yellow	0.61742038
GZMA	yellow	0.87203625
GZMB	yellow	0.7612634
GZMH	yellow	0.84275905
GZMK	yellow	0.75342208
GZMM	yellow	0.79697305
HECW2	yellow	-0.3981668
HLA-DMA	yellow	0.55309948
HLA-DMB	yellow	0.45292944
HLA-DOA	yellow	0.44060098
HLA-DPA1	yellow	0.63198351
HLA-DPB1	yellow	0.66126751
HLA-DQB1	yellow	0.52994978
HLA-DQB2	yellow	0.46901188
HLA-DRA	yellow	0.60309684
HLA-DRB1	yellow	0.60008907
HLA-DRB5	yellow	0.47106018
HLA-DRB6	yellow	0.37718584
HS3ST1	yellow	-0.2175017
ICAM3	yellow	0.61129644
ICOS	yellow	0.79716146
IDO1	yellow	0.54783135
IFNG	yellow	0.61903336
IGDCC4	yellow	-0.6315004
JCHAIN	yellow	0.61349217
IKZF3	yellow	0.60431939
IL15	yellow	0.48681675
IL15RA	yellow	0.53373796
IL18BP	yellow	0.46774482
IL18RAP	yellow	0.49854054
IL21R	yellow	0.75523053
IL2RB	yellow	0.78442719
IL2RG	yellow	0.86094571
IRF1	yellow	0.50228353
IRF4	yellow	0.56866316
ITK	yellow	0.83833097
KCNA3	yellow	0.34733682
TESPA1	yellow	0.71052212
JCAD	yellow	-0.3307344
KIR2DL4	yellow	0.51272852
KIRREL1	yellow	-0.5679976
KLRB1	yellow	0.77327064
KLRC1	yellow	0.43535731
LAG3	yellow	0.51690023
LAX1	yellow	0.51127986
LCK	yellow	0.64033889

LGALS2	yellow	0.27839603
LOXL3	yellow	-0.422883
LTA	yellow	0.71912183
LTB	yellow	0.42633916
LY9	yellow	0.58716261
MAN1C1	yellow	-0.3020526
MEI1	yellow	0.31237534
MZB1	yellow	0.56050619
NCR3	yellow	0.64274001
NKG7	yellow	0.86114803
P2RX1	yellow	0.09065557
P2RY10	yellow	0.8012964
P2RY8	yellow	0.49073031
PARP15	yellow	0.44838416
PDCD1	yellow	0.59640507
PIM2	yellow	0.2788375
PLA2G2D	yellow	0.62305267
PLXNA4	yellow	-0.4620025
POU2AF1	yellow	0.28081217
PRF1	yellow	0.83589264
PYHIN1	yellow	0.76113282
RASL12	yellow	-0.4798843
RHOH	yellow	0.79424017
S1PR4	yellow	0.70135247
SALL2	yellow	-0.5401947
SELL	yellow	0.56255004
SH2D1A	yellow	0.845612
SIRPG	yellow	0.89320638
SIT1	yellow	0.81433741
SLA2	yellow	0.86391722
SLAMF1	yellow	0.83535924
SLAMF6	yellow	0.84744974
SLAMF7	yellow	0.72380111
SNED1	yellow	-0.4295892
SP140	yellow	0.64627563
ST3GAL2	yellow	-0.483985
STARD13	yellow	-0.2911793
STAT4	yellow	0.48679312
SULT1C4	yellow	-0.4517992
TBX21	yellow	0.79626741
TGFBR1	yellow	-0.4485421
THEMIS	yellow	0.73966618
TIGIT	yellow	0.79543657
TMEM156	yellow	0.56670678
TMIGD2	yellow	0.59344041
TNFRSF17	yellow	0.45993624
TNFRSF18	yellow	0.32190642
TNFRSF9	yellow	0.43794589
TRAF3IP3	yellow	0.70691067
TRAT1	yellow	0.75894366
TSPAN11	yellow	-0.4281914

UBASH3A	yellow	0.71773498
UBD	yellow	0.62782228
XCL2	yellow	0.75970828
ZAP70	yellow	0.70602492
ZNF423	yellow	-0.5451109
ZNF683	yellow	0.80490598
ARHGEF15	black	0.74202771
BCL6B	black	0.72843412
CD34	black	0.57444802
CDH5	black	0.91240684
CETP	black	0.32732364
CLEC14A	black	0.798445
CLEC1A	black	0.47670584
CLEC3B	black	0.48452721
DLL4	black	0.71164592
DYSF	black	0.50907896
ECSCR	black	0.65850745
ADGRL4	black	0.78792636
EMCN	black	0.6344319
ESAM	black	0.88813505
FLT1	black	0.59889069
GIPC3	black	0.56361584
GNG11	black	0.4564193
ADGRF5	black	0.73331748
HEYL	black	0.62872314
IL18	black	-0.432235
ITGA1	black	0.68867311
ITGA8	black	0.55962464
ITM2A	black	0.36544663
KDR	black	0.68906573
LILRB5	black	0.36270021
LYVE1	black	0.48028784
MYCT1	black	0.83849578
NOTCH4	black	0.75692109
PCDH12	black	0.79315362
PTPRB	black	0.71131619
RAMP3	black	0.47328039
RGS5	black	0.69387441
RHOJ	black	0.67814327
ROBO4	black	0.90019735
S1PR1	black	0.61616741
SH2D3C	black	0.67245378
TBXA2R	black	0.4295062
TEK	black	0.5732824
TIE1	black	0.64155253
TM4SF18	black	0.8107786
VWF	black	0.72668868
